# Pathway-specific model estimation for improved pathway annotation by network crosstalk

**DOI:** 10.1038/s41598-020-70239-z

**Published:** 2020-08-12

**Authors:** Miguel Castresana-Aguirre, Erik L. L. Sonnhammer

**Affiliations:** Department of Biochemistry and Biophysics, Science for Life Laboratory, Stockholm University, Box 1031, 17121 Solna, Sweden

**Keywords:** Computational models, Databases, Computational biology and bioinformatics, Statistical methods

## Abstract

Pathway enrichment analysis is the most common approach for understanding which biological processes are affected by altered gene activities under specific conditions. However, it has been challenging to find a method that efficiently avoids false positives while keeping a high sensitivity. We here present a new network-based method ANUBIX based on sampling random gene sets against intact pathway. Benchmarking shows that ANUBIX is considerably more accurate than previous network crosstalk based methods, which have the drawback of modelling pathways as random gene sets. We demonstrate that ANUBIX does not have a bias for finding certain pathways, which previous methods do, and show that ANUBIX finds biologically relevant pathways that are missed by other methods.

## Introduction

Improvements in molecular biology have led to an increase in high-throughput data, which typically produces lists of differentially expressed genes or proteins. These lists are useful for identifying genes with important roles in certain conditions. However, more insight about the biological mechanisms is often needed, e.g. which functional gene sets are related to genes in the result list. The study of the relation between a query gene set (differentially expressed gene list) and functional gene sets (pathways) is called pathway enrichment analysis.

Improvements in molecular biology have led to an increase in high-throughput data, which typically produces lists of differentially expressed genes or proteins. These lists are useful for identifying genes with important roles in certain conditions. However, more insight about the biological mechanisms is often needed, e.g. which functional gene sets are related to genes in the result list. The study of the relation between a query gene set (differentially expressed gene list) and functional gene sets (pathways) is called pathway enrichment analysis.

There are four generations of pathway enrichment analysis approaches. Over-representation analysis (ORA) calculates how many genes from a list of genes, extracted based on a threshold or criteria (e.g. differentially expressed genes), are in a certain pathway^1^. Statistical significance is assessed repeating this process with a background list of genes (e.g. all the genes in the microarray). This is known as Gene Enrichment Analysis (GEA) and famous tools like DAVID^2^ use it. Similar but taking into account all the genes in the experiment and the gene expression values, is the Functional Class Scoring algorithms (FCS)^3^, for which known algorithms include Gene Set Analysis(GSA)^4^ and Gene Set Enrichment Analysis (GSEA)^5^. However, both FCS and ORA have limitations. They both consider genes as independent, which is often not true, only taking into account their overlap and not their associations or interactions^6^. Another issue with overlap-based methods is their low coverage since they are heavily dependent on pathway knowledge, which is still incomplete, leading to a high rate of false negatives^7^. Pathway topology-based methods use the same steps as FCS with additional pathway topology information. However, the reliance on gene overlap leads to similar limitations as ORA and FCS.

We could consider the network crosstalk enrichment tools as the fourth generation. They rely on a network, such as a functional association network like Funcoup^8^ or STRING^9^. These networks integrate different experiments from different data types into a single network, providing information about gene to gene functional associations, which is translated into links in the network. With this, limitations such as gene independency and low coverage of overlap-based methods are overcome. Association between two sets is measured in terms of links between them in the network, known as crosstalk. In the past few years different ways to assess enrichment between two gene sets have been published, like NEA^10^, EnrichNet^11^, CrosstalkZ^12^, NEAT^13^, NEArender^14^, BinoX^7^, and GeneSetDP/GeneSetMC^15^. EnrichNet defines a network enrichment score based on network distances between two gene sets using random walks with restart, but is not able to calculate statistical significance of the enrichment. The tools NEA and CrosstalkZ assess significance using statistical tests assuming that crosstalk between non-enriched gene sets is normally distributed, but this is often not the case. Moreover, they rely on network randomizations to obtain null model parameters, which makes them computationally very slow. Computational time is reduced in BinoX, which also applies network randomization but uses the binomial distribution to calculate statistical significance.

The methods NEAT, NEArender and GeneSetDP/GeneSetMC do not use network randomization. NEAT calculates the expected number of links between two gene sets based on their degrees and then uses the hypergeometric distribution to assess statistical significance. NEArender computes the expected number of links in the same way as NEAT, but uses a chi-square test to assess statistical significance. GeneSetDP uses dynamic programming to calculate an exact distribution of the expected number of links to a pathway for a certain gene set size. GeneSetMC does this approximately using Monte-Carlo sampling, which is faster. These two algorithms are however not implemented to allow large scale pathway enrichment analysis.

The null model assumption of NEAT, NEArender, and BinoX is that compared gene sets are expected to behave like random gene sets. For real pathways that are very non-random (e.g. highly intra-connected) this can lead to underestimating the expected level of crosstalk and produce a high false positive rate (FPR). To avoid this, it is important that the method can cope with the non-randomness of pathways. To this end, we have developed a novel network-based pathway enrichment analysis algorithm called ANUBIX (Adaptive NUll distriButIon of X-talk), which is based on scoring random gene sets against real pathways to build its null model. We show that ANUBIX clearly outperforms recent network crosstalk methods like BinoX, NEArender, and NEAT in terms of avoiding False Positives (FP), showing that it can model expected network crosstalk to pathways more precisely.

## Material and methods

Our network-based pathway enrichment analysis tool, ANUBIX, depends on a global functional association network. We used the network Funcoup version 3.0, with a link confidence cutoff of 0.75, containing 12,391 genes and 1,123,873 links. With those genes $\left\{{g}_{1},{g}_{2},...,{g}_{n-1},{g}_{n}\right\}\in S$ and all the pairwise links between them form a symmetric matrix $A$, with dimensions $SxS$ such that:

$$a_{ij} = 1\{ {\text{if}}\; \, g_{i} \;{\text{is }}\;{\text{connected }}\;{\text{to}}\;g_{j} \;{\text{and}}\;i \ne j\} , \;{\text{otherwise}}\;\;\;a_{ij} = 0$$

A gene set $Q$ and a pathway $P$ are a subset of the total number of genes for a certain proteome, such that $\left\{Q,P\right\}\subseteq S$. Notice that $S\subseteq Q$, we can have some genes from the proteome that are not in the network. The crosstalk between $Q$ and $P$ is measured with the degree $k={\sum }_{i\in Q}{\sum }_{j\in P}{a}_{ij}$.

The null model is built based on the expected crosstalk between a random gene set of the same size as the original gene set $Q$ and pathway $P$. Since the network connections are binary, each link is considered as a Bernoulli trial $Y\sim B\left(p\right)$, where $p$ is the probability of observing a link. We also calculate $n=\left|Q\right|\left|P\right|-\left|Q\cap P\right|$, all the possible links between $Q$ and $P$. We count the links each gene from Q has to the pathway $P$, meaning that if two linked genes are in $Q$ and also in the $P$, we count that link twice, boosting the cases where we find overlap. Each of these Bernoulli trials are assumed to be independent and the sum of them follows a binomial distribution.

In the binomial distribution, the mean and variance are defined as $\mu =np$ and $Var=np\left(1-p\right)$, respectively. This means that $\mu \ge Var$, which may not be true when the random variable is overdispersed, leading to an underestimation of its variance^16^.

The beta-binomial distribution has been extensively used as an alternative to handle overdispersed binomial-like random variables^17,18^. Here, the probability of success $p$, is not fixed as it is in the binomial distribution, but follows a beta distribution, *Beta(α, β)* with parameters *α* > 0 and *β* > 0*.*

The marginal distribution of the beta-binomial is described in Eq. (1):

1 $$f\left(k\text{|}n,\alpha ,\beta \right)=\left(\begin{array}{c}n\\ k\end{array}\right)\frac{B\left(k+\alpha ,n-k+\beta \right)}{B\left(\alpha ,\beta \right)}$$

To estimate the optimal parameters of the beta-binomial we use maximum likelihood estimation (MLE)^19^, where the log-likelihood is, Eq. (2):

2 $$\begin{array}{c}l\left(k\text{|}n,\alpha ,\beta \right)=logL\left(k\text{|}n,\alpha ,\beta \right) \, \, \, \, \, \, \, \, \, \, \, \, \, \, \, \, \, \, \, \, \, \, \, \, \, \, \, \, \, \, \, \, \, \, \, \, \, \, \, \, \, \, \, \, \, \, \, \, \, \, \, \, \, \, \, \, \, \, \, \, \, \, \, \\ \, \, \, \, \, \, \, \, \, \, \, \, \, \, \, \, \, \, =log\left(\begin{array}{c}n\\ k\end{array}\right)+logB\left(\alpha +k,\beta +n-k\right)-logB\left(\alpha ,\beta \right)\\ \, \, \, \, \, \, \, \, \, \, \, \, \, \, \, \, \, \, =log\left(\begin{array}{c}n\\ k\end{array}\right)+log\Gamma \left(\alpha +k\right)+log\Gamma \left(\beta +n-k\right)-\\ \, \, \, \, \, \, \, \, \, \, \, \, \, \, \, \, \, \, \, \, \, \, \, \, \, \, \, \, \, log\Gamma \left(\alpha +\beta +n\right)-log\Gamma \left(\alpha \right)-log\Gamma \left(\beta \right)+log\Gamma \left(\alpha +\beta \right)\end{array}$$

The negative log-likelihood is optimized with the Nelder and Mead method^20^. The factorial term in the log-likelihood is removed since it does not depend on the parameters to be optimized. Once we have the beta-binomial parameters $\alpha ,\beta$ of our null distribution we calculate if the crosstalk between $Q$ and $P$ is enriched. The null and alternative hypotheses are:

${H}_{0}$: No more links between $Q$ and $P$ than expected by chance.

${H}_{1}$: More links between $Q$ and $P$ than expected by chance.

**Figure 1:**
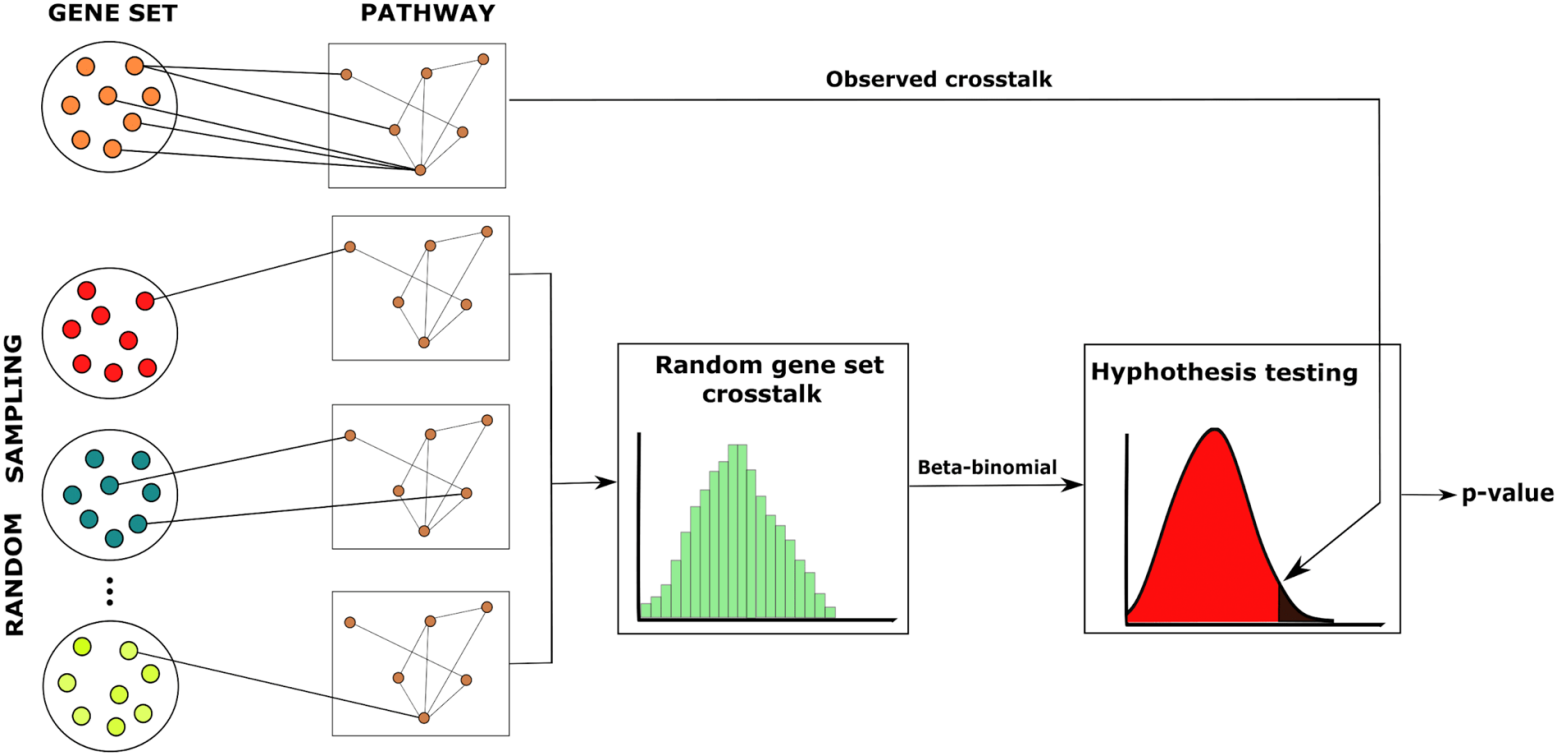
Workflow of ANUBIX. The algorithm assesses the significance of the network crosstalk between a query gene set and a pathway. A null distribution is generated for each pathway to model the expected crosstalk of random gene sets of the same size as the original gene set. This distribution is then fit to a beta-binomial distribution to calculate the probability of reaching at least the number of observed links, or more, between the query gene set and the pathway. Software: Inkscape version 0.91 https://inkscape.org.

Because of the discrete nature of the null distributions, ordinary p-values are conservative, and therefore mid p-values were used^21,22^. Mid p-value is defined as half the probability of the observed statistic plus the probability of observing more extreme values^22^. The workflow of the ANUBIX algorithm is depicted in Fig. 1.

It is important to point out that the network-based approaches ANUBIX, NEAT, NEArender, and BinoX test three different types of null hypothesis. ANUBIX, which takes only enrichment into account, computes a one tailed test. NEAT computes two one-tailed tests, for enrichment and depletion, and takes the minimum p-value of them multiplied by 2 to emulate a two-tailed test. BinoX and NEArender compute both enrichment and depletion but only perform one one-tailed test since the hypothesis test changes depending on whether the observed number of links is above or below the expected crosstalk.

### Pathways

To generate the false positive and true positive benchmarks we used 288 KEGG (v70.1)^23^ pathways and 398 REACTOME (v62)^24^ pathways for *Homo sapiens*. REACTOME pathways have a deep hierarchical structure, including many small pathways on the lower levels that are very specific. To reduce Reactome’s specificity we resolved its hierarchy by collapsing lower level pathways below a certain pathway size to their parents until obtaining an average pathway size similar to KEGG pathways, 80 genes per pathway.

### Performance measures

In the FP benchmark, we generated 10,000 random gene sets and tested them against KEGG and REACTOME pathways. To make these gene sets representative of real experiments, we took the average size of MSigDB^25^ gene sets, which is 110 genes.

In the True Positive (TP) benchmark, we bisected the KEGG pathways and REACTOME pathways into two parts. Each part gets a similar number of genes and links^7^. To be able to benchmark GEA we emulated some overlap between the two bisected parts. This overlap corresponded to the average overlap between the 2,392 MSigDB gene sets and the pathway, measured individually for each of the pathways in KEGG and REACTOME.

Correction for multiple hypothesis testing was done using the Benjamini–Hochberg procedure^26^.

### Stability

Our null distributions are based on random sampling. We take random samples of genes from the genome. This stochastic procedure makes the null distributions different every time they are generated. Since the p-values are computed from the null distribution, their values may change. To analyze stability, we generated the null distribution 100 times for the crosstalk between the same gene set to the same pathways, for increasing numbers of random samples. For each sample size, we computed the coefficient of variation (CV), which is the ratio between the standard deviation (SD) and the mean. We required a CV lower than 2% to limit the dispersion of the mean of the null distribution, and this was reached at 2000 random samples. Once the number of random samples were chosen, we measured how much the p-values were varying in each run. For that we ran a randomly selected MSigDB gene set 100 times. To compute the 95% confidence interval of the p-values, we used the central limit theorem and applied normal distribution statistics to compute them.

### Used programs

ANUBIX: https://bitbucket.org/sonnhammergroup/anubix

BinoX: https://bitbucket.org/sonnhammergroup/binox

NEAT: https://cran.r-project.org/web/packages/neat/neat.pdf

NEArender: https://cran.r-project.org/web/packages/NEArender/NEArender.pdf

GeneSetDP: https://github.com/statisticalbiotechnology/genesetdp

## Results

**Figure 2:**
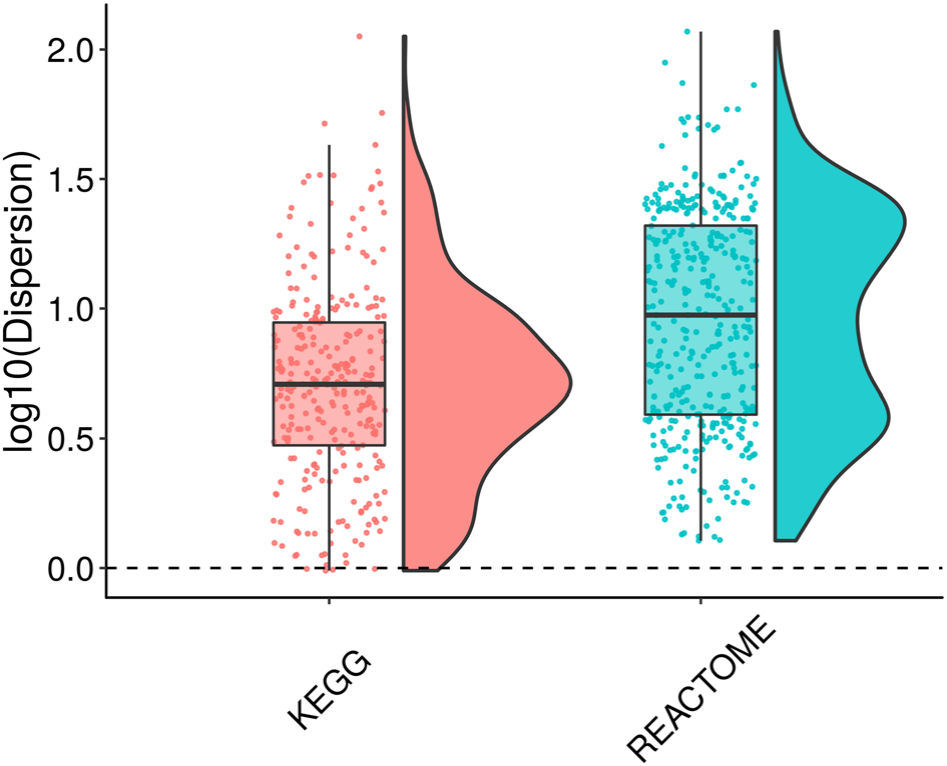
Overdispersion of KEGG and REACTOME pathways null distributions when sampling 2000 random gene sets of size 110 from the proteome. The dispersion for each pathway is calculated as the ratio between the variance and the mean of the crosstalk null distribution. For each pathway database we illustrate the dispersion values through a boxplot and also by showing the dispersion distribution. Software: R version 3.4.3 https://www.r-project.org/.

To correctly assess the statistical significance of an observed network crosstalk between two gene sets, e.g. one experimental gene set and one known pathway, it is paramount that the null distribution appropriately models the crosstalk of random query gene sets. Note that it is not necessarily appropriate to assume that the pathway gene set behaves like a random gene set, i.e. the null distributions need to model crosstalk between random query gene sets versus real pathway gene sets. It is also paramount to model the expected crosstalk distribution with an appropriate distribution. Previous methods, such as BinoX or NEAT, use binomial and hypergeometric distributions respectively, which are not appropriate for overdispersed distributions, since they do not allow the variance of the distribution to be greater than the mean. To showcase this, we generated null distributions for KEGG and REACTOME pathways by sampling 2,000 gene sets of size 110 from the proteome. In Fig. 2 we show the dispersion for each pathway as the ratio between the variance and the mean of the crosstalk null distribution. We observe that almost all of these distributions suffer from overdispersion, meaning that the variance of the distribution is greater than the mean. Therefore, statistical models that cannot cope with overdispersion are not appropriate to model the null distribution of most pathways.

**Figure 3:**
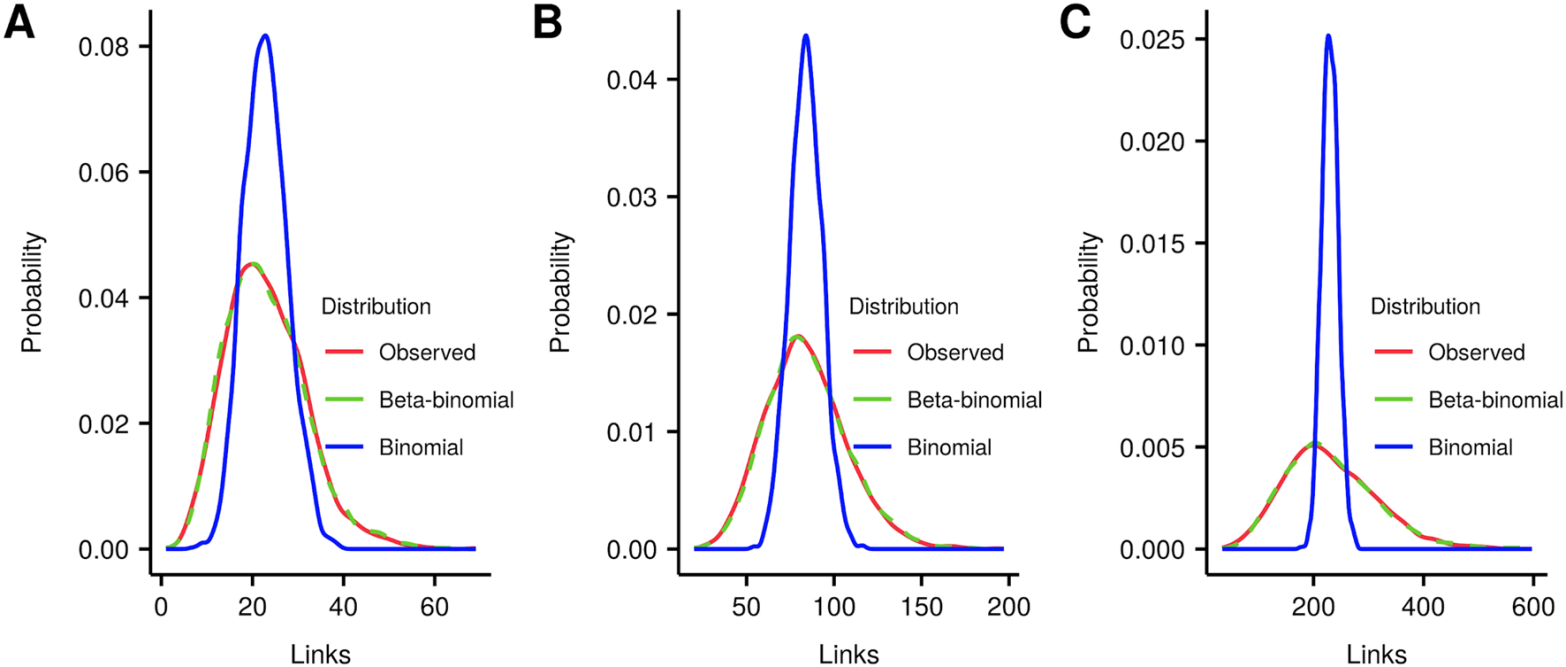
Observed crosstalk distribution fit with binomial and beta-binomial distributions. 2000 random gene sets of size 110 were used to generate a null distribution of crosstalk to the (**A**) “Beta-alanine metabolism”, (**B**) “Prostate cancer”, and (**C**) “Alzheimer’s disease” pathways. Beta-binomial shows a much better fit to the observed link distribution than the binomial. Software: R version 3.4.3 https://www.r-project.org/.

To visualize the overdispersion in detail we chose 3 pathways that are in different quartiles of the dispersion distribution. We show their null distributions in Fig. 3. Figure 3A shows the “Beta-alanine metabolism” pathway, whose dispersion value is in the first quartile. Figure 3B shows the “Prostate cancer” pathway, with a dispersion in the second quartile, and Fig. 3C shows the “Alzheimer’s disease” pathway with a dispersion in the fourth quartile. The high variance relative to the mean gives a very poor fit with the binomial distribution, yet the beta-binomial distribution gives a very good fit. This underestimation of variance by the binomial distribution would lead to many false positives. With a few pathways there is no overdispersion in the data, but these can fit a beta-binomial equally well as a binomial.

### Benchmark for false positives

**Figure 4:**
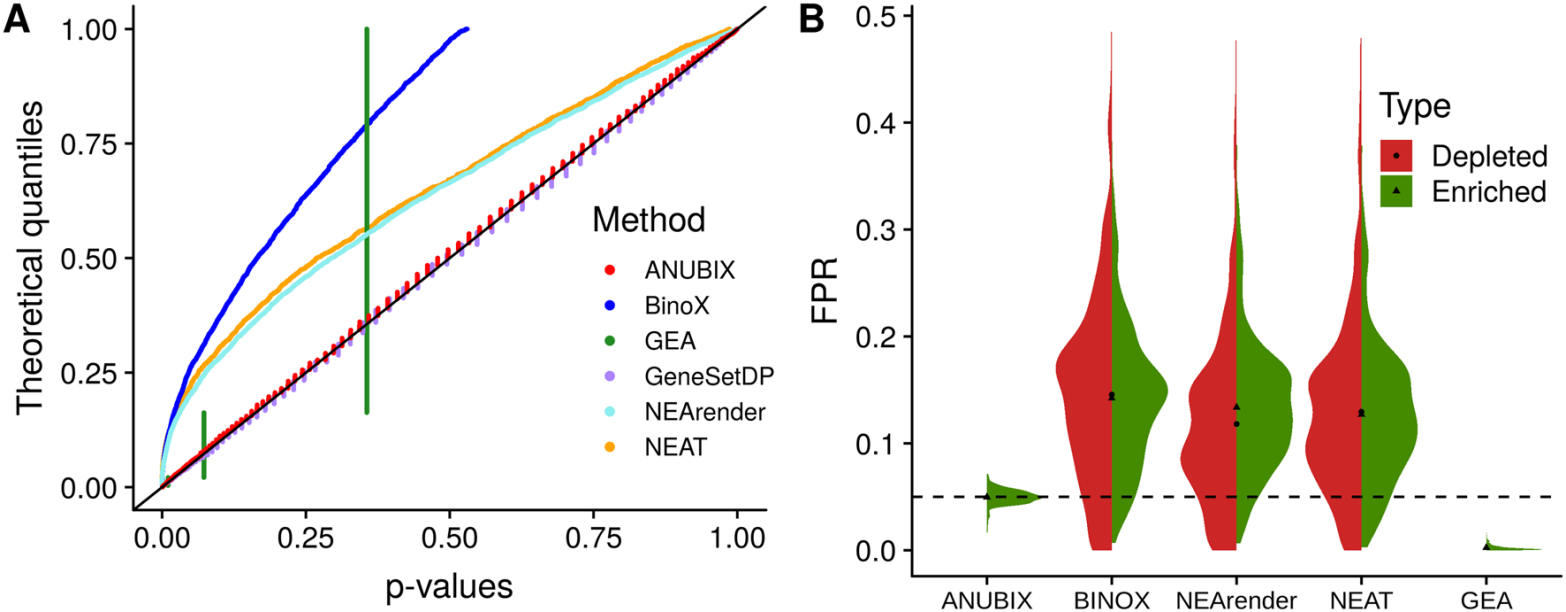
P-value uniformity test of ANUBIX, Binox, GEA, GeneSetDP, NEArender and NEAT. 10,000 random gene sets of 110 genes were tested for crosstalk enrichment against the KEGG pathway “Prostate cancer” (**A**). Reported p-values are plotted against theoretical quantile (rank). A perfect method should adhere to the diagonal. (**B**) Distributions of the FPR for all KEGG and REACTOME pathways tested with ANUBIX, BinoX, NEArender, NEAT and GEA. Green distribution for enriched tests and red distribution for depleted. The dashed line at FPR = 0.05 denotes the expected FPR level. The black triangle and circle represent the mean FPR for enrichment or depletion respectively. Software: R version 3.4.3 https://www.r-project.org/.

Since the null model in ANUBIX is based on random gene sets we expect the p-value distributions when tested with random query gene sets to behave uniformly for any pathway. For almost all pathways we observed a virtually perfectly uniform distribution when plotting ANUBIX p-values of 10,000 random gene sets against each KEGG pathway (full results at Supplementary Fig. 1). A few pathways deviated somewhat from uniform, which is the result of the beta-binomial fit not being able to model the null distribution with enough precision. A second type of deviation from perfect uniform distribution is caused by staggering of observed p-values. This is relatively frequent and arises because the support of the test statistics is limited to a few values and therefore unavoidable. We also generated the p-value distributions for 10,000 gene sets of size 50 and size 200 against each KEGG pathway (Supplementary Fig. 2 and 3 respectively), which gave similar results. However, some pathways seem to be affected by the size of the gene set. ANUBIX was compared to the top network-based methods BinoX, NEAT and NEArender, and a recently published method GeneSetDP. For comparison we also tested a popular overlap-based pathway enrichment method, GEA. Because GeneSetDP and GenesetMC are too computationally heavy for large scale analysis, we first tested all the gene sets against one individual pathway. We only used GeneSetDP because GeneSetMC produces similar p-values. P-values were plotted versus quantiles of a uniform distribution (0,1). For an unbiased method, the p-values would lie on the diagonal $y=x$. Figure 4A shows that for the “Prostate cancer” pathway. P-values of ANUBIX adhere to the diagonal much better than for BinoX, NEAT, NEArender and GEA, while performing equally well as GenesetDP.

For crosstalk to random gene sets, we expect ~ 5% of the p-values to be lower than 0.05. However, for the “Prostate cancer” pathway, BinoX had 26.4% of its p-values lower than 0.05, NEAT 21.2% and NEArender 20.9%. GEA, whose coverage is small^7^, had 0.2% of its p-values below 0.05, and highly discrete, taking on only four possible values for “Prostate cancer” due to few overlapping genes. ANUBIX and GeneSetDP find a correct fraction of the p-values with 5.4% and GeneSetDP 5.2% under 0.05, respectively.

We also ran ANUBIX, BinoX, NEAT, NEArender, and GEA for the 10,000 random gene sets against all pathways in the KEGG database and REACTOME database. Full results in Supplementary Data 1 and Data 2 respectively. GeneSetDP was not included as it is not implemented to run at a large scale. NEAT, NEArender and BinoX can also give statistical significance when gene sets have fewer links to a pathway than expected by chance, known as depletion. To make a more consistent benchmark where all methods can be compared equally we only considered enrichment, and depleted pathways were treated as non-significant. The average FPR for all KEGG pathways was 5.1% with ANUBIX, 13.3% with BinoX, 11.2% with NEAT, 12.0% with NEArender, and 0.4% with GEA. For REACTOME almost the same FPR values were obtained (ANUBIX 4.9%, BinoX 14.9%, NEAT 13.8%, NEArender 14.3% and GEA 0.2%). Roughly the same FPR levels came from significant depletions for BinoX, NEAT and NEArender. However, the averaging of the FPR levels for all pathways does not show the real problem of these methods. Some pathways could give very non-conservative p-values while other pathways could give very conservative p-values. To show how each method performs for each of the pathways we plot the distribution of the FPR (fraction of p-values below 0.05) for each pathway as violin plots in Fig. 4B. Since GEA and ANUBIX cannot test for depletion they only have the enriched case. A perfect method would have all points close to the dashed line at FPR = 0.05. ANUBIX produces FPR values close to this line, meaning that the model is robust. GEA greatly underestimates FDR and produces almost no false positives, but this leads to very poor sensitivity as shown below. NEArender, NEAT and BinoX, produce similar FPR distributions that are very spread out, i.e. the FPR tends to be very different for different pathways. For the 10,000 tests performed per pathway, some pathways reach an FPR of 0.4 for enriched cases and similar for depleted. Summing these two can lead to a total FPR above 0.8 if we take both enriched and depleted cases into account, which is very non-conservative. The plot also shows that for some pathways these methods are overly conservative, giving considerably lower FPR than they should. In other words, methods like BinoX, NEAT and NEArender have a huge variation in the quality of their p-values depending on the pathway under study.

**Figure 5:**
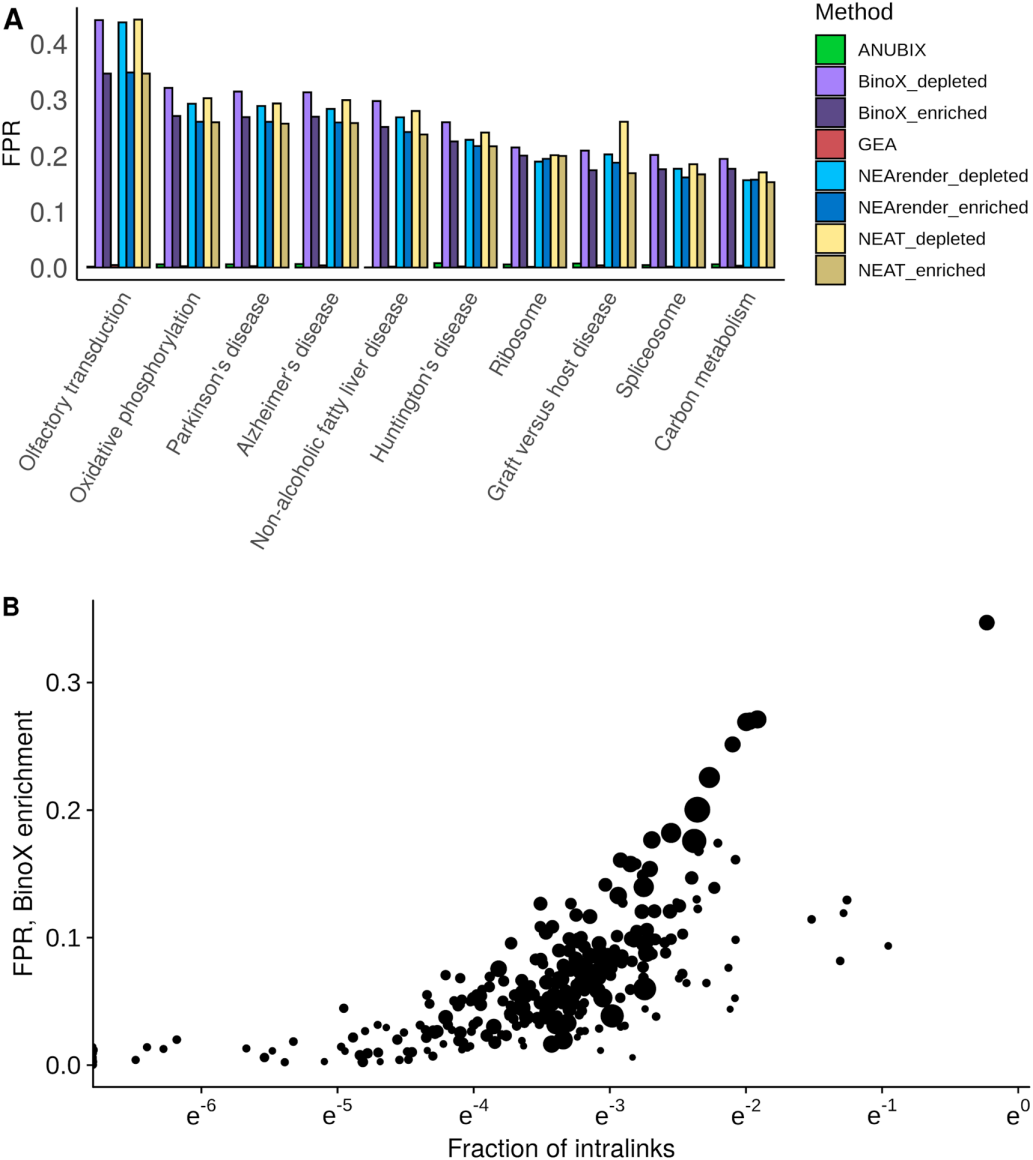
Analysis of why certain pathways are very prone to produce false positives. 10,000 random gene sets of 110 genes were tested independently for crosstalk enrichment against the KEGG pathways. (**A**) The top ten pathways that produce the highest false positive rate (FPR) with BinoX, and the FPR obtained with other methods. (**B**) Fraction of intralinks for each of the KEGG pathways against FPR. The size of the point reflects the total number of links in each pathway. Software: R version 3.4.3 https://www.r-project.org/.

BinoX is implemented in a web server, called PathwAX^27^, where users can submit a query gene set to test for network crosstalk enrichment. By analogy, we studied false positive rates assuming independence between gene sets, where each user submits a single gene set, i.e. multiple testing correction is only performed for number of pathways each query is compared to. 10,000 random gene sets were used against the KEGG database. A FDR threshold of 5% was used and enrichment and depletion were grouped separately as shown in Fig. 5A. The top 10 pathways with highest FPR for BinoX were plotted (full results in Supplementary Data 3), all having a highly non-conservative behaviour for BinoX, NEAT and NEArender. Every time a user submits a random gene set, the chance of getting one of these pathways is very high, on average 40% if we take both enriched and depleted cases into account. In contrast, ANUBIX and GEA have less than 1% FPR. We observed a very high correlation between per-pathway FPR values for BinoX, NEAT and NEArender, above 0.98 for each pairwise comparison. This indicates that the pathway enrichment analysis results obtained with these methods are highly similar. They all had low Pearson correlation to ANUBIX, with 0.06 for BinoX, 0.005 for NEAT, and 0.08 for NEArender. The corresponding Spearman correlations were 0.24, 0.22, and 0.11.

As for the pathways, we noticed that there is a high overlap between some of them. For instance, the “Alzheimer’s disease” and “Parkinson’s disease” pathways share 43.3% of their genes. The “Alzheimer’s disease”, the “Parkinson’s disease” and the “Huntington’s disease” pathways have 32% of the genes in common from the union between them. Further, the “Oxidative phosphorylation”, the “Non-alcoholic fatty liver disease”, the “Alzheimer’s disease”, the “Parkinson’s disease” and the “Huntington’s disease” have 20% of the genes in common from the union between them. Therefore, if there is significant crosstalk to one of them, crosstalk to the other pathways is very likely. The high dependency between some pathways points to opportunities for further improvement of pathway definitions. Further exploration was performed in these pathways’ topologies to understand their tendency to generate many FPs.

We computed the fraction of intralinks for each pathway, as the ratio between the number of internal links and the total number of links. We plotted this ratio against the FPR (Fig. 5B). A higher fraction of intralinks means that more links are within the pathway than to the outside, suggesting a more isolated pathway. The Spearman correlation coefficient between the fraction of intralinks and FPR for BinoX was 0.79, indicating that the fraction of internal links plays a major role in causing false positives. This dependence is also observed with NEAT, with a correlation of 0.82, and with NEArender at 0.83. However, ANUBIX had a correlation of only 0.12 and GEA 0.34. This indicates that methods like NEAT, NEArender, and BinoX cannot deal properly with pathways that are clearly not random and behave more as isolated communities.

Additionally, we calculated the number of maximal cliques each of the KEGG pathways has and we observed a correlation with the FPR for BinoX, with a spearman correlation of 0.71. These maximal cliques were computed using the igraph package in R. We considered cliques as all complete subgraphs and a clique is considered maximal if we cannot add more nodes to it. This indicates that the higher the number of maximal cliques in a pathway, meaning a less random pathway in terms of topology, the higher the FPR is.

### Benchmark of true positives

**Figure 6:**
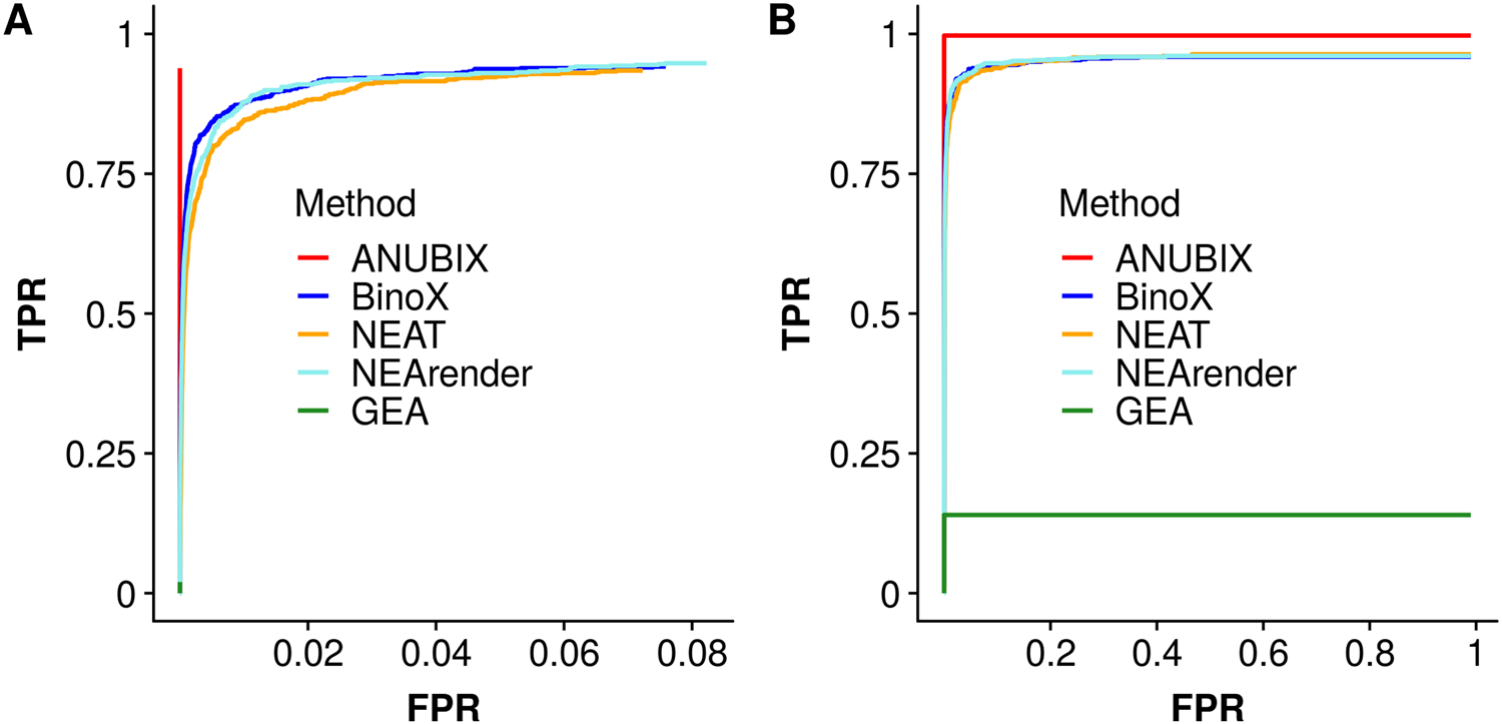
Receiver Operating Characteristic (ROC) curve. For the TP tests, each KEGG and REACTOME pathway is divided into two and a TP is interpreted as the crosstalk between two parts from the same pathway. For the FP tests, 10,000 random gene sets of size 110 are tested for enrichment against KEGG and REACTOME pathways. (**A**) ROC curve for only the significantly enriched tests (FDR < 0.05). (**B**) ROC curve for all enriched tests. Software: R version 3.4.3 https://www.r-project.org/.

Besides a correct FPR, it is also important to verify that the power of the method is sufficient for a high true positive rate (TPR). To this end we devised a benchmark by splitting each KEGG and REACTOME pathway into two parts and then measured each method’s ability to reconnect these parts. The splitting into parts included giving an amount of gene overlap between the two parts, emulated based on the average overlap between MSigDB gene sets and KEGG and REACTOME pathways. We compared the methods by their Receiver Operating Characteristic (ROC) curves. Figure 6A shows only the tests that are statistically significant, FDR < 5%, and only considering enrichment. ANUBIX has a TPR of 94.0% of the enrichment tests as significant without having any FP. BinoX has a TPR of 94.2% with 7.6% FPR, NEArender a TPR of 94.8% with 8.2% FPR, and NEAT a TPR of 93.4% with 7.2% FPR. GEA, whose coverage is low, gives only 1.5% TPR and no FPs. Figure 6B shows the ROC curve for all the enriched tests performed, also including insignificant results. This shows the coverage of each method. ANUBIX recovers 99.4% of the TP tests without suffering any FPs. BinoX, NEArender and NEAT have similar curves, recovering 96.2%, 95.9% and 95.5% of the enriched TP tests respectively. GEA can here maximally find 14.1% of the TP tests, since only those tests have some gene overlap. This benchmark shows that GEA has very low coverage of what it can potentially find. We note that the maximal TPR obtained by GEA corresponds to the amount of significantly enriched crosstalks obtained when running all of MSigDB against KEGG pathways (see Pathway annotation of MSigDB gene sets).

### Stability and robustness

**Figure 7:**
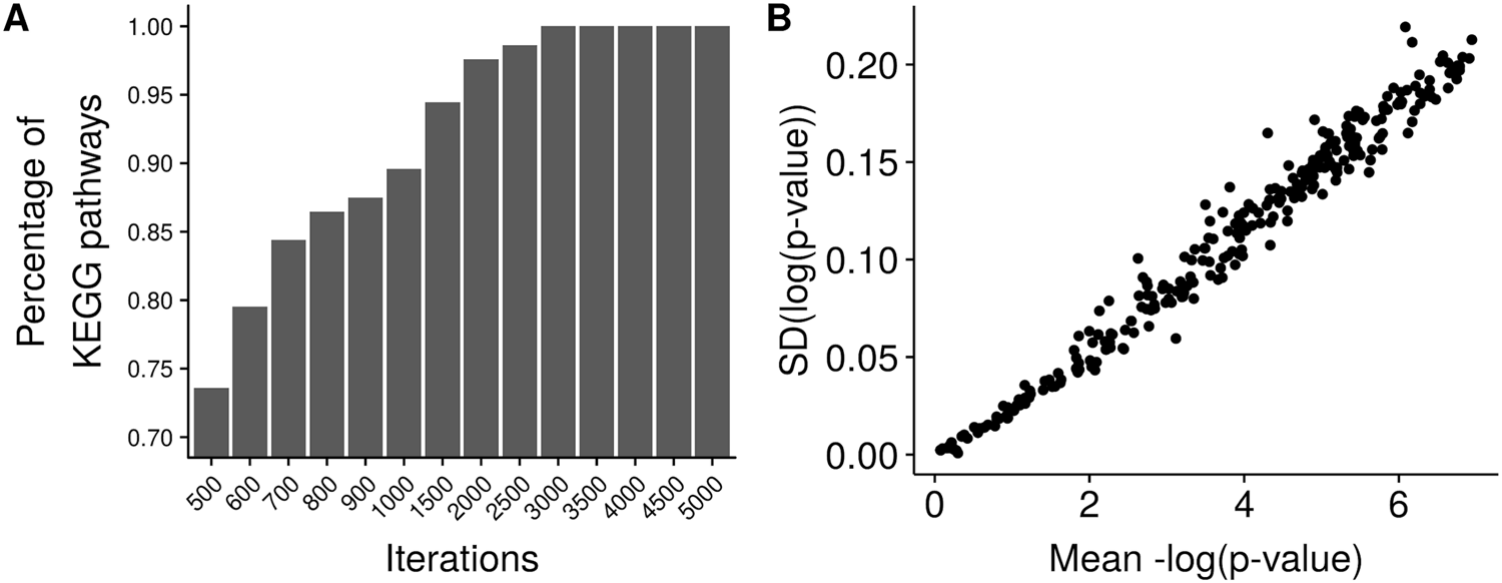
Stability analysis of ANUBIX. (**A**) Fraction of KEGG pathways with Coefficient of variation (CV) below 2% for different number of iterations. (**B**) ANUBIX p-values are stable—their variance is low, and proportional to the magnitude of the p-value. A randomly chosen MSigDB gene set, DAIRKEE_CANCER_PRONE_RESPONSE_BPA, was run 100 times against KEGG pathways. Standard deviation of the log(p-value) is plotted against the mean-log(p-values) for each pathway. Software: R version 3.4.3 https://www.r-project.org/.

Considering that the null distributions are based on random sampling, we studied the number of iterations required to reach a coefficient of variation (CV) of 2%. Figure 7A shows how many pathways pass that threshold depending on different amounts of random samples. 98% of the pathways had a CV lower than 2% when using 2,000 random samples to model the null distribution. To verify that this number of random samples is sufficient for every pathway, we computed the enrichment of one randomly selected MSigDB gene set to all KEGG pathways 100 times. The null distributions are thus generated 100 times for each pathway and we would expect some changes in the p-values between runs. Figure 7B shows the standard deviation of the p-values. We observe that the p-values almost did not vary, showing that 2,000 random samples are enough. Moreover because of sampling, the p-value is not an exact p-value but a point estimate of it, we also provide with the 95% confidence interval of each of the p-values (Supplementary Data 4).

### Compute time

**Figure 8:**
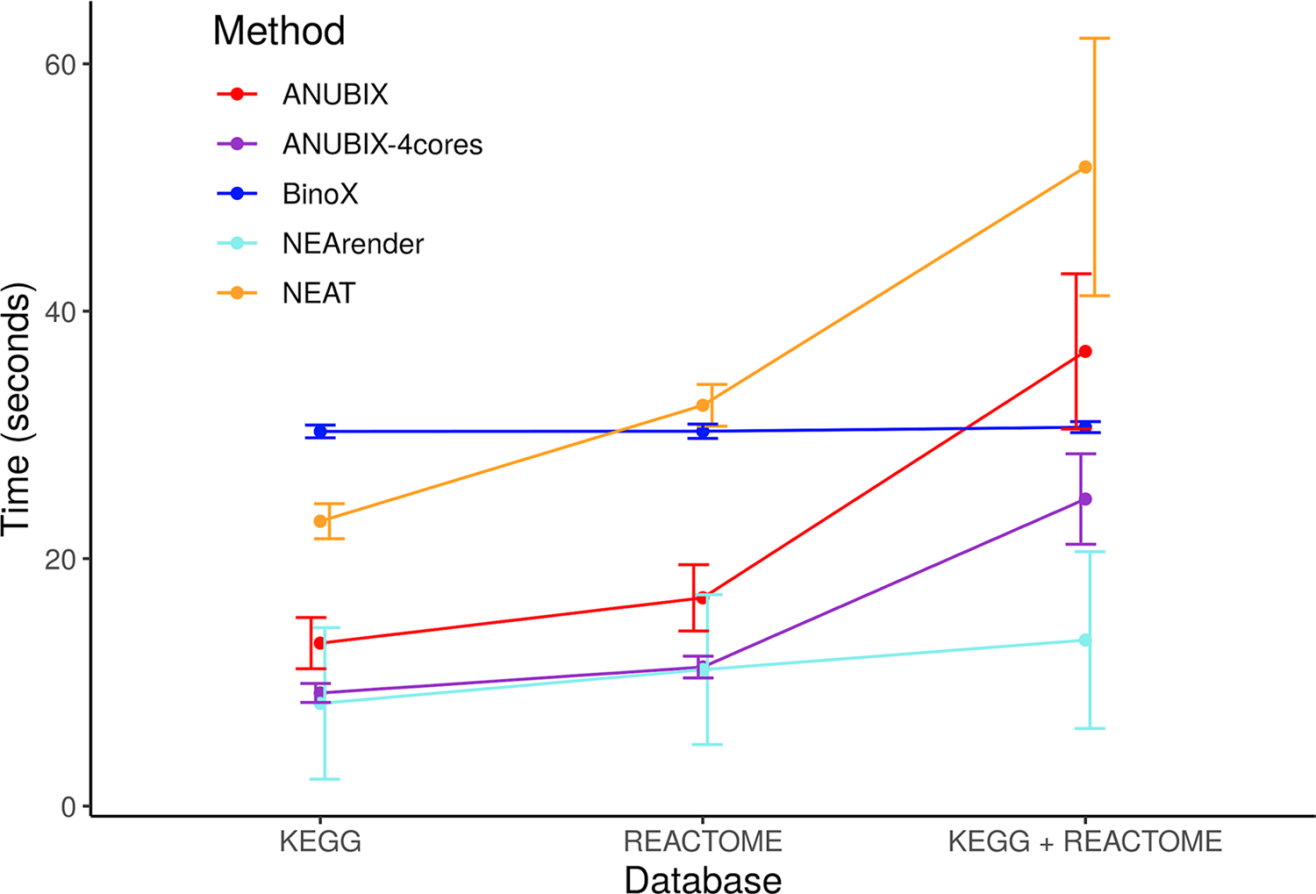
Compute time when running a random experimental gene set from MSigDB. 100 different gene sets were tested against KEGG, REACTOME and KEGG plus REACTOME pathways, for each of the network-based methods. Since ANUBIX allows parallelization we also added another run with 4 cores. The error bars show the variability in compute time for each of the methods in each of the databases. The BinoX precomputation step is not included since it takes 22 min. Software: R version 3.4.3 https://www.r-project.org/.

Our method relies on random sampling to model the null distribution, which makes ANUBIX computationally intensive. To benchmark its speed we did 100 runs, each time with a randomly chosen biological gene set extracted from MSigDB against KEGG, REACTOME, and KEGG plus REACTOME. We measured the compute time for each of the network-based methods, see Fig. 8. With this benchmark we can show that ANUBIX is fast when running single gene sets. One should take into account that ANUBIX and BinoX need a precomputation step before running the actual analysis. However, the ANUBIX precomputation step takes around 2 s whereas in BinoX it takes around 22 min. To compute the randomized network for BinoX, we used 150 iterations. A drawback for ANUBIX compared to methods like BinoX or NEAT is that the computation time for large scale analyses take more time. For instance, the time required to compute the large scale pathway annotation study for the 2392 MSigDB gene sets against KEGG pathways took 150 min for ANUBIX using 4 cores, 90 min for NEArender, 28 min for BinoX, and 18 min for NEAT. Compute times were measured on an i7-7700 CPU 3.60 GHz with 32 Gb RAM.

### Pathway annotation of MSigDB gene sets

We carried out a large-scale pathway analysis study by running 2392 MSigDB gene sets against 288 KEGG pathways using ANUBIX, BinoX, NEAT, NEArender, and GEA. Full results are in Supplementary Data 5. In total 688 896 crosstalk tests were done per method, and to get a more fair comparison between different methods we only considered enriched crosstalk, considering that ANUBIX and GEA can only test for enrichment.

NEArender, BinoX, and NEAT found the highest number of significantly (FDR < 0.05) enriched crosstalks, with 28.75%, 27% and 26.4% of all pairs respectively, followed by ANUBIX with 21.1% and GEA with 1.3%. Many MSigDB gene sets thus appear to have a high occurrence of pathway enrichments. Even if we do not know whether those enrichments are TPs or FPs, we show above (Figs. 4 and 5A) that BinoX, NEArender and NEAT are prone to produce FPs.

**Figure 9:**
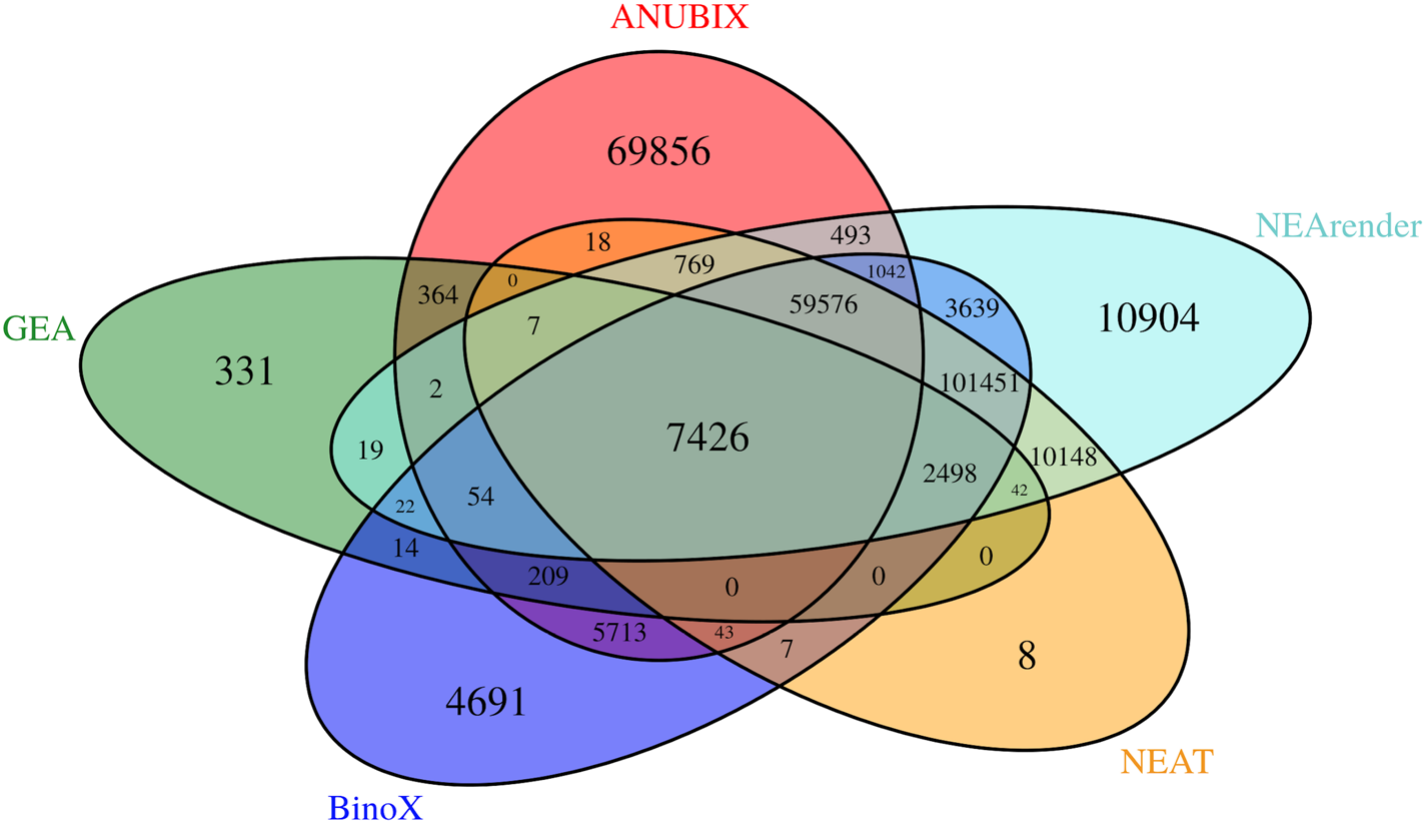
KEGG pathway annotation for 2392 MSigDB gene sets with five methods. The Venn diagram shows the number of shared pathway annotations at FDR < 0.05. Note that ANUBIX finds a high number of unique annotations. Software: R version 3.4.3. https://www.r-project.org/.

The Venn diagram in Fig. 9 shows that the overlap between BinoX, NEAT and NEArender is very high, having 81.9% of their significant pathway annotations in common. This was expected since all these methods consider pathways as random. The overlap is even higher between NEAT and NEArender, 91.8%, because they compute the expected number of links between sets in identical ways. Even though the number of significant annotations by ANUBIX is lower, we show that 47.9% of its annotations are unique.

An example of annotations unique to ANUBIX is the gene set RODRIGUES_THYROID_CARCINOMA_POORLY_DIFFERENTIATED_UP, for which only ANUBIX identifies specific pathways such as “Thyroid cancer” (q-value = 1.0 × ${10}^{-23}$), but also more general cancer pathways, such as “Pathways in cancer” (q-value = 2.8 × ${10}^{-41}$). Further, only ANUBIX found “thyroid hormone signaling” (q-value = 5.3 × ${10}^{-28}$) and “thyroid hormone synthesis” (q-value = 1.1 × ${10}^{-28}$), which is reasonable since it has been demonstrated that anaplastic thyroid carcinomas lose the most characteristic thyroid cellular function, which is the synthesis of T4 and T3 hormones^28^. The tumor suppressor P53 has been found to be mutated in poorly differentiated thyroid carcinoma^29^, and this was supported by ANUBIX with a significant finding of the “p53 signalling” pathway (q-value = 4.3 × ${10}^{-56}$), yet was not found by the other network-based methods. Finally, the “MAPK signaling” pathway (q-value = 5.9 × ${10}^{-20}$) shows a key role in the genesis and progression of a substantial proportion of papillary tumours^30^. For this gene set, none of these pathways were found by any of the other methods, except GEA that found “p53 signalling” pathway with a q-value of 1.4 × ${10}^{-03}$.

Another example is the gene set GRADE_COLON_CANCER_UP. ANUBIX is the only method that finds expected pathways such as, “Colorectal cancer” , “Pathways in cancer” or “microRNAs in cancer'', with q-values of 2.5 × ${10}^{-56}$, 3.3 × ${10}^{-64}$, and 4.8 × ${10}^{-53}$ respectively. Another pathway found only by ANUBIX that is a key driver in almost all colorectal cancers is the “WNT-signaling pathway” (q-value = 7.5 × ${10}^{-55}$)^31^. It also uniquely found two other signalling pathways that generally are dysregulated in cancer, “p53 signalling” and “RAS-signaling” with q-values of 3.5 × ${10}^{-48}$ and 5.8 × ${10}^{-43}$ respectively^32^.

## Discussion

Here we present ANUBIX, a novel network-based pathway enrichment method, which focuses on modelling the expected crosstalk between a gene set and a pathway. We prove how important it is to have an accurate model that can correctly treat real pathways. Users working with pathway enrichment analysis tools are expecting to find out whether their gene sets have a relation to certain pathways that is not expected by chance. To achieve this, ANUBIX keeps the properties of each pathway intact to precisely estimate the expected level of crosstalk between a query gene set and each individual pathway. In contrast, previous methods such as BinoX, NEArender and NEAT generalize the statistical properties of crosstalk for all pathways, and are therefore unable to adapt to specific properties of individual pathways which may be highly non-random. BinoX uses network randomizations to assess the expected crosstalk between a gene set and a pathway, while NEArender and NEAT assume that pathways behave like random gene sets. However, this ignores important properties of the known biological pathways. As a result, the null distributions used by BinoX, NEArender and NEAT are not suitable to handle crosstalk to certain pathways, especially the pathways in Fig. 5A, where they produce a high FPR for random gene sets. This means that a user is likely to get some of those pathways as significant enrichments even though a random gene set is submitted. Almost all pathways showed overdispersed crosstalk distributions for random query gene sets, and hence violate the model assumptions of BinoX, NEArender, and NEAT, leading to high FPR.

We have analyzed the reliability of different methods for crosstalk of random gene sets against all KEGG and REACTOME pathways. For each pathway one would expect ~ 5% of the p-values to be under 0.05. ANUBIX displayed excellent reliability, with pathways close to 5%. In contrast, BinoX, NEAT and NEArender had highly variable performance between pathways. In general, these methods have non-conservative p-values, but we show that for some pathways they are actually too conservative. This shows how sensitive these methods are to pathway properties. Pathways should not be treated as random gene sets, and as shown in Fig. 5B there is a correlation of 0.79 between the FPR of Binox and the fraction of intralinks. The same dependency is observed for NEAT with a correlation of 0.81, and for NEArender at 0.83. This suggests that these methods perform worse when the pathways are more isolated communities. In contrast, ANUBIX only had a correlation of 0.12, showing almost no bias towards certain pathways. Since ANUBIX can handle random gene sets well, as demonstrated in the benchmark, it gives to the user a higher confidence of obtaining reliable results. Further, ANUBIX not only gives a good accuracy for random gene sets together with a perfect specificity, but its TPR competes with previous methods like BinoX, NEArender and NEAT. Because ANUBIX’ model assumption keeps the properties of each pathway intact, it can discover many new pathway annotations that are not found by any of the previous methods. As shown in Fig. 9, 47.9% of the significant MSigDB annotations found by ANUBIX were unique.

Compute time analysis showed that even though ANUBIX is fast for single runs, it scales linearly with the number of query gene sets. In contrast, methods like BinoX and NEAT have a high fixed computation cost regardless of the number of query gene sets but a low cost per gene set, which makes them relatively faster for large batch comparisons.

GenesetDP/GeneSetMC show an equally good accuracy in terms of FPR as ANUBIX for the pathway tested in Fig. 4A. They keep the biological properties of the pathways and they focus on the gene sets the user inputs. However, no large scale benchmark of FPs or TPs was possible since these algorithms are not implemented to allow large scale analyses.

For comparison we also benchmarked GEA, which is implemented in DAVID, a popular overlap-based pathway enrichment analysis tool. While it was not found to have a high FPR for random gene sets, it suffers from poor sensitivity (TPR) which is caused by its dependency on overlapping genes. The high false negative rate may be explained by the fact that GEA only relies on overlap between sets, while network-based methods use a network, which gives them a much richer source of information. Moreover, GEA as implemented in DAVID uses the EASE-score^33^, which is a conservative modification of Fisher’s exact test, and requires an overlap of at least 2 genes to perform the test.

In conclusion, we show that ANUBIX substantially improves the quality of pathway annotation compared to state of the art network-based methods. Existing state of the art network-based methods have high false positive rates and a bias to find certain pathways, which are both eliminated by the ANUBIX algorithm, that also still has a true positive rate that competes with previous network-based methods. We show that ANUBIX is able to find a large amount of biologically relevant pathways that are not found by other methods.

## Supplementary information

Supplementary Figures.

Supplementary Data.

Supplementary Information.

## Data Availability

The 2392 MSigDB gene sets were taken from the C2.CGP (chemical and genetic perturbations) v3.0 collection, available at https://software.broadinstitute.org/gsea/msigdb/download_file.jsp?filePath=/resources/msigdb/3.0/msigdb_v3.0_files_to_download_locally.zip. The 288 human pathways were extracted from KEGG release 70.1 via https://www.kegg.jp/kegg/rest/keggapi.html. The Reactome pathways were gathered from https://reactome.org/download/current/Ensembl2Reactome_All_Levels.txt and the pathway hierarchy from https://reactome.org/download/current/ReactomePathwaysRelation.txt. We also used the human functional association network, Funcoup v3.0 available at https://funcoup.sbc.su.se/archive/FC3.0/. The source code for ANUBIX is available at https://bitbucket.org/sonnhammergroup/anubix/src/master/R/. All scripts and data are available at https://bitbucket.org/sonnhammergroup/anubix/src/master/anubix_benchmark/.
