# Supplementary material for: Pathway-specific model estimation for improved pathway annotation by network crosstalk
Source: Sci Rep. 2020 Aug 12;10:13585. doi: 10.1038/s41598-020-70239-z (PMC7423893; doi:10.1038/s41598-020-70239-z)
Supplement: Supplementary file 1 — Supplementary Figures. [file 41598_2020_70239_MOESM1_ESM.pdf]

Pathway-specific model estimation for improved pathway annotation by network crosstalk

*Miguel Castresana-Aguirre, Erik L.L. Sonnhammer**

Department of Biochemistry and Biophysics, Stockholm University, Science for Life Laboratory, Box 1031, 17121 Solna, Sweden,
*To whom correspondence should be addressed: erik.sonnhammer@dbb.su.se

Supplementary Figure 1: 10000 random gene sets of size 110 are run against KEGG pathways. P-value uniformity test of ANUBIX. Reported p-values are plotted against theoretical quantile (rank). Software: R version 3.4.3 https://www.r-project.org/.

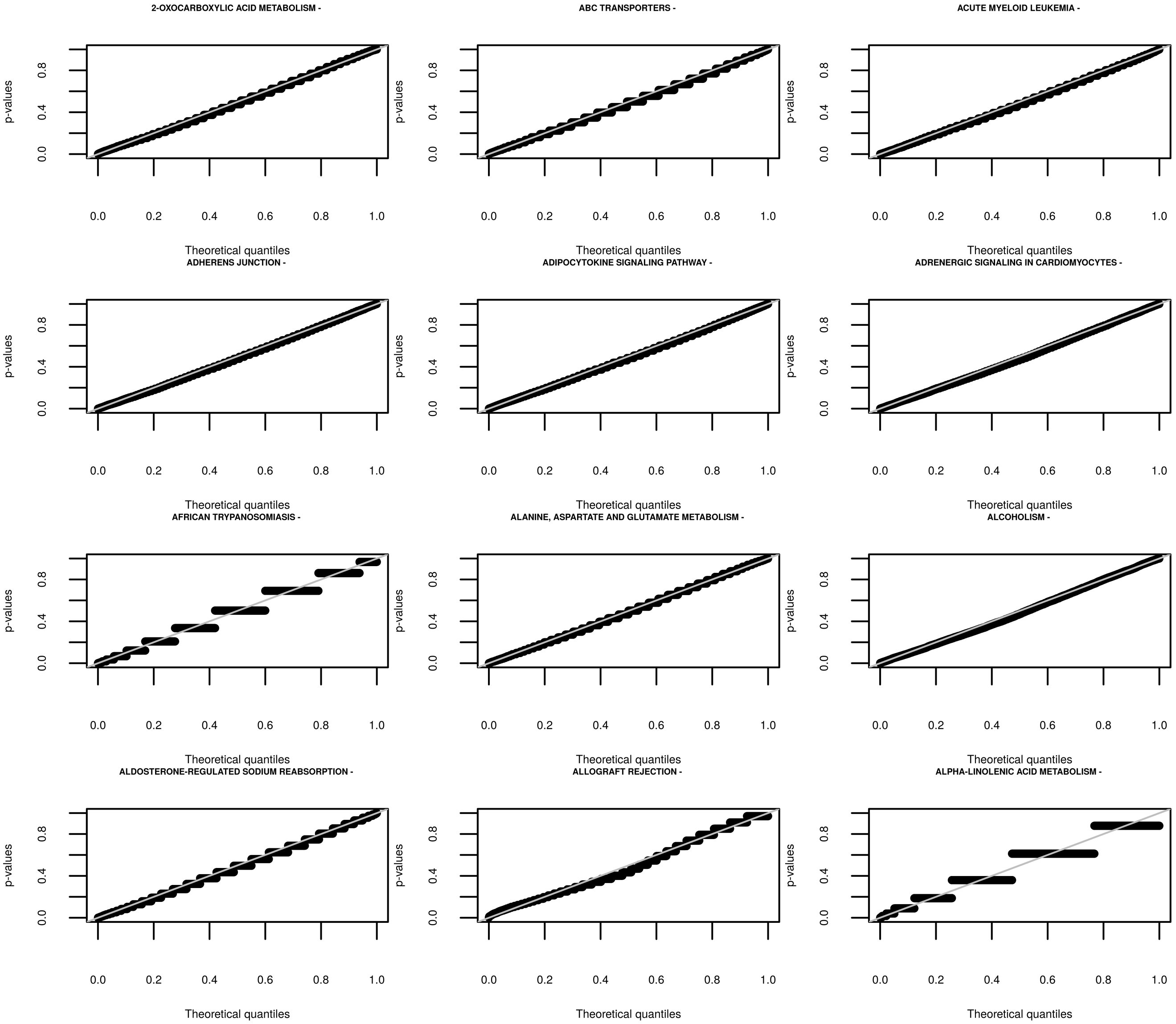

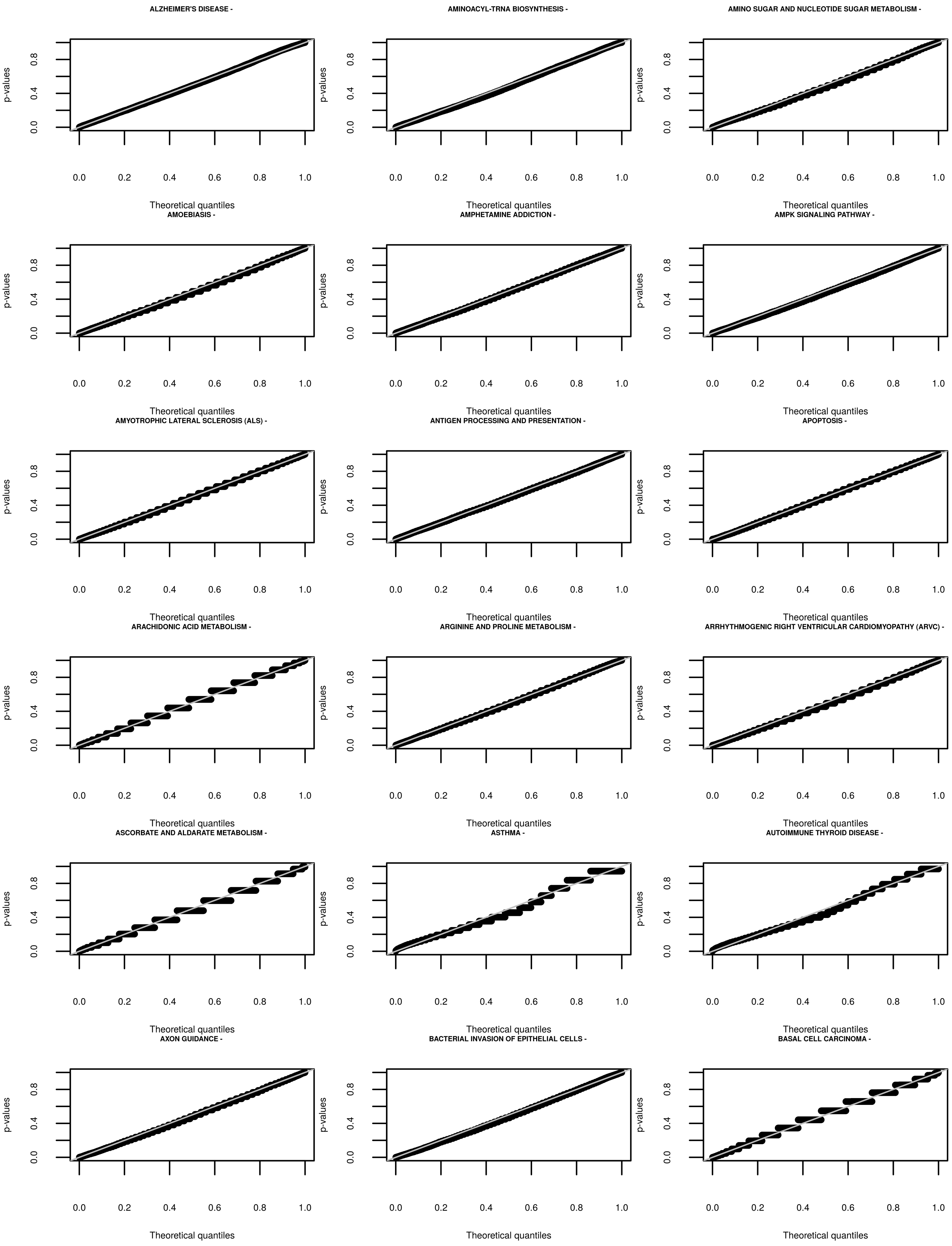
ALZHEIMER'S DISEASE -
AMINOACYL-TRNA BIOSYNTHESIS -
AMINO SUGAR AND NUCLEOTIDE SUGAR METABOLISM -
AMOEBIASIS -
AMPHETAMINE ADDICTION -
AMPK SIGNALING PATHWAY -
AMYOTROPHIC LATERAL SCLEROSIS (ALS) -
ANTIGEN PROCESSING AND PRESENTATION -
APOPTOSIS -
ARACHIDONIC ACID METABOLISM -
ARGININE AND PROLINE METABOLISM -
ARRHYTHMOGENIC RIGHT VENTRICULAR CARDIOMYOPATHY (ARVC) -
ASCORBATE AND ALDARATE METABOLISM -
ASTHMA -
AUTOIMMUNE THYROID DISEASE -
AXON GUIDANCE -
BACTERIAL INVASION OF EPITHELIAL CELLS -
BASAL CELL CARCINOMA -
p-values
0.0
0.4
0.8
0.0
0.2
0.4
0.6
0.8
1.0
Theoretical quantiles

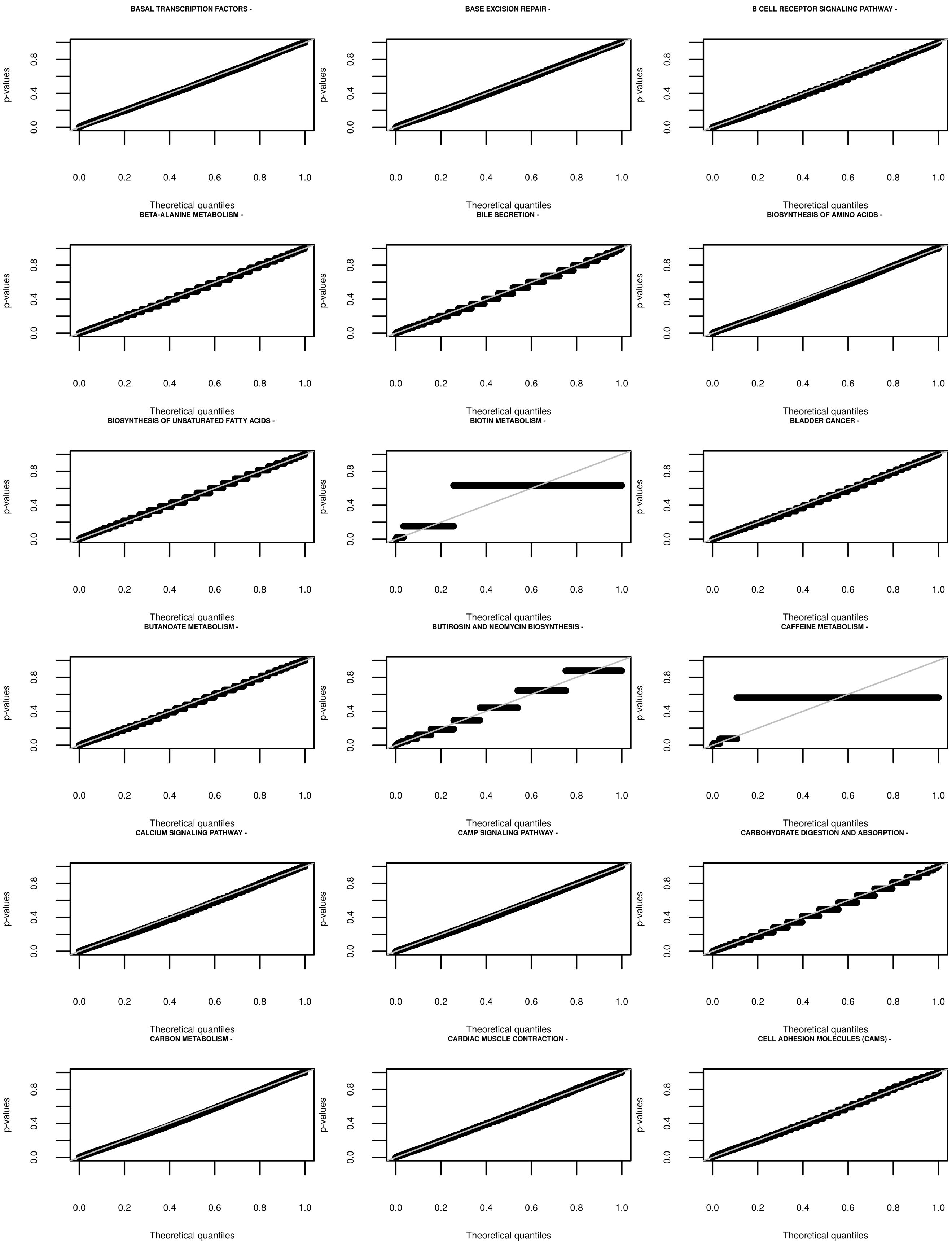

BASAL TRANSCRIPTION FACTORS -
BASE EXCISION REPAIR -
B CELL RECEPTOR SIGNALING PATHWAY -
BETA-ALANINE METABOLISM -
BILE SECRETION -
BIOSYNTHESIS OF AMINO ACIDS -
BIOSYNTHESIS OF UNSATURATED FATTY ACIDS -
BIOTIN METABOLISM -
BLADDER CANCER -
BUTANOATE METABOLISM -
BUTIROSIN AND NEOMYCIN BIOSYNTHESIS -
CAFFEINE METABOLISM -
CALCIUM SIGNALING PATHWAY -
CAMP SIGNALING PATHWAY -
CARBOHYDRATE DIGESTION AND ABSORPTION -
CARBON METABOLISM -
CARDIAC MUSCLE CONTRACTION -
CELL ADHESION MOLECULES (CAMS) -
p-values
0.0
0.4
0.8
Theoretical quantiles
0.0
0.2
0.4
0.6
0.8
1.0

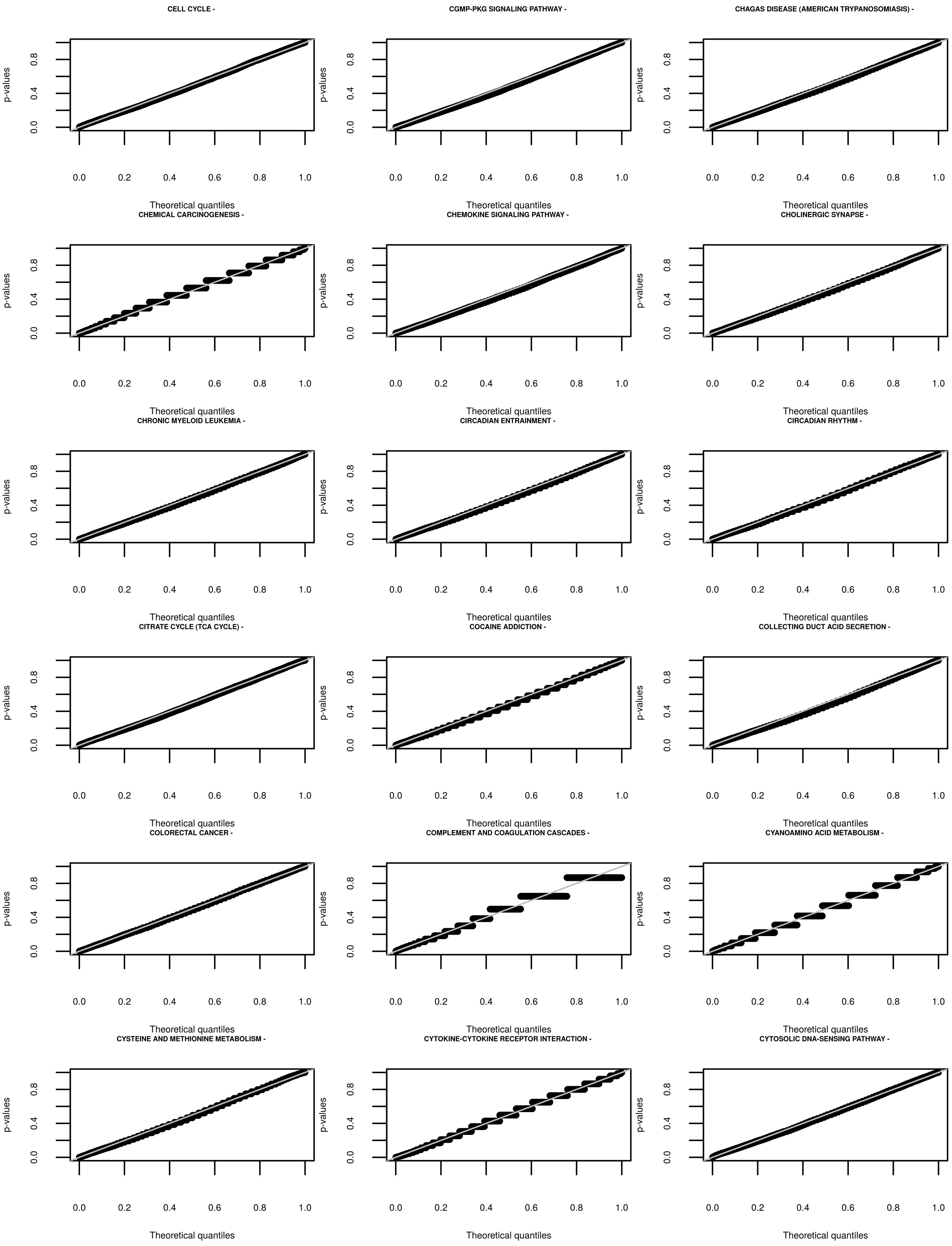
CELL CYCLE -
CGMP-PKG SIGNALING PATHWAY -
CHAGAS DISEASE (AMERICAN TRYPANOSOMIASIS) -
CHEMICAL CARCINOGENESIS -
CHEMOKINE SIGNALING PATHWAY -
CHOLINERGIC SYNAPSE -
CHRONIC MYELOID LEUKEMIA -
CIRCADIAN ENTRAINMENT -
CIRCADIAN RHYTHM -
CITRATE CYCLE (TCA CYCLE) -
COCAINE ADDICTION -
COLLECTING DUCT ACID SECRETION -
COLORECTAL CANCER -
COMPLEMENT AND COAGULATION CASCADES -
CYANOAMINO ACID METABOLISM -
CYSTEINE AND METHIONINE METABOLISM -
CYTOKINE-CYTOKINE RECEPTOR INTERACTION -
CYTOSOLIC DNA-SENSING PATHWAY -
p-values
Theoretical quantiles

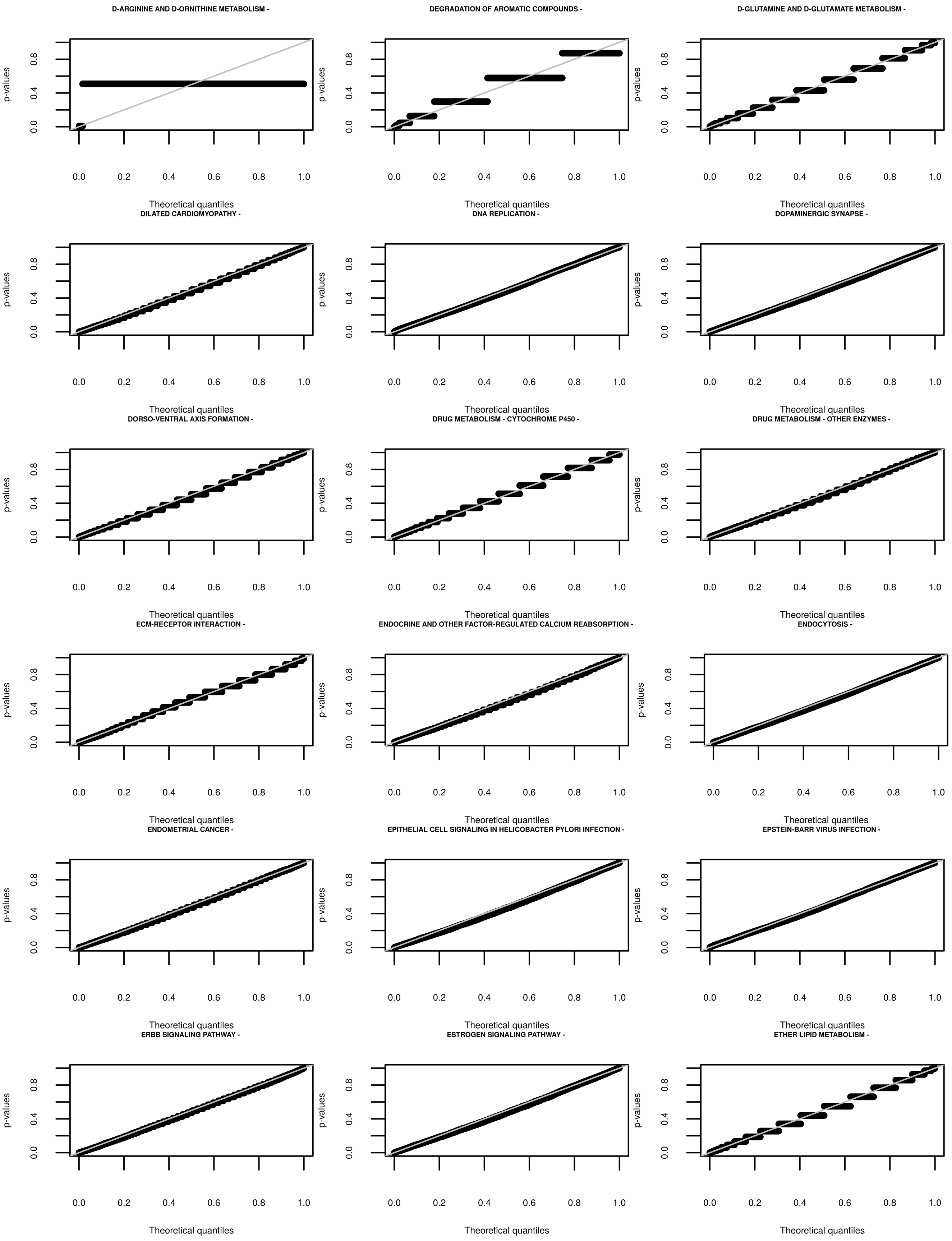

D-ARGININE AND D-ORNITHINE METABOLISM -
p-values
0.0 0.4 0.8
0.0 0.2 0.4 0.6 0.8 1.0
Theoretical quantiles
DEGRADATION OF AROMATIC COMPOUNDS -
p-values
0.0 0.4 0.8
0.0 0.2 0.4 0.6 0.8 1.0
Theoretical quantiles
D-GLUTAMINE AND D-GLUTAMATE METABOLISM -
p-values
0.0 0.4 0.8
0.0 0.2 0.4 0.6 0.8 1.0
Theoretical quantiles
DILATED CARDIOMYOPATHY -
p-values
0.0 0.4 0.8
0.0 0.2 0.4 0.6 0.8 1.0
Theoretical quantiles
DNA REPLICATION -
p-values
0.0 0.4 0.8
0.0 0.2 0.4 0.6 0.8 1.0
Theoretical quantiles
DOPAMINERGIC SYNAPSE -
p-values
0.0 0.4 0.8
0.0 0.2 0.4 0.6 0.8 1.0
Theoretical quantiles
DORSO-VENTRAL AXIS FORMATION -
p-values
0.0 0.4 0.8
0.0 0.2 0.4 0.6 0.8 1.0
Theoretical quantiles
DRUG METABOLISM - CYTOCHROME P450 -
p-values
0.0 0.4 0.8
0.0 0.2 0.4 0.6 0.8 1.0
Theoretical quantiles
DRUG METABOLISM - OTHER ENZYMES -
p-values
0.0 0.4 0.8
0.0 0.2 0.4 0.6 0.8 1.0
Theoretical quantiles
ECM-RECEPTOR INTERACTION -
p-values
0.0 0.4 0.8
0.0 0.2 0.4 0.6 0.8 1.0
Theoretical quantiles
ENDOCRINE AND OTHER FACTOR-REGULATED CALCIUM REABSORPTION -
p-values
0.0 0.4 0.8
0.0 0.2 0.4 0.6 0.8 1.0
Theoretical quantiles
ENDOCYTOSIS -
p-values
0.0 0.4 0.8
0.0 0.2 0.4 0.6 0.8 1.0
Theoretical quantiles
ENDOMETRIAL CANCER -
p-values
0.0 0.4 0.8
0.0 0.2 0.4 0.6 0.8 1.0
Theoretical quantiles
EPITHELIAL CELL SIGNALING IN HELICOBACTER PYLORI INFECTION -
p-values
0.0 0.4 0.8
0.0 0.2 0.4 0.6 0.8 1.0
Theoretical quantiles
EPSTEIN-BARR VIRUS INFECTION -
p-values
0.0 0.4 0.8
0.0 0.2 0.4 0.6 0.8 1.0
Theoretical quantiles
ERBB SIGNALING PATHWAY -
p-values
0.0 0.4 0.8
0.0 0.2 0.4 0.6 0.8 1.0
Theoretical quantiles
ESTROGEN SIGNALING PATHWAY -
p-values
0.0 0.4 0.8
0.0 0.2 0.4 0.6 0.8 1.0
Theoretical quantiles
ETHER LIPID METABOLISM -
p-values
0.0 0.4 0.8
0.0 0.2 0.4 0.6 0.8 1.0
Theoretical quantiles

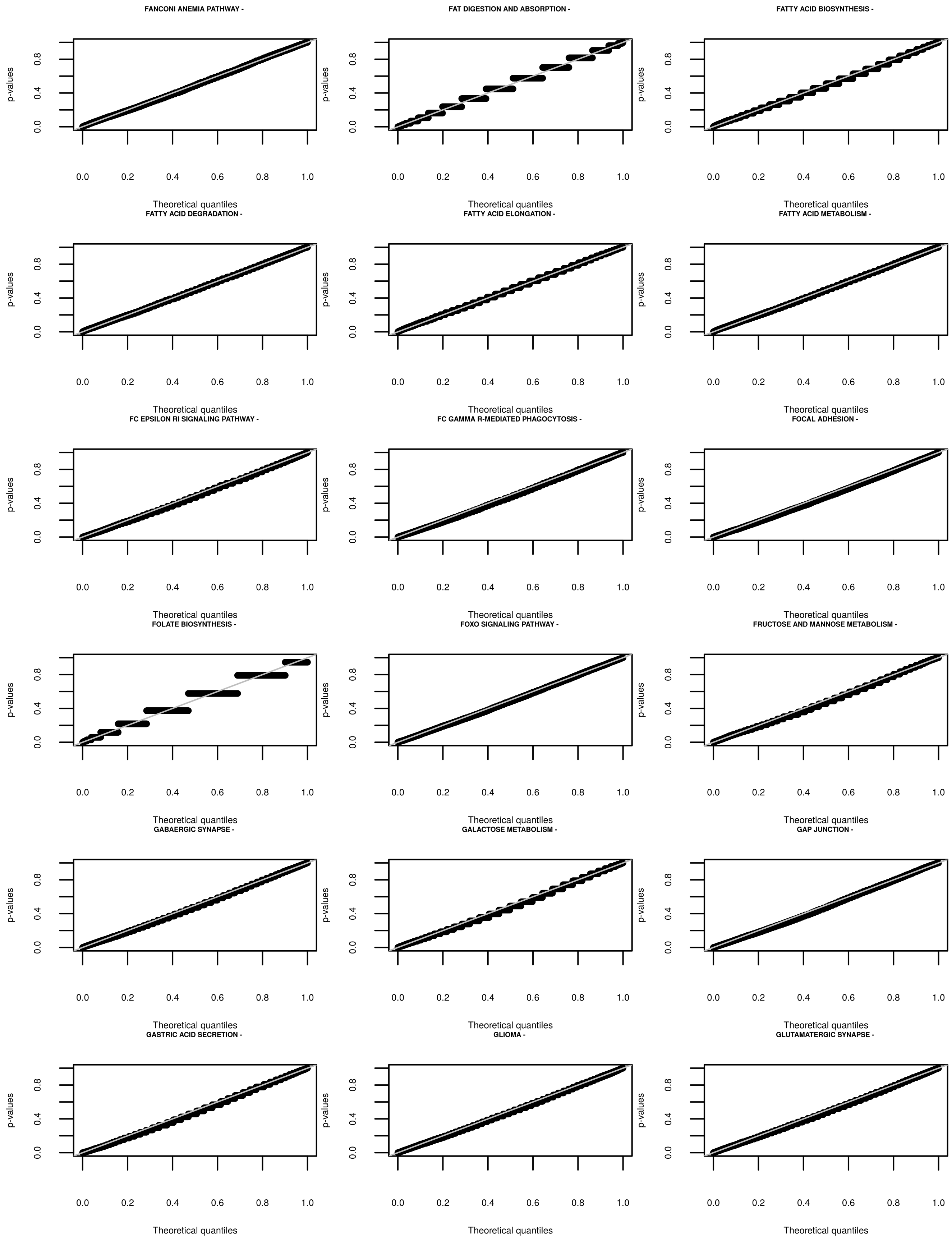
FANCONI ANEMIA PATHWAY -
FAT DIGESTION AND ABSORPTION -
FATTY ACID BIOSYNTHESIS -
FATTY ACID DEGRADATION -
FATTY ACID ELONGATION -
FATTY ACID METABOLISM -
FC EPSILON RI SIGNALING PATHWAY -
FC GAMMA R-MEDIATED PHAGOCYTOSIS -
FOCAL ADHESION -
FOLATE BIOSYNTHESIS -
FOXO SIGNALING PATHWAY -
FRUCTOSE AND MANNOSE METABOLISM -
GABAERGIC SYNAPSE -
GALACTOSE METABOLISM -
GAP JUNCTION -
GASTRIC ACID SECRETION -
GLIOMA -
GLUTAMATERGIC SYNAPSE -
p-values
0.0
0.4
0.8
Theoretical quantiles
0.0
0.2
0.4
0.6
0.8
1.0

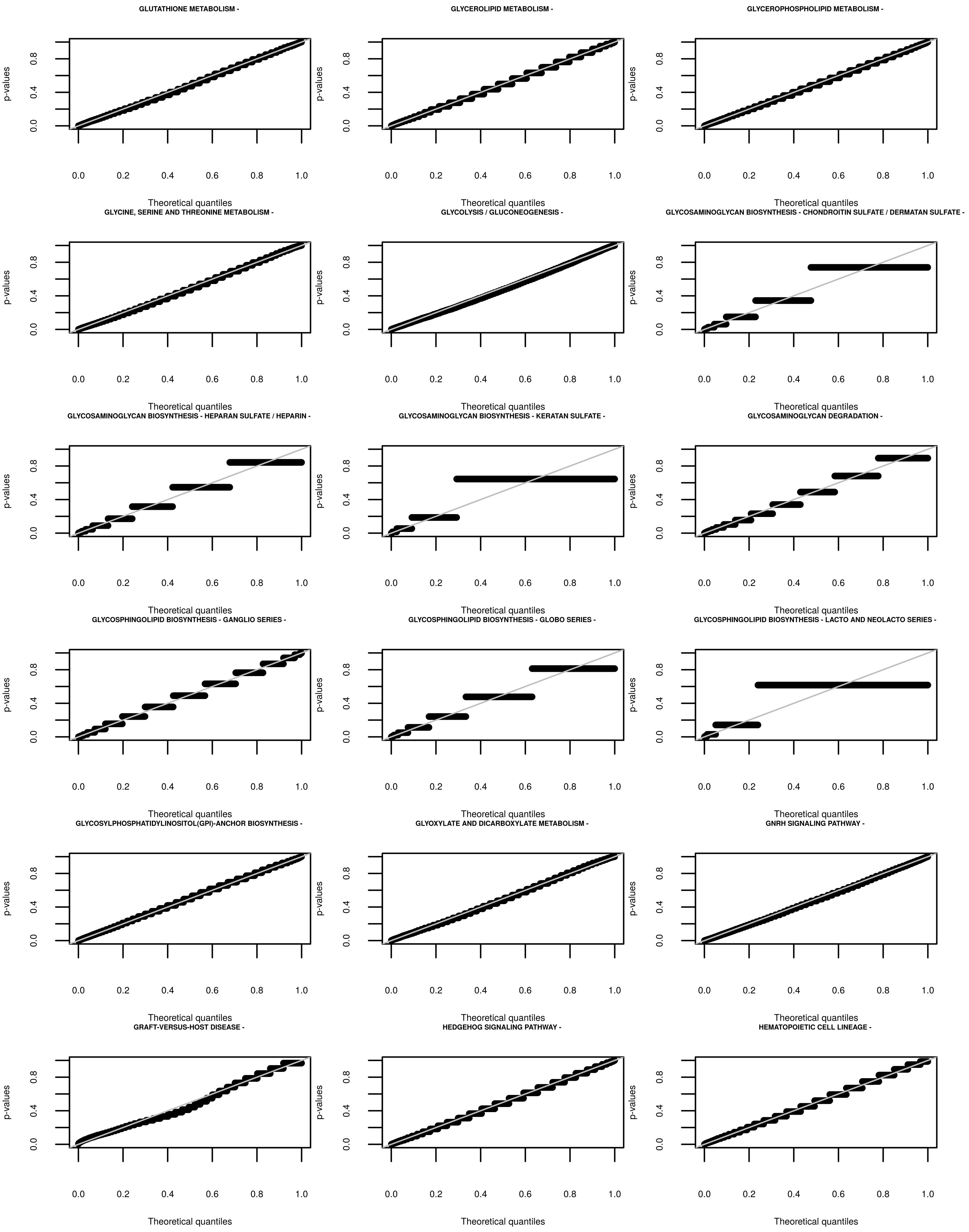
GLUTATHIONE METABOLISM -
GLYCEROLIPID METABOLISM -
GLYCEROPHOSPHOLIPID METABOLISM -
GLYCINE, SERINE AND THREONINE METABOLISM -
GLYCOLYSIS / GLUCONEOGENESIS -
GLYCOSAMINOGLYCAN BIOSYNTHESIS - CHONDROITIN SULFATE / DERMATAN SULFATE -
GLYCOSAMINOGLYCAN BIOSYNTHESIS - HEPARAN SULFATE / HEPARIN -
GLYCOSAMINOGLYCAN BIOSYNTHESIS - KERATAN SULFATE -
GLYCOSAMINOGLYCAN DEGRADATION -
GLYCOSPHINGOLIPID BIOSYNTHESIS - GANGLIO SERIES -
GLYCOSPHINGOLIPID BIOSYNTHESIS - GLOBO SERIES -
GLYCOSPHINGOLIPID BIOSYNTHESIS - LACTO AND NEOLACTO SERIES -
GLYCOSYLPHOSPHATIDYLINOSITOL(GPI)-ANCHOR BIOSYNTHESIS -
GLYOXYLATE AND DICARBOXYLATE METABOLISM -
GNRH SIGNALING PATHWAY -
GRAFT-VERSUS-HOST DISEASE -
HEDGEHOG SIGNALING PATHWAY -
HEMATOPOIETIC CELL LINEAGE -
p-values
0.0
0.4
0.8
0.0
0.2
0.4
0.6
0.8
1.0
Theoretical quantiles

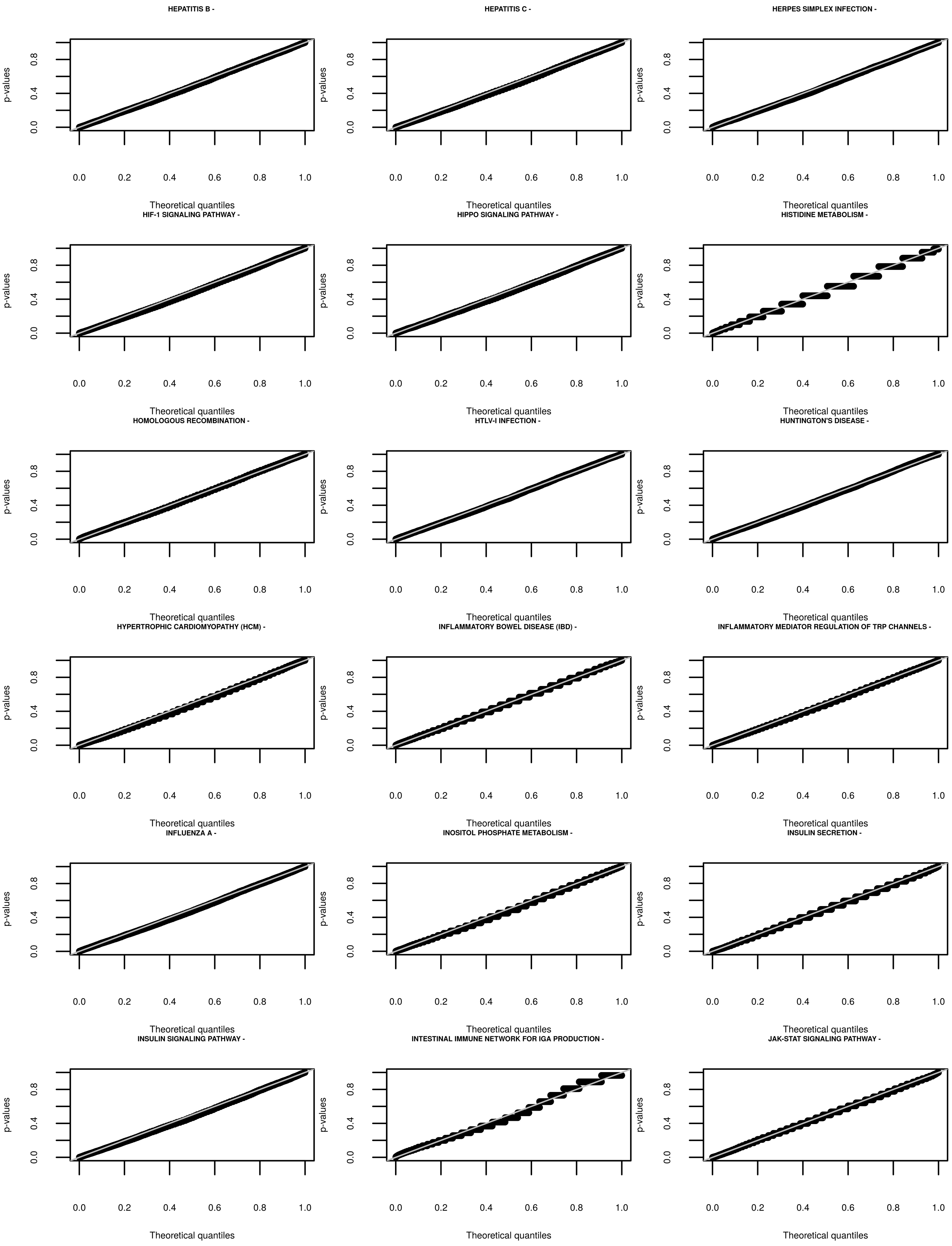
HEPATITIS B -
HEPATITIS C -
HERPES SIMPLEX INFECTION -
HIF-1 SIGNALING PATHWAY -
HIPPO SIGNALING PATHWAY -
HISTIDINE METABOLISM -
HOMOLOGOUS RECOMBINATION -
HTLV-I INFECTION -
HUNTINGTON'S DISEASE -
HYPERTROPHIC CARDIOMYOPATHY (HCM) -
INFLAMMATORY BOWEL DISEASE (IBD) -
INFLAMMATORY MEDIATOR REGULATION OF TRP CHANNELS -
INFLUENZA A -
INOSITOL PHOSPHATE METABOLISM -
INSULIN SECRETION -
INSULIN SIGNALING PATHWAY -
INTESTINAL IMMUNE NETWORK FOR IGA PRODUCTION -
JAK-STAT SIGNALING PATHWAY -
p-values
0.0
0.4
0.8
Theoretical quantiles
0.0
0.2
0.4
0.6
0.8
1.0

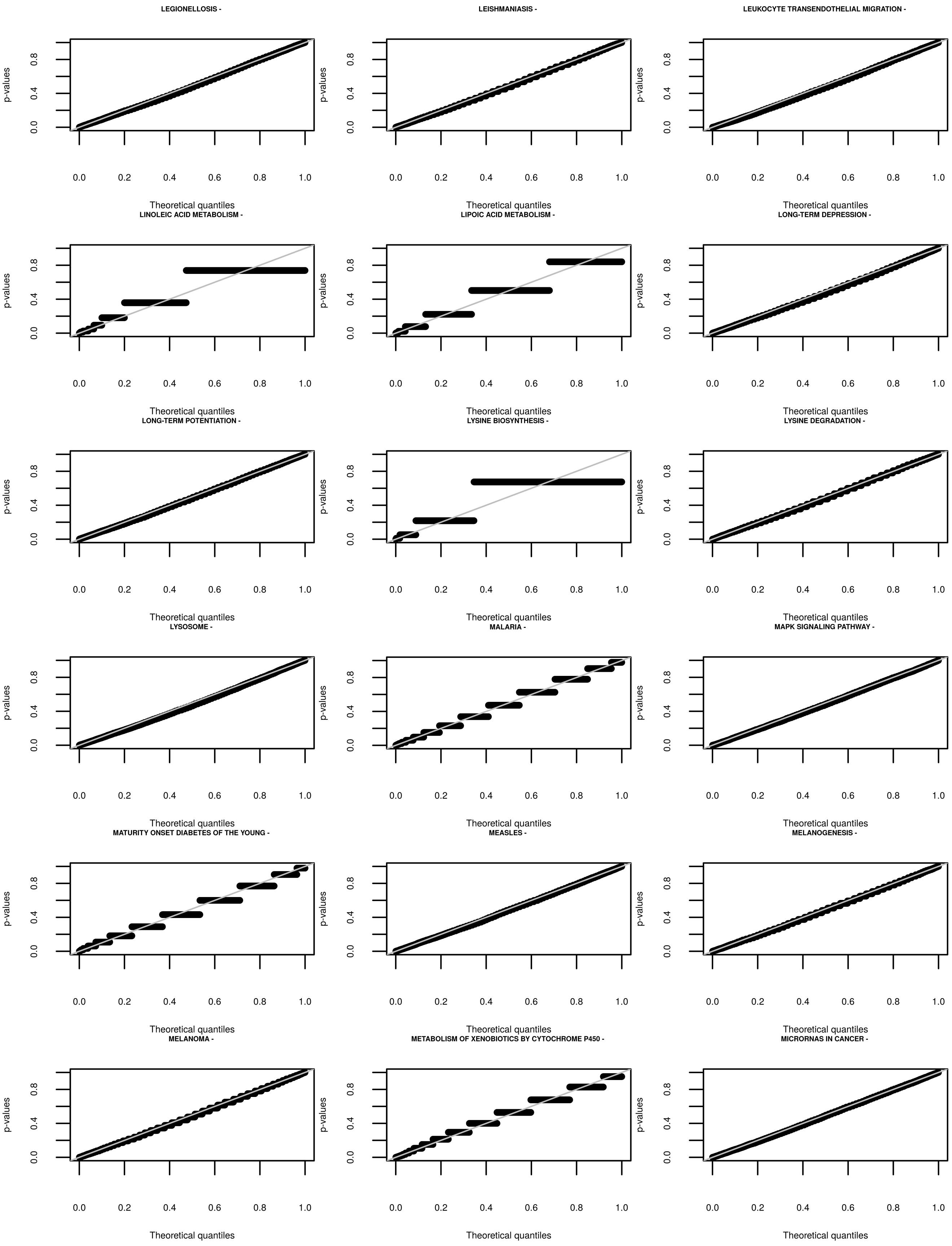

LEGIONELLOSIS -
LEISHMANIASIS -
LEUKOCYTE TRANSENDOTHELIAL MIGRATION -
LINOLEIC ACID METABOLISM -
LIPOIC ACID METABOLISM -
LONG-TERM DEPRESSION -
LONG-TERM POTENTIATION -
LYSINE BIOSYNTHESIS -
LYSINE DEGRADATION -
LYSOSOME -
MALARIA -
MAPK SIGNALING PATHWAY -
MATURITY ONSET DIABETES OF THE YOUNG -
MEASLES -
MELANOGENESIS -
MELANOMA -
METABOLISM OF XENOBIOTICS BY CYTOCHROME P450 -
MICRORNAS IN CANCER -
p-values
0.0
0.4
0.8
Theoretical quantiles
0.0
0.2
0.4
0.6
0.8
1.0

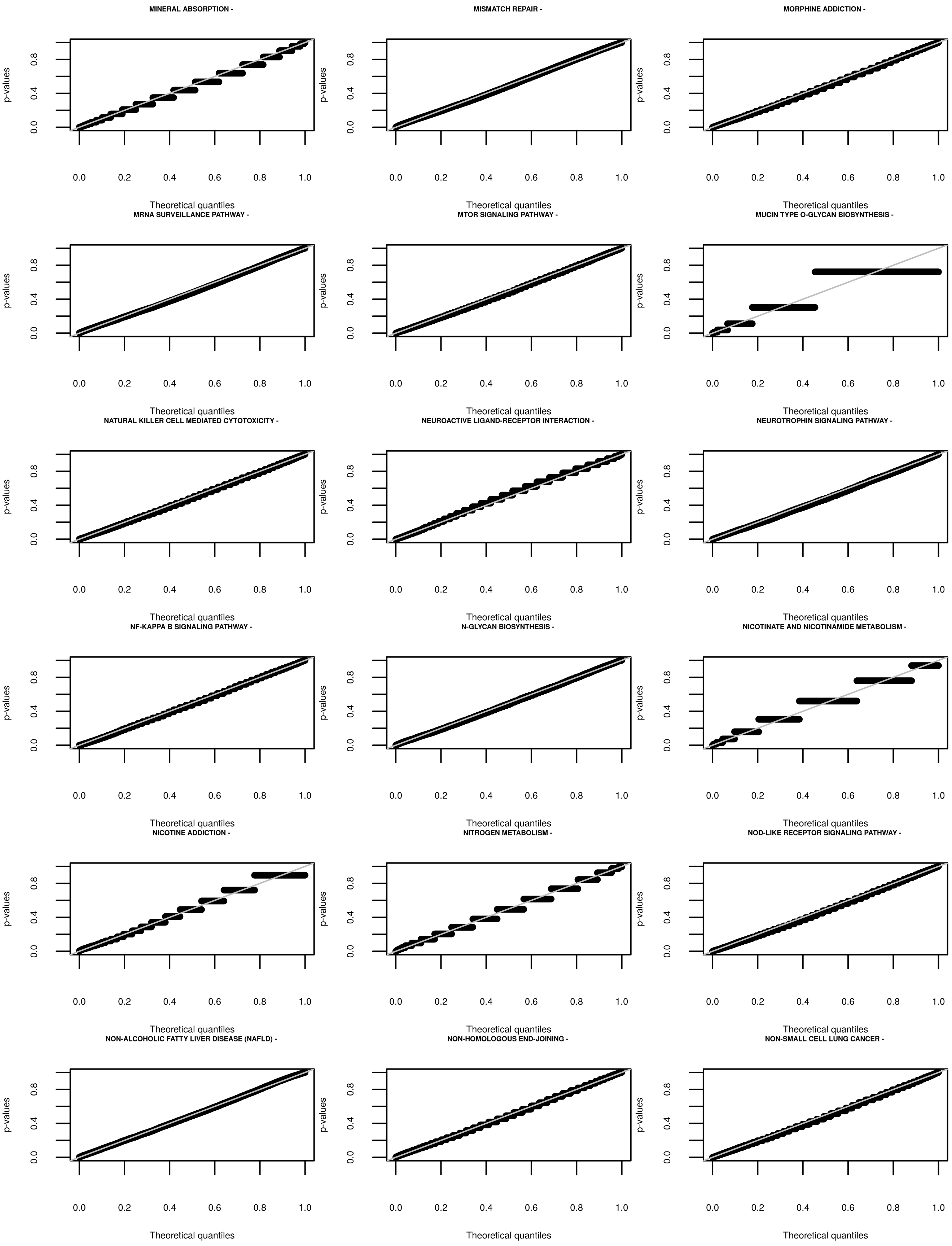
MINERAL ABSORPTION -
MISMATCH REPAIR -
MORPHINE ADDICTION -
MRNA SURVEILLANCE PATHWAY -
MTOR SIGNALING PATHWAY -
MUCIN TYPE O-GLYCAN BIOSYNTHESIS -
NATURAL KILLER CELL MEDIATED CYTOTOXICITY -
NEUROACTIVE LIGAND-RECEPTOR INTERACTION -
NEUROTROPHIN SIGNALING PATHWAY -
NF-KAPPA B SIGNALING PATHWAY -
N-GLYCAN BIOSYNTHESIS -
NICOTINATE AND NICOTINAMIDE METABOLISM -
NICOTINE ADDICTION -
NITROGEN METABOLISM -
NOD-LIKE RECEPTOR SIGNALING PATHWAY -
NON-ALCOHOLIC FATTY LIVER DISEASE (NAFLD) -
NON-HOMOLOGOUS END-JOINING -
NON-SMALL CELL LUNG CANCER -
p-values
0.0
0.4
0.8
Theoretical quantiles
0.0
0.2
0.4
0.6
0.8
1.0

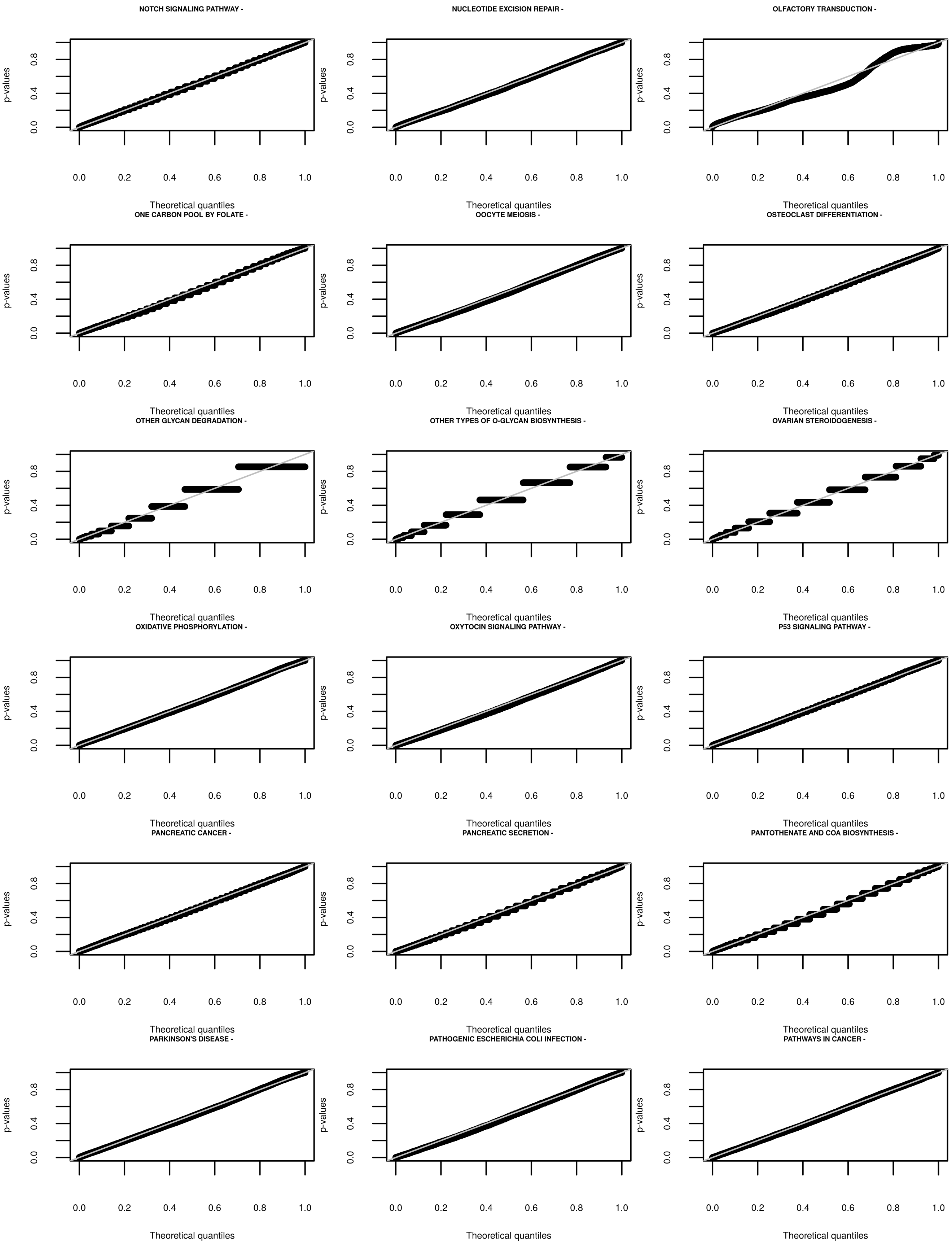
NOTCH SIGNALING PATHWAY -
NUCLEOTIDE EXCISION REPAIR -
OLFACTORY TRANSDUCTION -
ONE CARBON POOL BY FOLATE -
OOCYTE MEIOSIS -
OSTEOCLAST DIFFERENTIATION -
OTHER GLYCAN DEGRADATION -
OTHER TYPES OF O-GLYCAN BIOSYNTHESIS -
OVARIAN STEROIDOGENESIS -
OXIDATIVE PHOSPHORYLATION -
OXYTOCIN SIGNALING PATHWAY -
P53 SIGNALING PATHWAY -
PANCREATIC CANCER -
PANCREATIC SECRETION -
PANTOTHENATE AND COA BIOSYNTHESIS -
PARKINSON'S DISEASE -
PATHOGENIC ESCHERICHIA COLI INFECTION -
PATHWAYS IN CANCER -
p-values
0.0
0.4
0.8
Theoretical quantiles
0.0
0.2
0.4
0.6
0.8
1.0

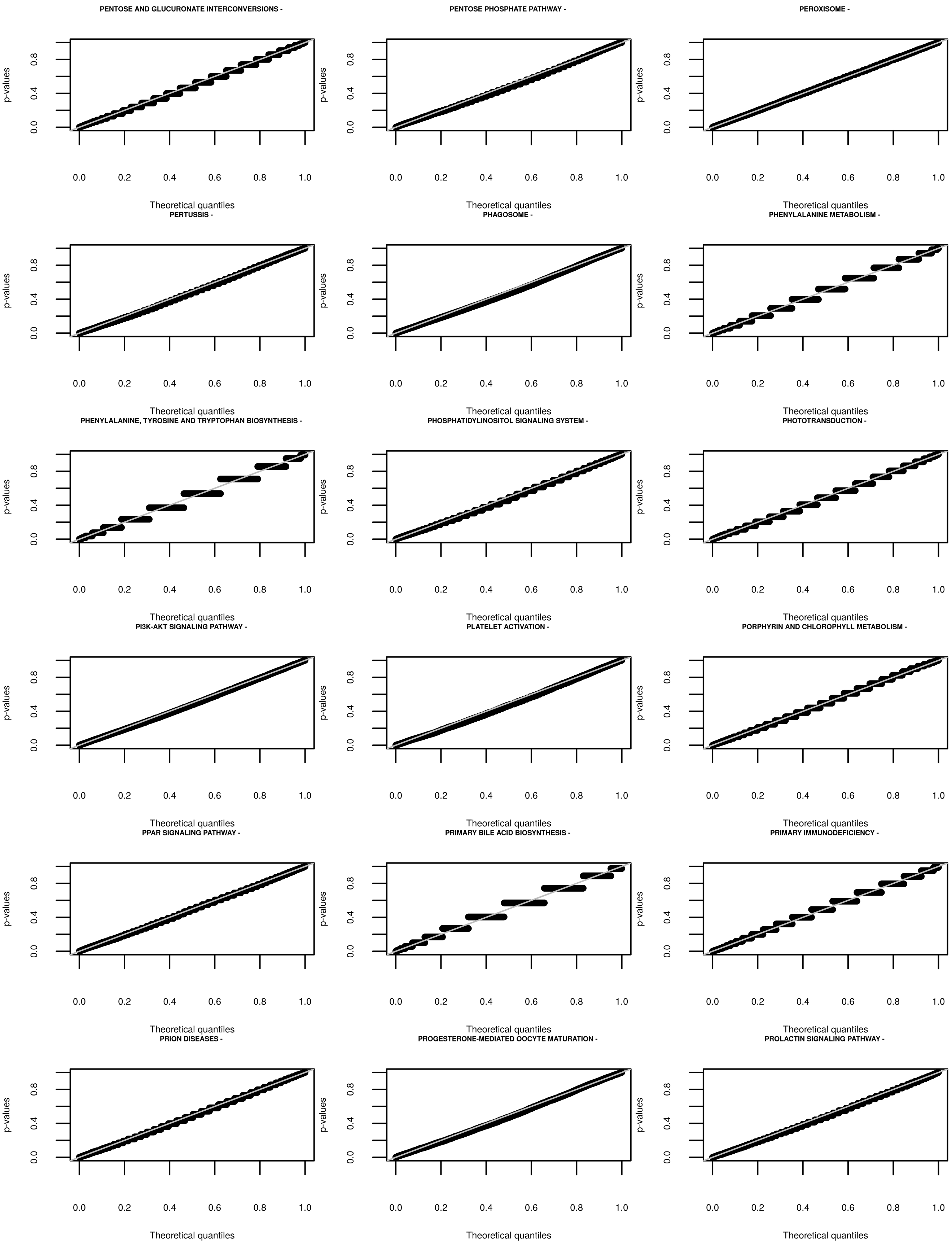

PENTOSE AND GLUCURONATE INTERCONVERSIONS -
PENTOSE PHOSPHATE PATHWAY -
PEROXISOME -
PERTUSSIS -
PHAGOSOME -
PHENYLALANINE METABOLISM -
PHENYLALANINE, TYROSINE AND TRYPTOPHAN BIOSYNTHESIS -
PHOSPHATIDYLINOSITOL SIGNALING SYSTEM -
PHOTOTRANSDUCTION -
PI3K-AKT SIGNALING PATHWAY -
PLATELET ACTIVATION -
PORPHYRIN AND CHLOROPHYLL METABOLISM -
PPAR SIGNALING PATHWAY -
PRIMARY BILE ACID BIOSYNTHESIS -
PRIMARY IMMUNODEFICIENCY -
PRION DISEASES -
PROGESTERONE-MEDIATED OOCYTE MATURATION -
PROLACTIN SIGNALING PATHWAY -
p-values
0.0
0.4
0.8
Theoretical quantiles
0.0
0.2
0.4
0.6
0.8
1.0

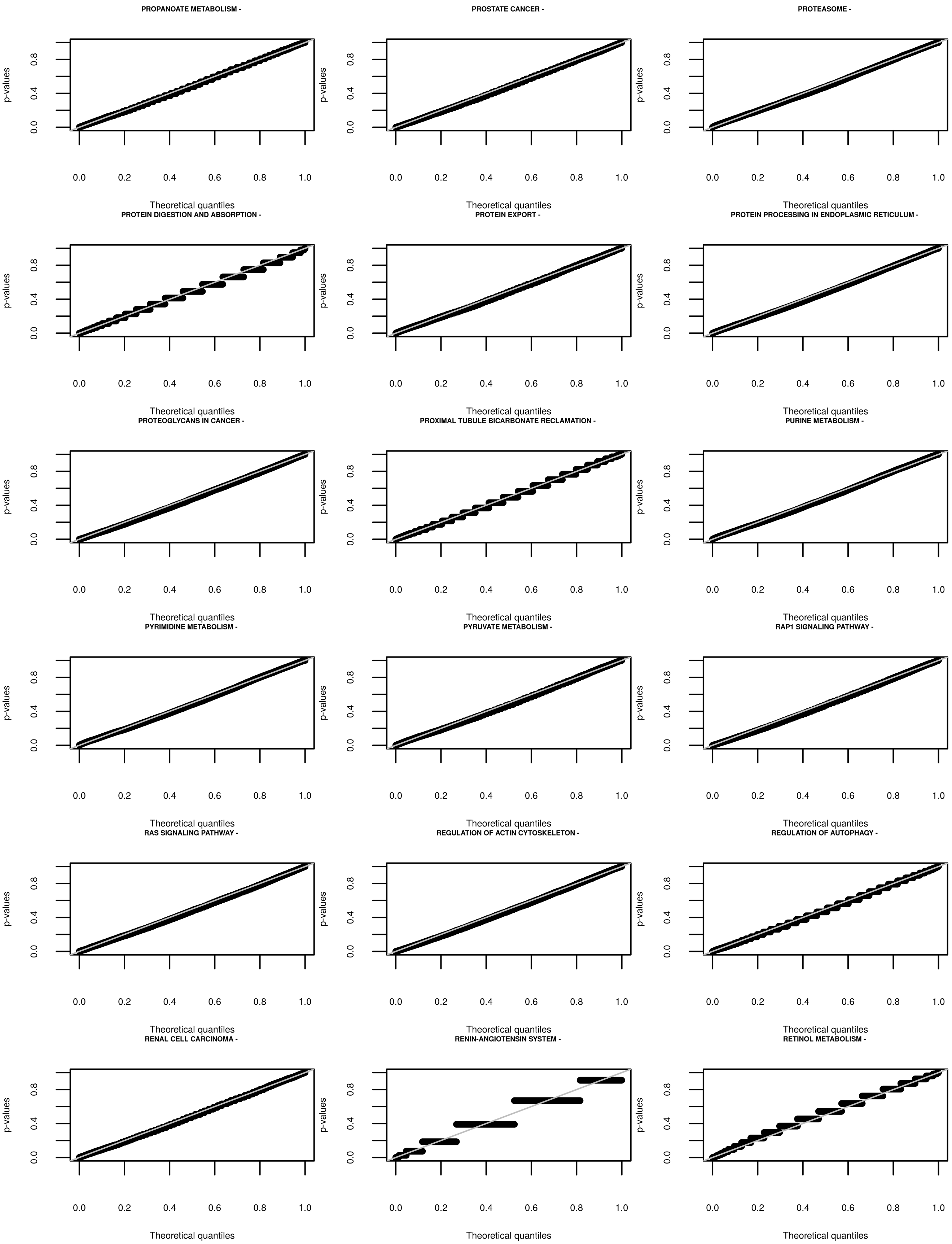
PROPANOATE METABOLISM -
PROSTATE CANCER -
PROTEASOME -
PROTEIN DIGESTION AND ABSORPTION -
PROTEIN EXPORT -
PROTEIN PROCESSING IN ENDOPLASMIC RETICULUM -
PROTEOGLYCANS IN CANCER -
PROXIMAL TUBULE BICARBONATE RECLAMATION -
PURINE METABOLISM -
PYRIMIDINE METABOLISM -
PYRUVATE METABOLISM -
RAP1 SIGNALING PATHWAY -
RAS SIGNALING PATHWAY -
REGULATION OF ACTIN CYTOSKELETON -
REGULATION OF AUTOPHAGY -
RENAL CELL CARCINOMA -
RENIN-ANGIOTENSIN SYSTEM -
RETINOL METABOLISM -
p-values
0.0
0.4
0.8
0.0
0.2
0.4
0.6
0.8
1.0
Theoretical quantiles

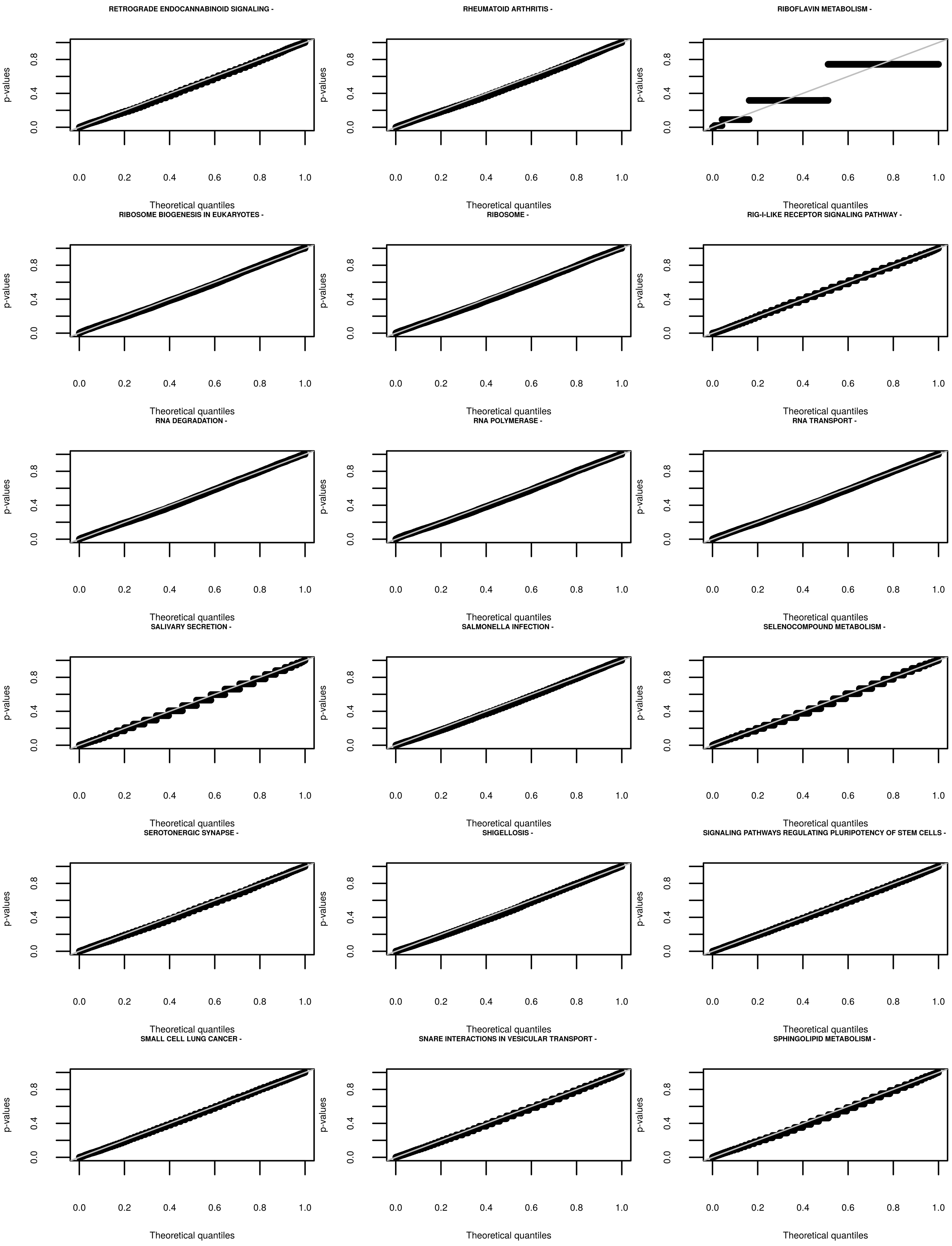
RETROGRADE ENDOCANNABINOID SIGNALING -
RHEUMATOID ARTHRITIS -
RIBOFLAVIN METABOLISM -
RIBOSOME BIOGENESIS IN EUKARYOTES -
RIBOSOME -
RIG-I-LIKE RECEPTOR SIGNALING PATHWAY -
RNA DEGRADATION -
RNA POLYMERASE -
RNA TRANSPORT -
SALIVARY SECRETION -
SALMONELLA INFECTION -
SELENOCOMPOUND METABOLISM -
SEROTONERGIC SYNAPSE -
SHIGELLOSIS -
SIGNALING PATHWAYS REGULATING PLURIPOTENCY OF STEM CELLS -
SMALL CELL LUNG CANCER -
SNARE INTERACTIONS IN VESICULAR TRANSPORT -
SPHINGOLIPID METABOLISM -
p-values
0.0
0.4
0.8
Theoretical quantiles
0.0
0.2
0.4
0.6
0.8
1.0

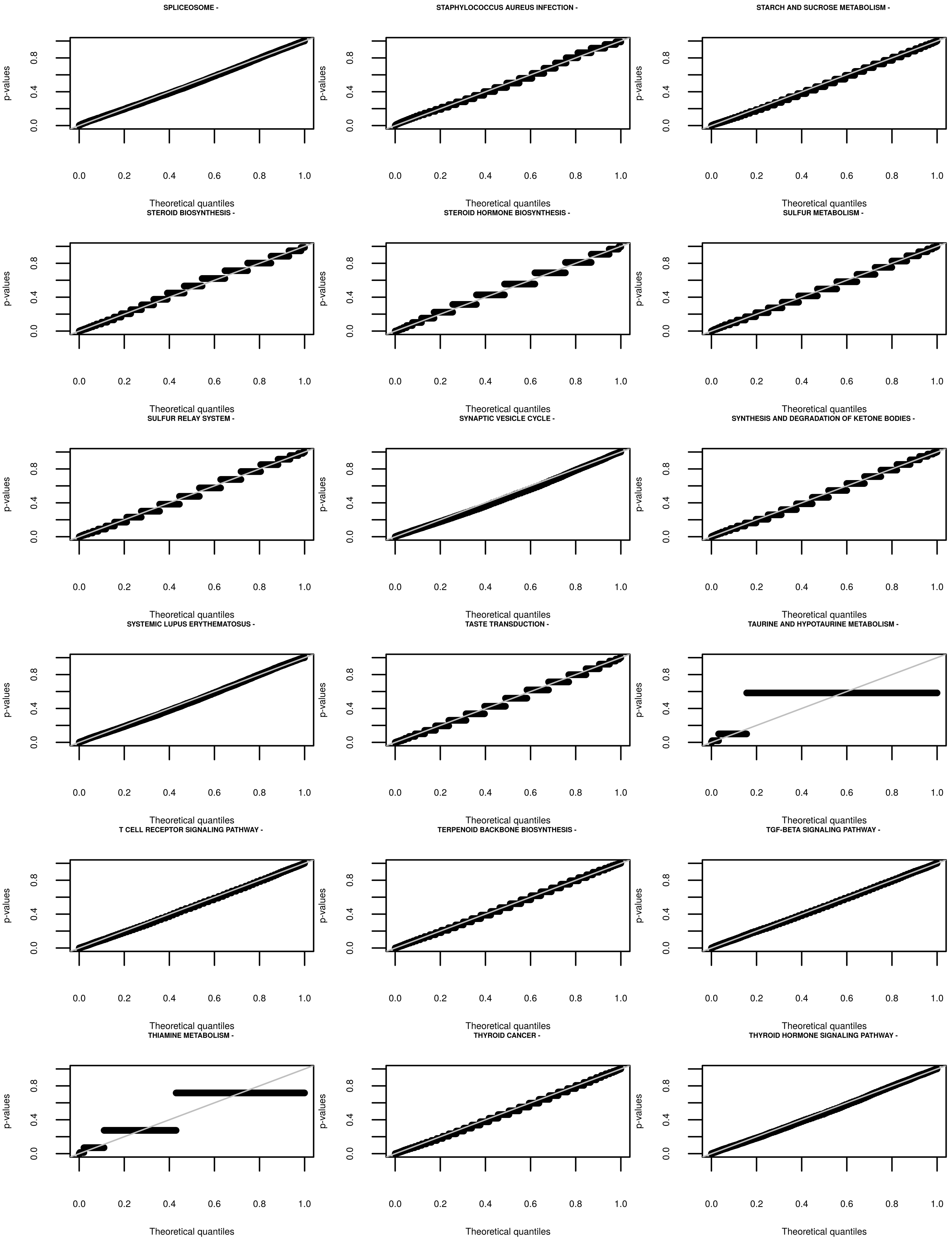
SPLICEOSOME -
STAPHYLOCOCCUS AUREUS INFECTION -
STARCH AND SUCROSE METABOLISM -
STEROID BIOSYNTHESIS -
STEROID HORMONE BIOSYNTHESIS -
SULFUR METABOLISM -
SULFUR RELAY SYSTEM -
SYNAPTIC VESICLE CYCLE -
SYNTHESIS AND DEGRADATION OF KETONE BODIES -
SYSTEMIC LUPUS ERYTHEMATOSUS -
TASTE TRANSDUCTION -
TAURINE AND HYPOTAURINE METABOLISM -
T CELL RECEPTOR SIGNALING PATHWAY -
TERPENOID BACKBONE BIOSYNTHESIS -
TGF-BETA SIGNALING PATHWAY -
THIAMINE METABOLISM -
THYROID CANCER -
THYROID HORMONE SIGNALING PATHWAY -
p-values
0.0
0.4
0.8
Theoretical quantiles
0.0
0.2
0.4
0.6
0.8
1.0

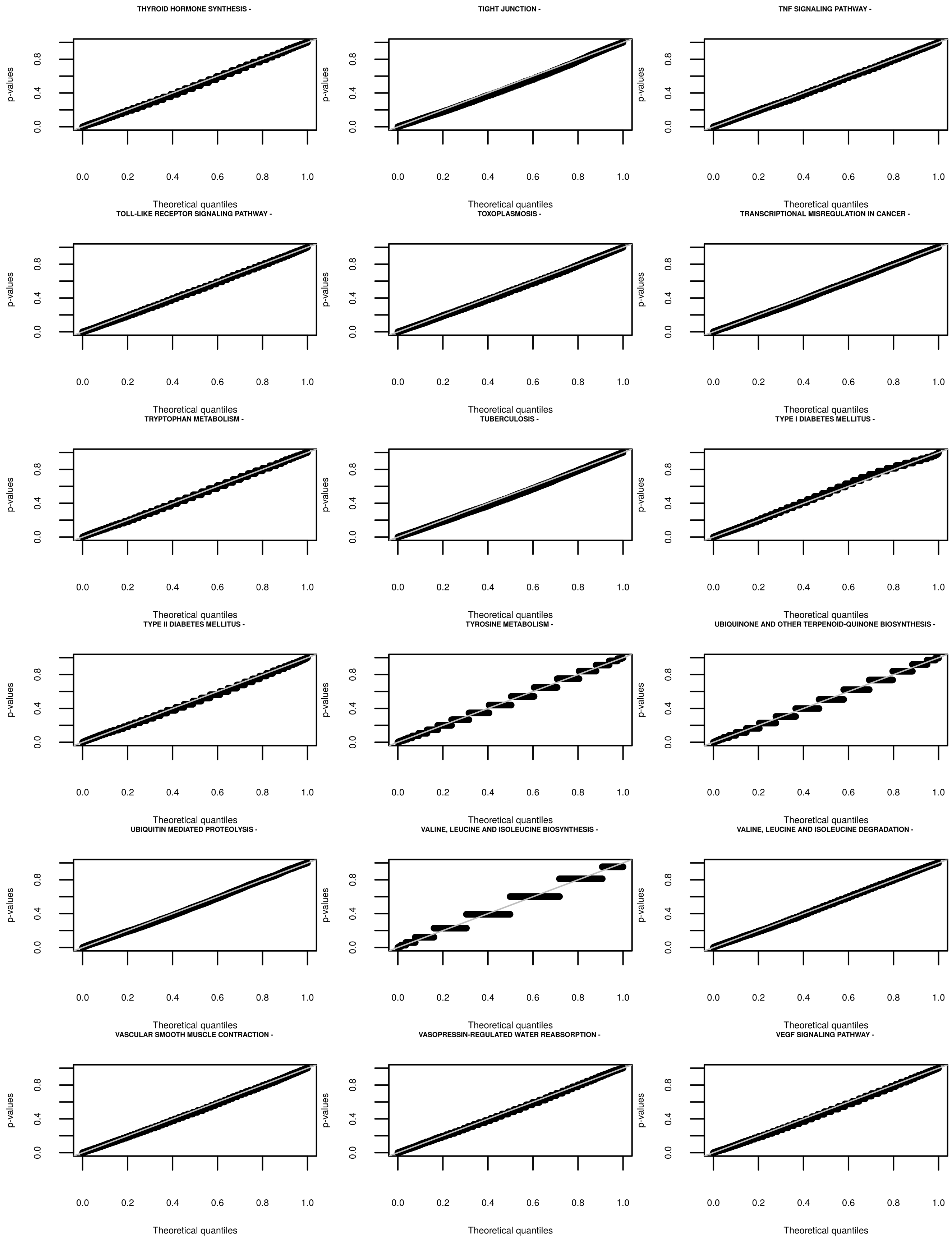
THYROID HORMONE SYNTHESIS -
TIGHT JUNCTION -
TNF SIGNALING PATHWAY -
TOLL-LIKE RECEPTOR SIGNALING PATHWAY -
TOXOPLASMOSIS -
TRANSCRIPTIONAL MISREGULATION IN CANCER -
TRYPTOPHAN METABOLISM -
TUBERCULOSIS -
TYPE I DIABETES MELLITUS -
TYPE II DIABETES MELLITUS -
TYROSINE METABOLISM -
UBIQUINONE AND OTHER TERPENOID-QUINONE BIOSYNTHESIS -
UBIQUITIN MEDIATED PROTEOLYSIS -
VALINE, LEUCINE AND ISOLEUCINE BIOSYNTHESIS -
VALINE, LEUCINE AND ISOLEUCINE DEGRADATION -
VASCULAR SMOOTH MUSCLE CONTRACTION -
VASOPRESSIN-REGULATED WATER REABSORPTION -
VEGF SIGNALING PATHWAY -
p-values
0.0
0.4
0.8
Theoretical quantiles
0.0
0.2
0.4
0.6
0.8
1.0

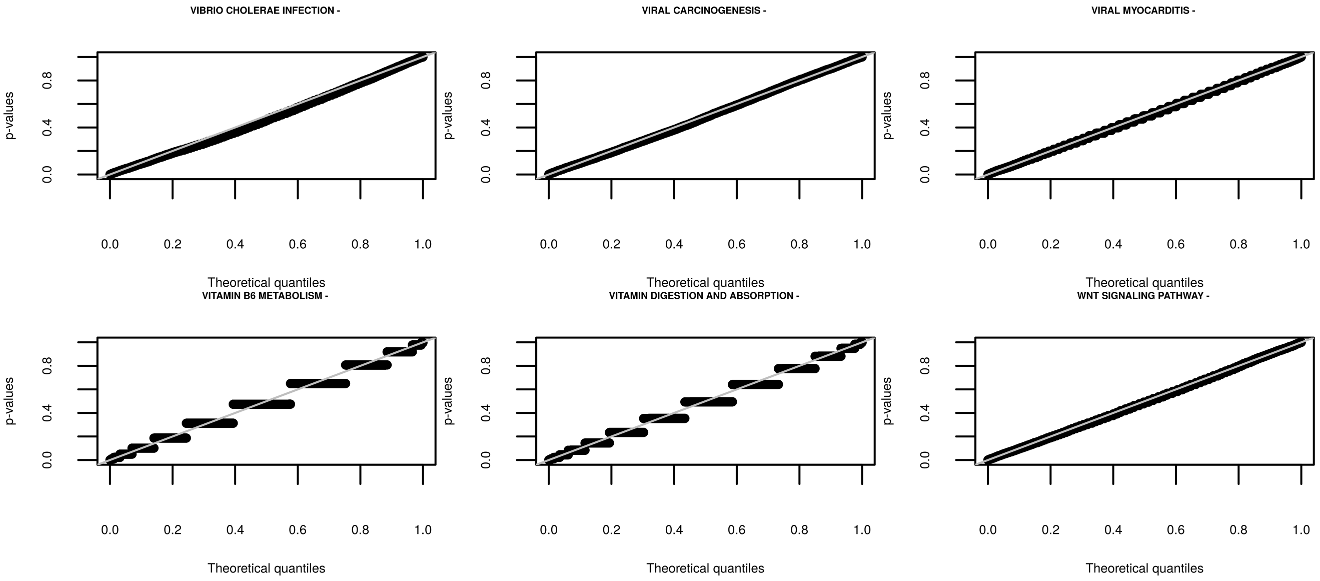
VIBRIO CHOLERAE INFECTION -
VIRAL CARCINOGENESIS -
VIRAL MYOCARDITIS -
VITAMIN B6 METABOLISM -
VITAMIN DIGESTION AND ABSORPTION -
WNT SIGNALING PATHWAY -
p-values
0.0
0.4
0.8
Theoretical quantiles
0.0
0.2
0.4
0.6
0.8
1.0

Pathway-specific model estimation for improved pathway annotation by network crosstalk

*Miguel Castresana-Aguirre, Erik L.L. Sonnhammer**

Department of Biochemistry and Biophysics, Stockholm University, Science for Life Laboratory, Box 1031, 17121 Solna, Sweden,
*To whom correspondence should be addressed: erik.sonnhammer@dbb.su.se

Supplementary Figure 2: 10000 random gene sets of size 50 are run against KEGG pathways. P-value uniformity test of ANUBIX. Reported p-values are plotted against theoretical quantile (rank). Software: R version 3.4.3 https://www.r-project.org/.

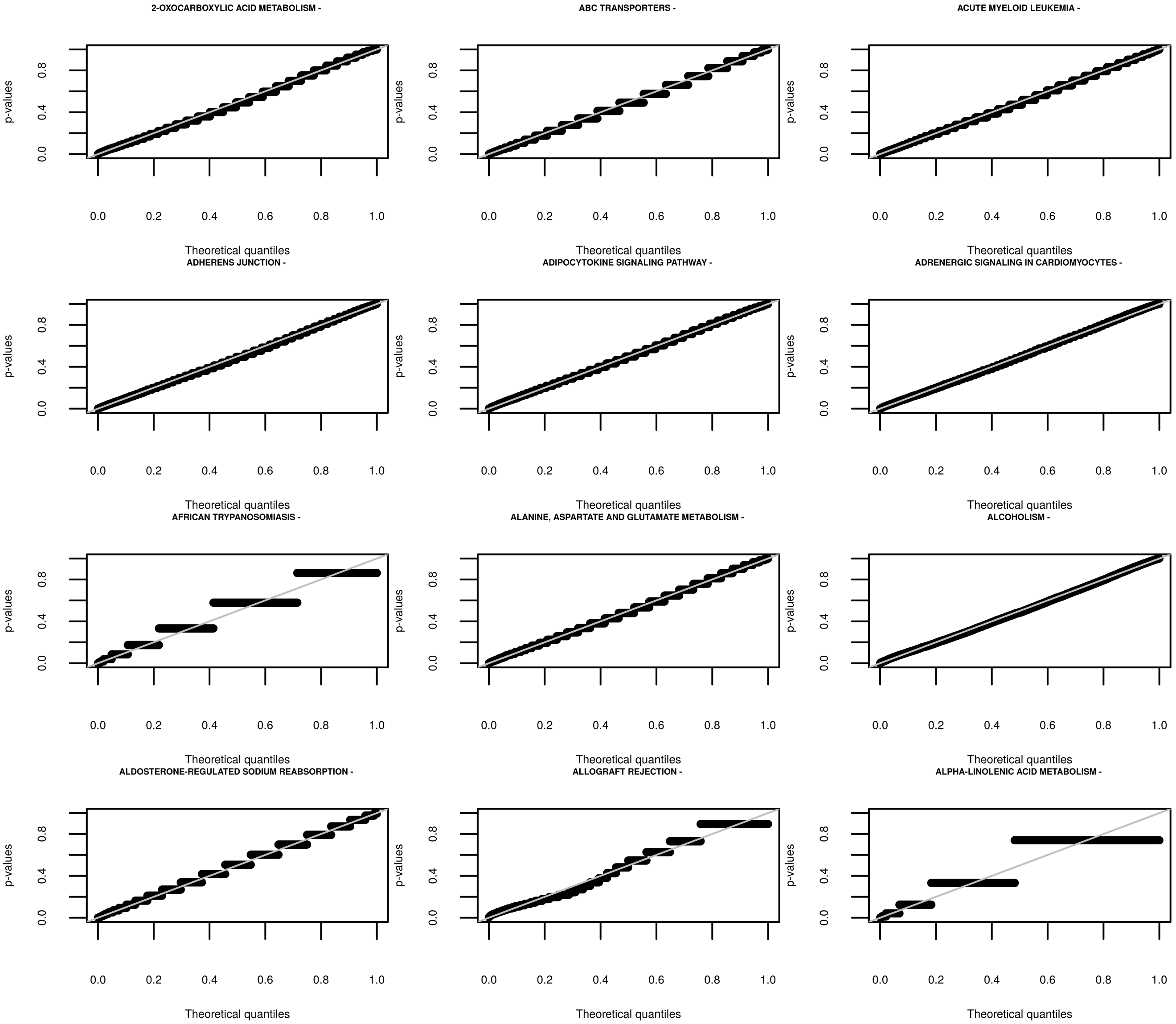

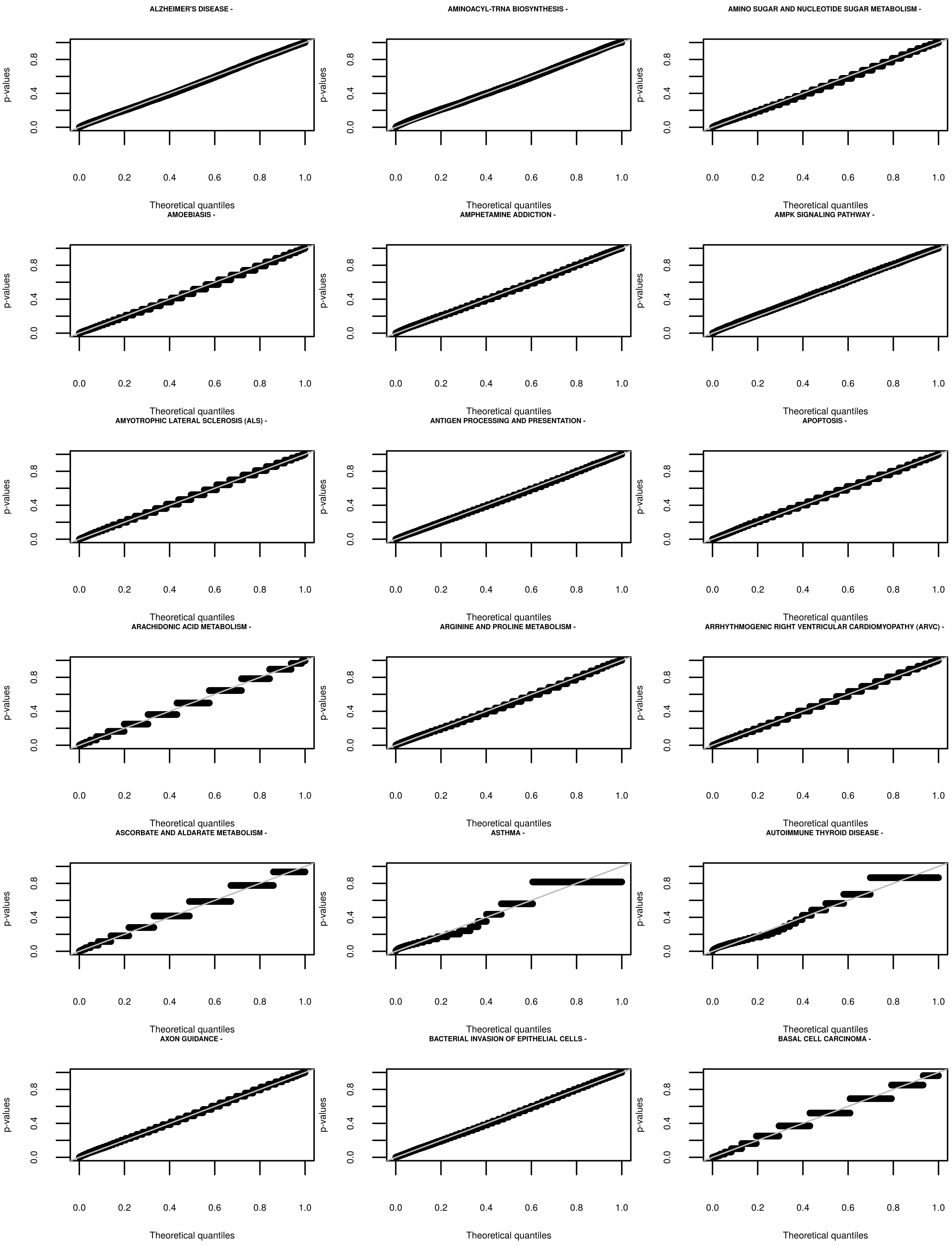
ALZHEIMER'S DISEASE -
AMINOACYL-TRNA BIOSYNTHESIS -
AMINO SUGAR AND NUCLEOTIDE SUGAR METABOLISM -
AMOEBIASIS -
AMPHETAMINE ADDICTION -
AMPK SIGNALING PATHWAY -
AMYOTROPHIC LATERAL SCLEROSIS (ALS) -
ANTIGEN PROCESSING AND PRESENTATION -
APOPTOSIS -
ARACHIDONIC ACID METABOLISM -
ARGININE AND PROLINE METABOLISM -
ARRHYTHMOGENIC RIGHT VENTRICULAR CARDIOMYOPATHY (ARVC) -
ASCORBATE AND ALDARATE METABOLISM -
ASTHMA -
AUTOIMMUNE THYROID DISEASE -
AXON GUIDANCE -
BACTERIAL INVASION OF EPITHELIAL CELLS -
BASAL CELL CARCINOMA -
p-values
0.0
0.4
0.8
0.0
0.2
0.4
0.6
0.8
1.0
Theoretical quantiles

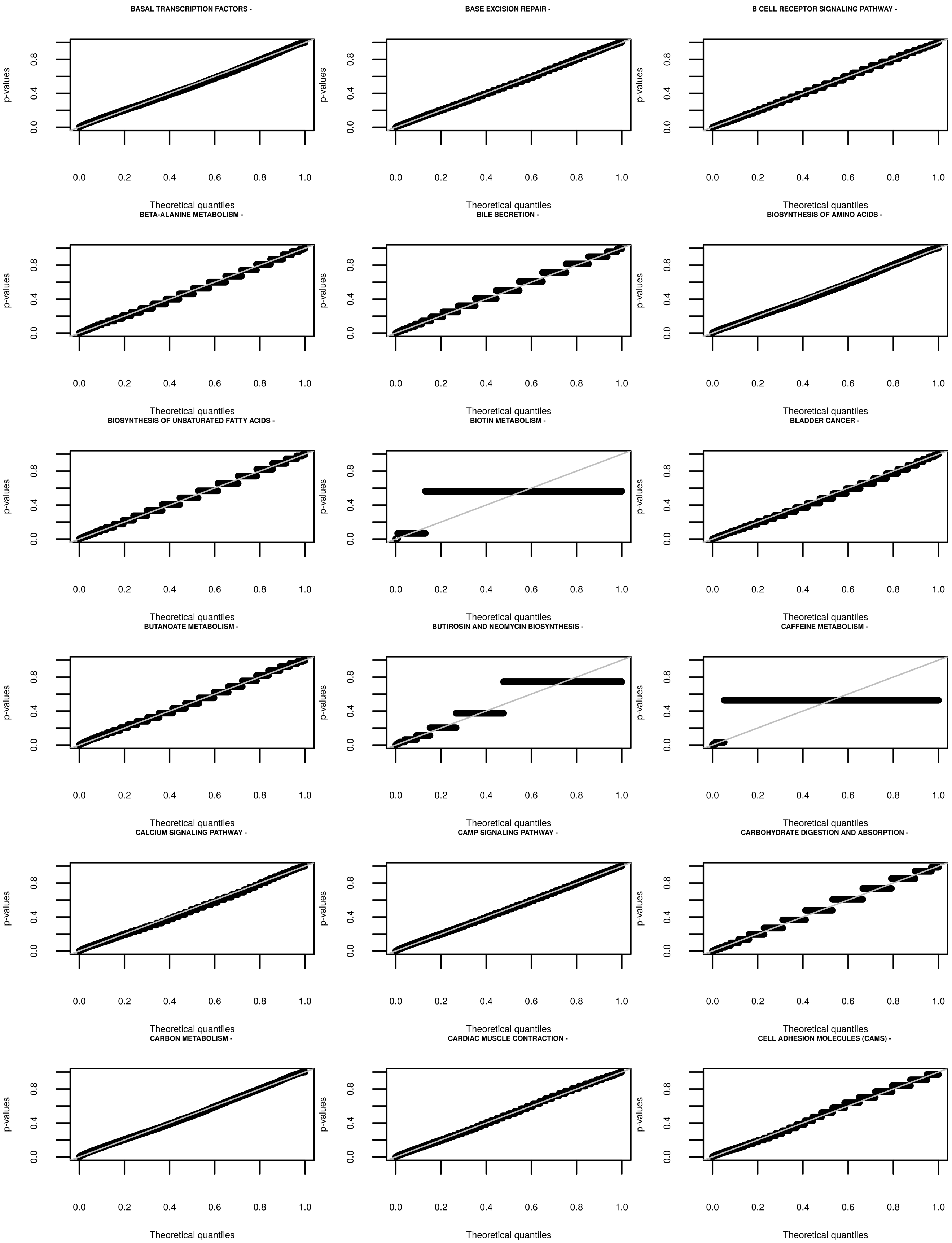
BASAL TRANSCRIPTION FACTORS -
BASE EXCISION REPAIR -
B CELL RECEPTOR SIGNALING PATHWAY -
BETA-ALANINE METABOLISM -
BILE SECRETION -
BIOSYNTHESIS OF AMINO ACIDS -
BIOSYNTHESIS OF UNSATURATED FATTY ACIDS -
BIOTIN METABOLISM -
BLADDER CANCER -
BUTANOATE METABOLISM -
BUTIROSIN AND NEOMYCIN BIOSYNTHESIS -
CAFFEINE METABOLISM -
CALCIUM SIGNALING PATHWAY -
CAMP SIGNALING PATHWAY -
CARBOHYDRATE DIGESTION AND ABSORPTION -
CARBON METABOLISM -
CARDIAC MUSCLE CONTRACTION -
CELL ADHESION MOLECULES (CAMS) -
p-values
0.0
0.4
0.8
Theoretical quantiles
0.0
0.2
0.4
0.6
0.8
1.0

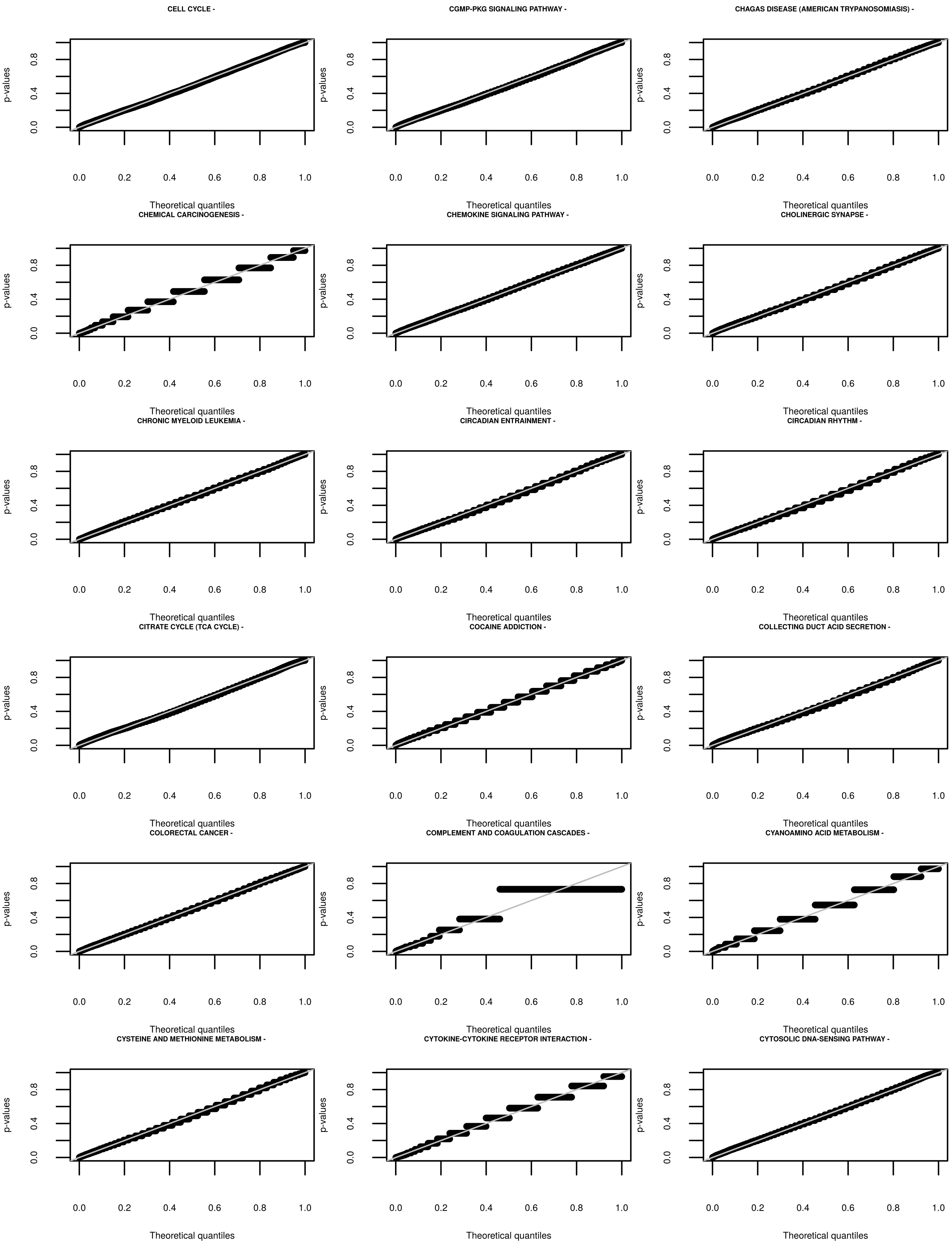
CELL CYCLE -
CGMP-PKG SIGNALING PATHWAY -
CHAGAS DISEASE (AMERICAN TRYPANOSOMIASIS) -
CHEMICAL CARCINOGENESIS -
CHEMOKINE SIGNALING PATHWAY -
CHOLINERGIC SYNAPSE -
CHRONIC MYELOID LEUKEMIA -
CIRCADIAN ENTRAINMENT -
CIRCADIAN RHYTHM -
CITRATE CYCLE (TCA CYCLE) -
COCAINE ADDICTION -
COLLECTING DUCT ACID SECRETION -
COLORECTAL CANCER -
COMPLEMENT AND COAGULATION CASCADES -
CYANOAMINO ACID METABOLISM -
CYSTEINE AND METHIONINE METABOLISM -
CYTOKINE-CYTOKINE RECEPTOR INTERACTION -
CYTOSOLIC DNA-SENSING PATHWAY -
p-values
0.0
0.4
0.8
Theoretical quantiles
0.0
0.2
0.4
0.6
0.8
1.0

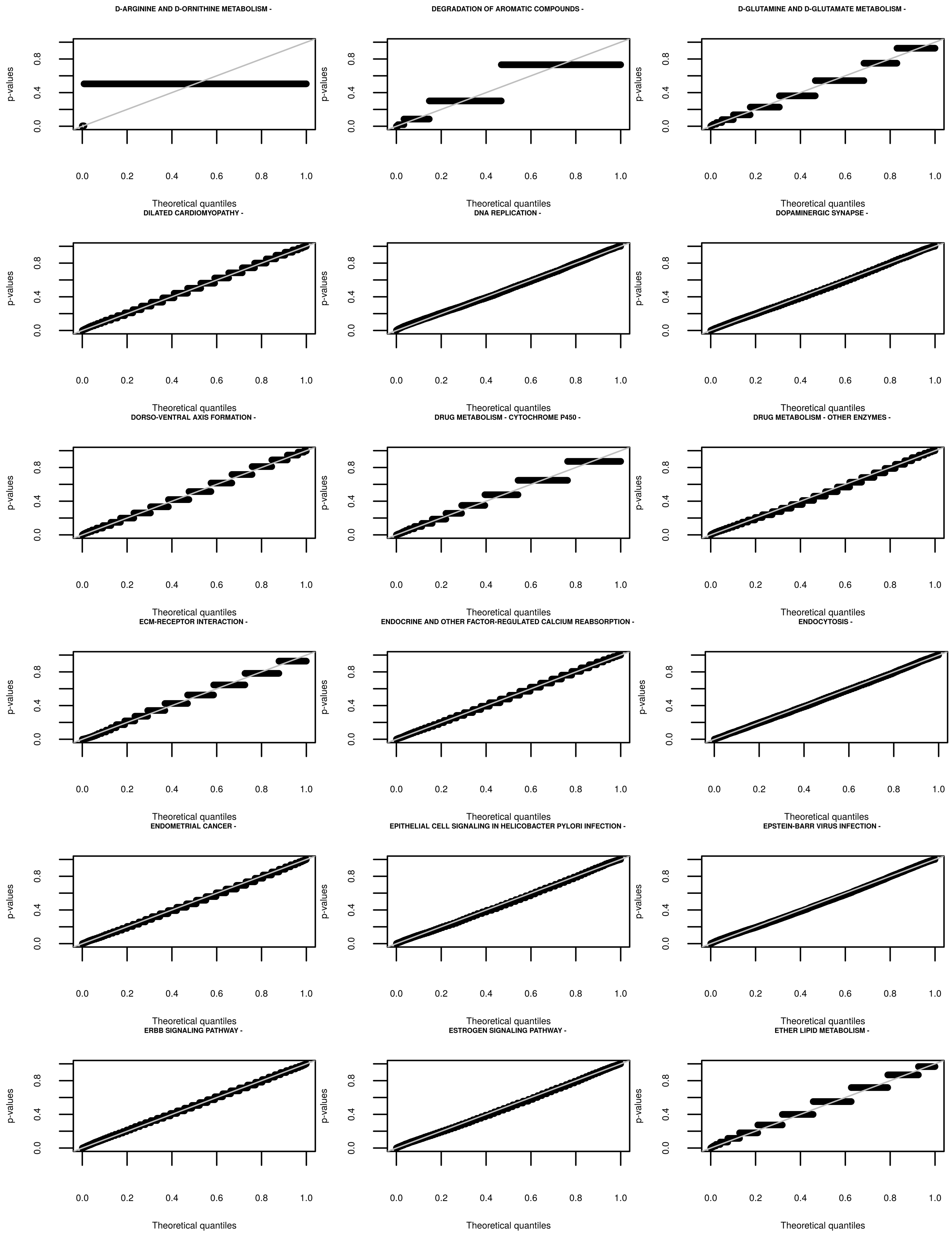
D-ARGININE AND D-ORNITHINE METABOLISM -
DEGRADATION OF AROMATIC COMPOUNDS -
D-GLUTAMINE AND D-GLUTAMATE METABOLISM -
DILATED CARDIOMYOPATHY -
DNA REPLICATION -
DOPAMINERGIC SYNAPSE -
DORSO-VENTRAL AXIS FORMATION -
DRUG METABOLISM - CYTOCHROME P450 -
DRUG METABOLISM - OTHER ENZYMES -
ECM-RECEPTOR INTERACTION -
ENDOCRINE AND OTHER FACTOR-REGULATED CALCIUM REABSORPTION -
ENDOCYTOSIS -
ENDOMETRIAL CANCER -
EPITHELIAL CELL SIGNALING IN HELICOBACTER PYLORI INFECTION -
EPSTEIN-BARR VIRUS INFECTION -
ERBB SIGNALING PATHWAY -
ESTROGEN SIGNALING PATHWAY -
ETHER LIPID METABOLISM -
p-values
0.0
0.4
0.8
Theoretical quantiles
0.0
0.2
0.4
0.6
0.8
1.0

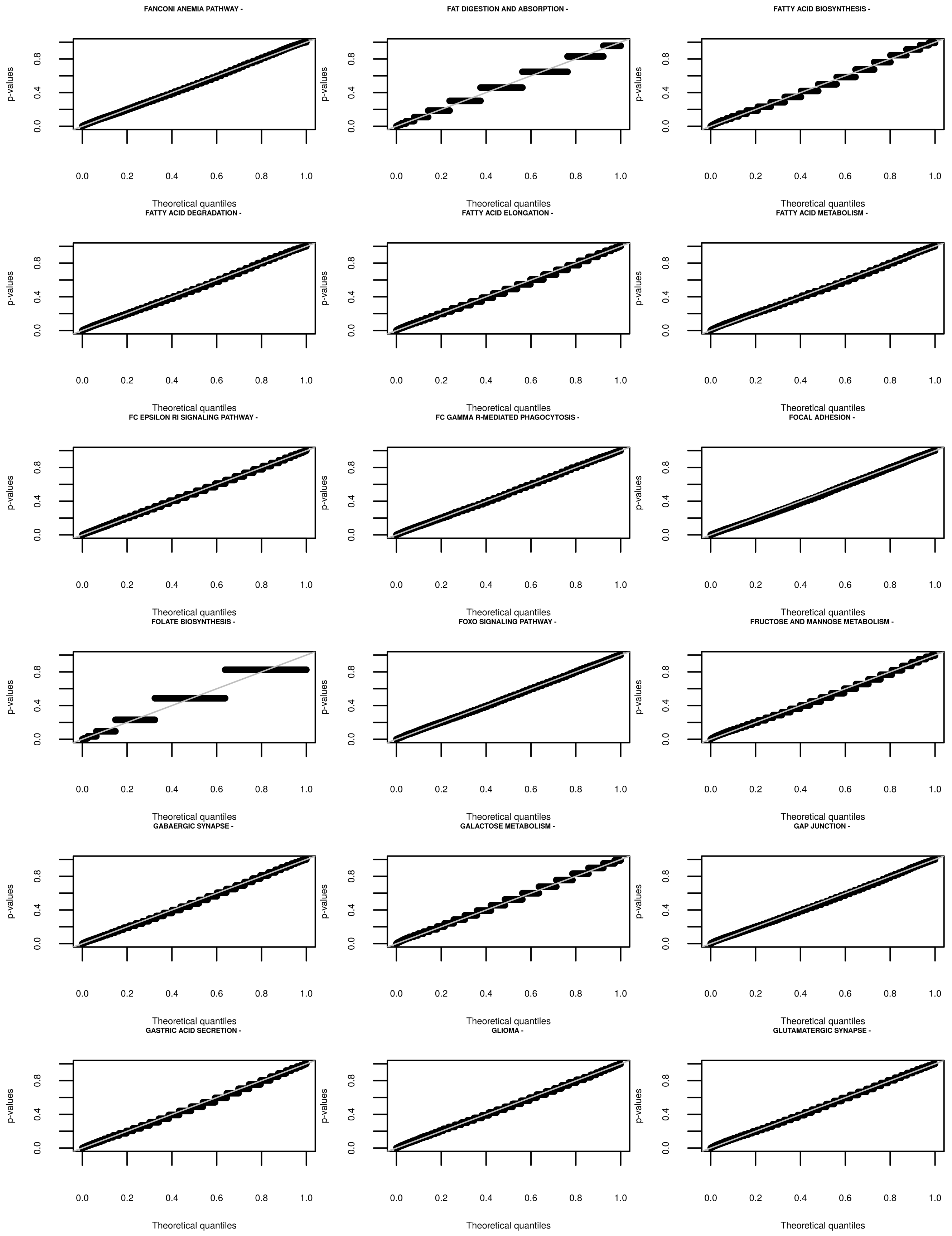
FANCONI ANEMIA PATHWAY -
FAT DIGESTION AND ABSORPTION -
FATTY ACID BIOSYNTHESIS -
FATTY ACID DEGRADATION -
FATTY ACID ELONGATION -
FATTY ACID METABOLISM -
FC EPSILON RI SIGNALING PATHWAY -
FC GAMMA R-MEDIATED PHAGOCYTOSIS -
FOCAL ADHESION -
FOLATE BIOSYNTHESIS -
FOXO SIGNALING PATHWAY -
FRUCTOSE AND MANNOSE METABOLISM -
GABAERGIC SYNAPSE -
GALACTOSE METABOLISM -
GAP JUNCTION -
GASTRIC ACID SECRETION -
GLIOMA -
GLUTAMATERGIC SYNAPSE -
p-values
0.0
0.4
0.8
Theoretical quantiles
0.0
0.2
0.4
0.6
0.8
1.0

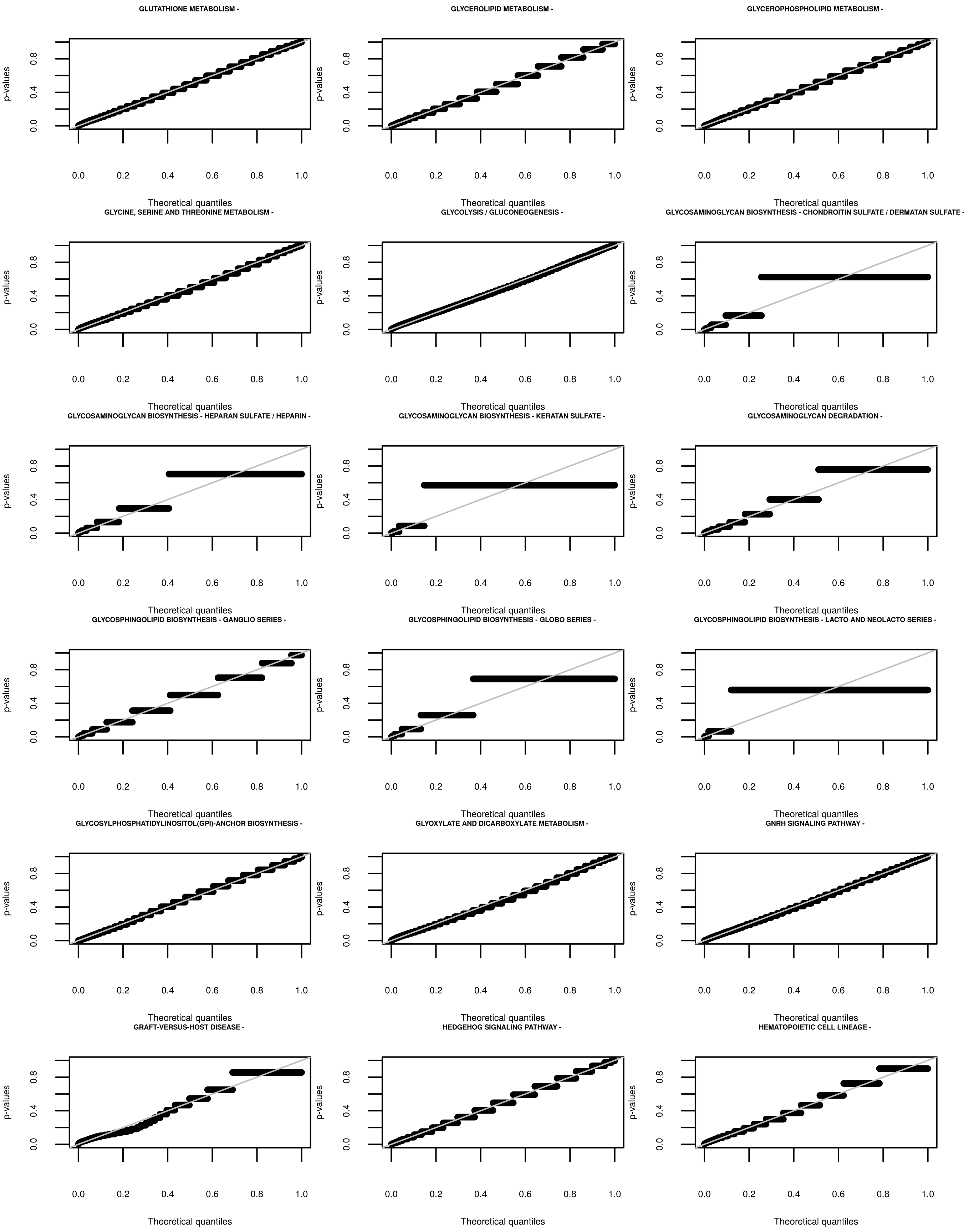
GLUTATHIONE METABOLISM -
GLYCEROLIPID METABOLISM -
GLYCEROPHOSPHOLIPID METABOLISM -
GLYCINE, SERINE AND THREONINE METABOLISM -
GLYCOLYSIS / GLUCONEOGENESIS -
GLYCOSAMINOGLYCAN BIOSYNTHESIS - CHONDROITIN SULFATE / DERMATAN SULFATE -
GLYCOSAMINOGLYCAN BIOSYNTHESIS - HEPARAN SULFATE / HEPARIN -
GLYCOSAMINOGLYCAN BIOSYNTHESIS - KERATAN SULFATE -
GLYCOSAMINOGLYCAN DEGRADATION -
GLYCOSPHINGOLIPID BIOSYNTHESIS - GANGLIO SERIES -
GLYCOSPHINGOLIPID BIOSYNTHESIS - GLOBO SERIES -
GLYCOSPHINGOLIPID BIOSYNTHESIS - LACTO AND NEOLACTO SERIES -
GLYCOSYLPHOSPHATIDYLINOSITOL(GPI)-ANCHOR BIOSYNTHESIS -
GLYOXYLATE AND DICARBOXYLATE METABOLISM -
GNRH SIGNALING PATHWAY -
GRAFT-VERSUS-HOST DISEASE -
HEDGEHOG SIGNALING PATHWAY -
HEMATOPOIETIC CELL LINEAGE -
p-values
0.0
0.4
0.8
0.0
0.2
0.4
0.6
0.8
1.0
Theoretical quantiles

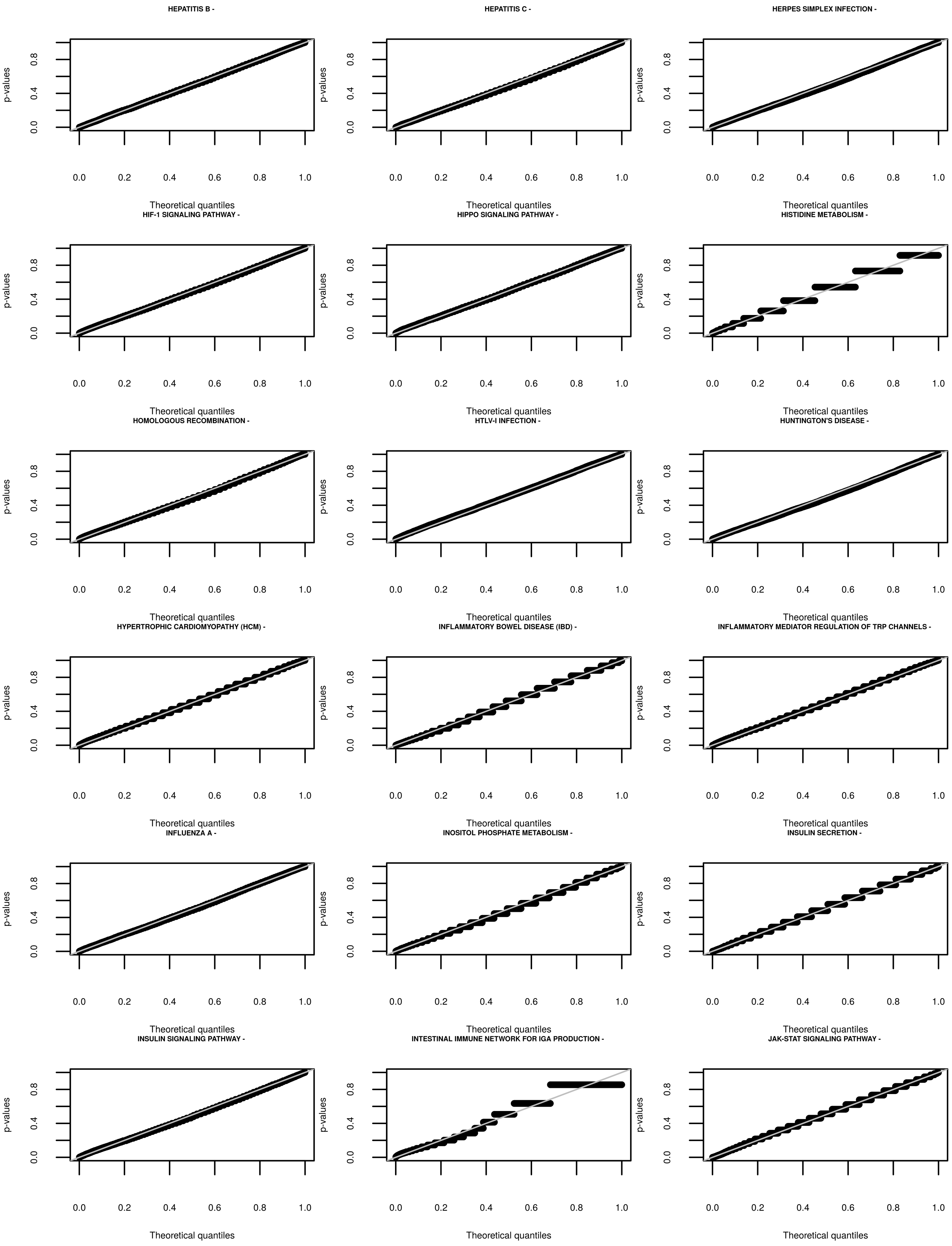
HEPATITIS B -
HEPATITIS C -
HERPES SIMPLEX INFECTION -
HIF-1 SIGNALING PATHWAY -
HIPPO SIGNALING PATHWAY -
HISTIDINE METABOLISM -
HOMOLOGOUS RECOMBINATION -
HTLV-I INFECTION -
HUNTINGTON'S DISEASE -
HYPERTROPHIC CARDIOMYOPATHY (HCM) -
INFLAMMATORY BOWEL DISEASE (IBD) -
INFLAMMATORY MEDIATOR REGULATION OF TRP CHANNELS -
INFLUENZA A -
INOSITOL PHOSPHATE METABOLISM -
INSULIN SECRETION -
INSULIN SIGNALING PATHWAY -
INTESTINAL IMMUNE NETWORK FOR IGA PRODUCTION -
JAK-STAT SIGNALING PATHWAY -
p-values
0.0
0.4
0.8
Theoretical quantiles
0.0
0.2
0.4
0.6
0.8
1.0

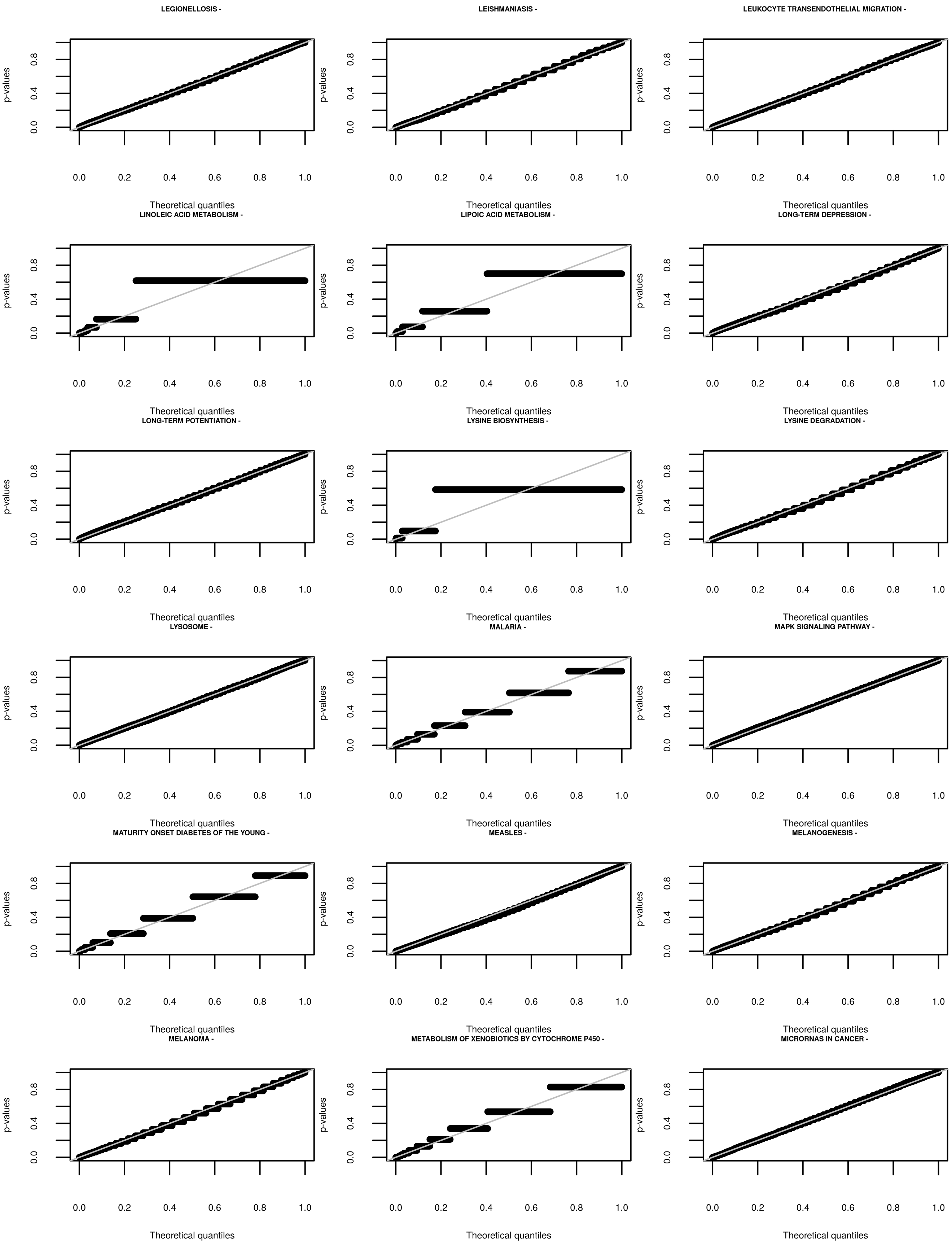
LEGIONELLOSIS -
LEISHMANIASIS -
LEUKOCYTE TRANSENDOTHELIAL MIGRATION -
LINOLEIC ACID METABOLISM -
LIPOIC ACID METABOLISM -
LONG-TERM DEPRESSION -
LONG-TERM POTENTIATION -
LYSINE BIOSYNTHESIS -
LYSINE DEGRADATION -
LYSOSOME -
MALARIA -
MAPK SIGNALING PATHWAY -
MATURITY ONSET DIABETES OF THE YOUNG -
MEASLES -
MELANOGENESIS -
MELANOMA -
METABOLISM OF XENOBIOTICS BY CYTOCHROME P450 -
MICRORNAS IN CANCER -
p-values
Theoretical quantiles
0.0
0.2
0.4
0.6
0.8
1.0

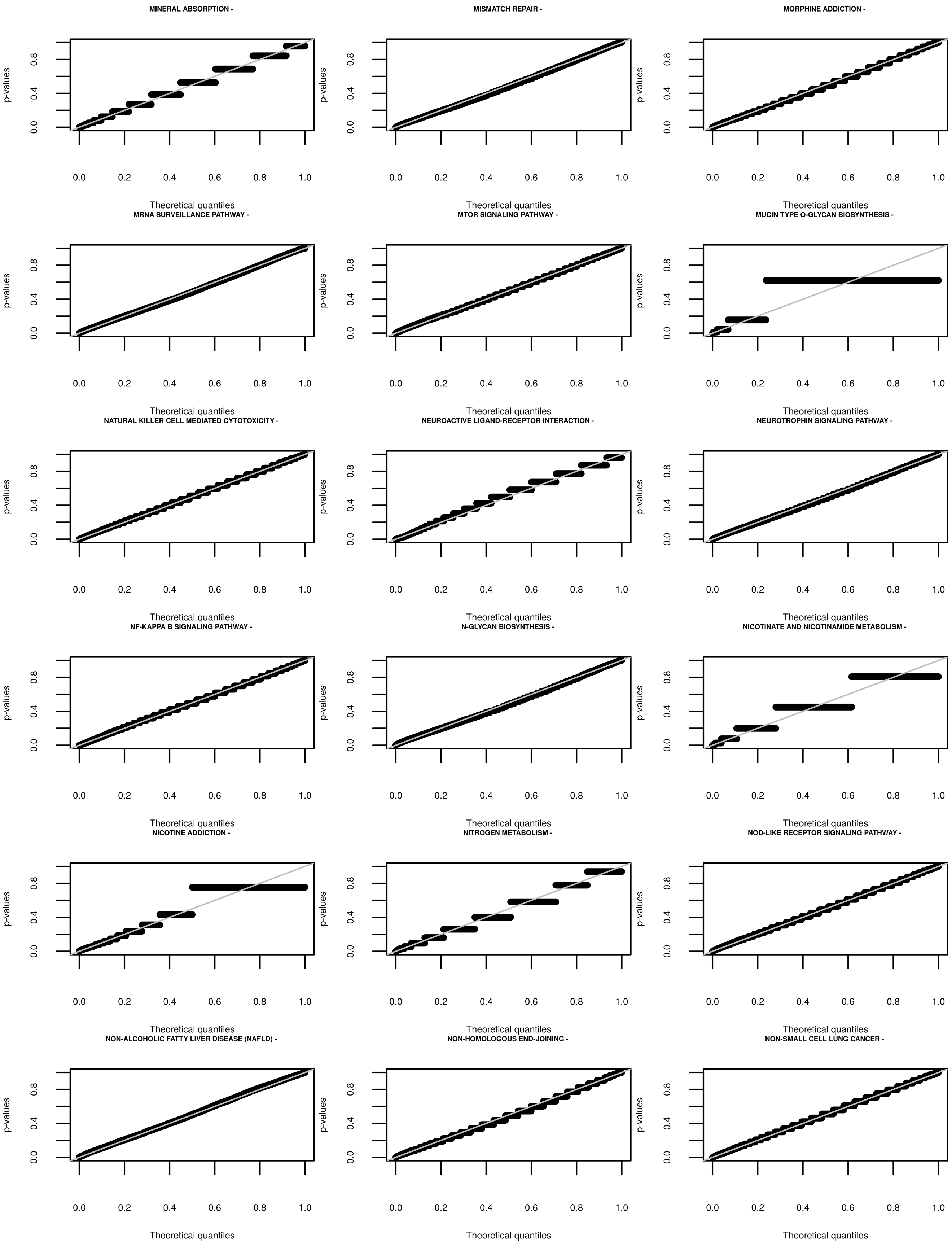
MINERAL ABSORPTION -
MISMATCH REPAIR -
MORPHINE ADDICTION -
MRNA SURVEILLANCE PATHWAY -
MTOR SIGNALING PATHWAY -
MUCIN TYPE O-GLYCAN BIOSYNTHESIS -
NATURAL KILLER CELL MEDIATED CYTOTOXICITY -
NEUROACTIVE LIGAND-RECEPTOR INTERACTION -
NEUROTROPHIN SIGNALING PATHWAY -
NF-KAPPA B SIGNALING PATHWAY -
N-GLYCAN BIOSYNTHESIS -
NICOTINATE AND NICOTINAMIDE METABOLISM -
NICOTINE ADDICTION -
NITROGEN METABOLISM -
NOD-LIKE RECEPTOR SIGNALING PATHWAY -
NON-ALCOHOLIC FATTY LIVER DISEASE (NAFLD) -
NON-HOMOLOGOUS END-JOINING -
NON-SMALL CELL LUNG CANCER -
p-values
0.0
0.4
0.8
Theoretical quantiles
0.0
0.2
0.4
0.6
0.8
1.0

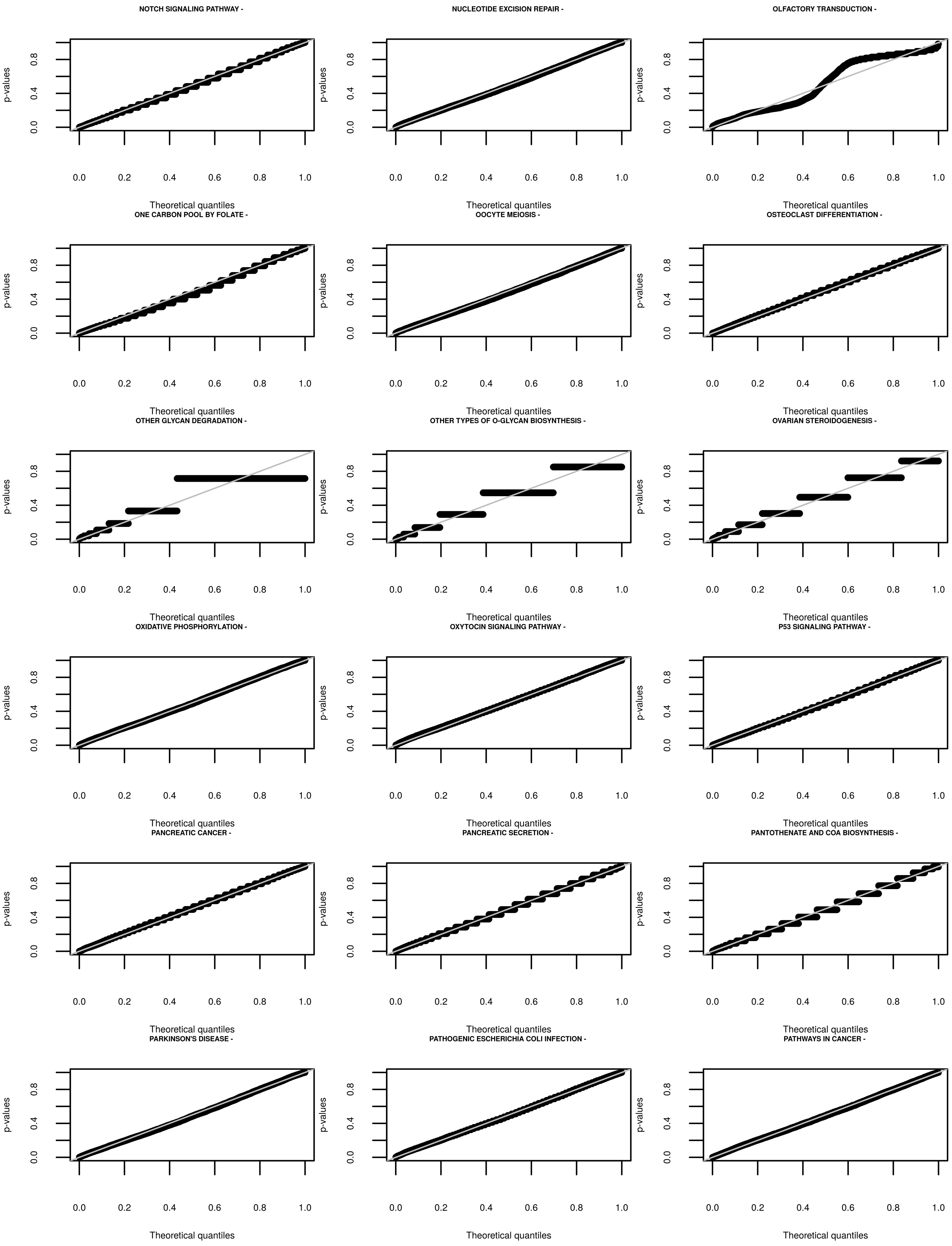
NOTCH SIGNALING PATHWAY -
NUCLEOTIDE EXCISION REPAIR -
OLFACTORY TRANSDUCTION -
ONE CARBON POOL BY FOLATE -
OOCYTE MEIOSIS -
OSTEOCLAST DIFFERENTIATION -
OTHER GLYCAN DEGRADATION -
OTHER TYPES OF O-GLYCAN BIOSYNTHESIS -
OVARIAN STEROIDOGENESIS -
OXIDATIVE PHOSPHORYLATION -
OXYTOCIN SIGNALING PATHWAY -
P53 SIGNALING PATHWAY -
PANCREATIC CANCER -
PANCREATIC SECRETION -
PANTOTHENATE AND COA BIOSYNTHESIS -
PARKINSON'S DISEASE -
PATHOGENIC ESCHERICHIA COLI INFECTION -
PATHWAYS IN CANCER -
p-values
Theoretical quantiles

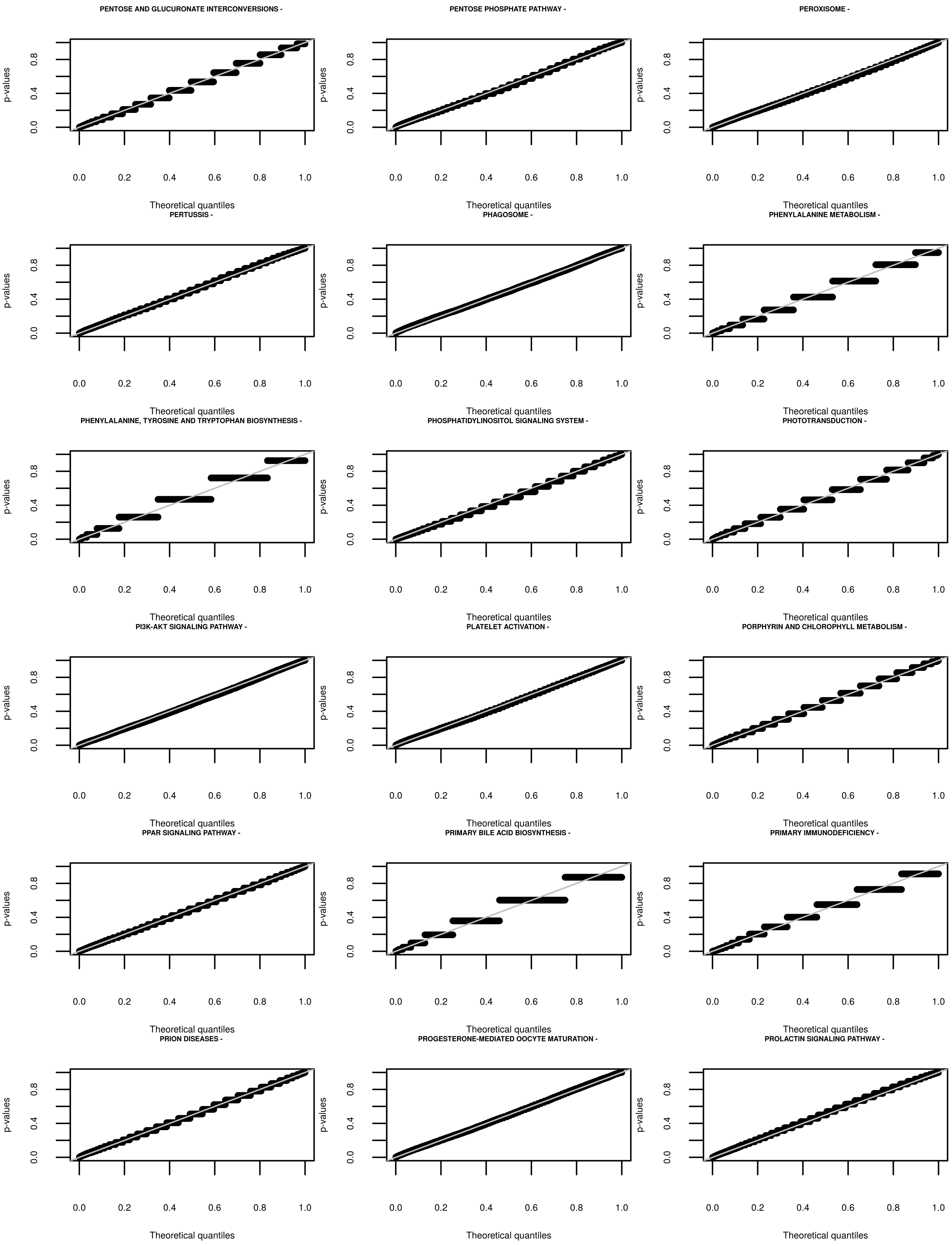
PENTOSE AND GLUCURONATE INTERCONVERSIONS -
PENTOSE PHOSPHATE PATHWAY -
PEROXISOME -
PERTUSSIS -
PHAGOSOME -
PHENYLALANINE METABOLISM -
PHENYLALANINE, TYROSINE AND TRYPTOPHAN BIOSYNTHESIS -
PHOSPHATIDYLINOSITOL SIGNALING SYSTEM -
PHOTOTRANSDUCTION -
PI3K-AKT SIGNALING PATHWAY -
PLATELET ACTIVATION -
PORPHYRIN AND CHLOROPHYLL METABOLISM -
PPAR SIGNALING PATHWAY -
PRIMARY BILE ACID BIOSYNTHESIS -
PRIMARY IMMUNODEFICIENCY -
PRION DISEASES -
PROGESTERONE-MEDIATED OOCYTE MATURATION -
PROLACTIN SIGNALING PATHWAY -
p-values
0.0
0.4
0.8
Theoretical quantiles
0.0
0.2
0.4
0.6
0.8
1.0

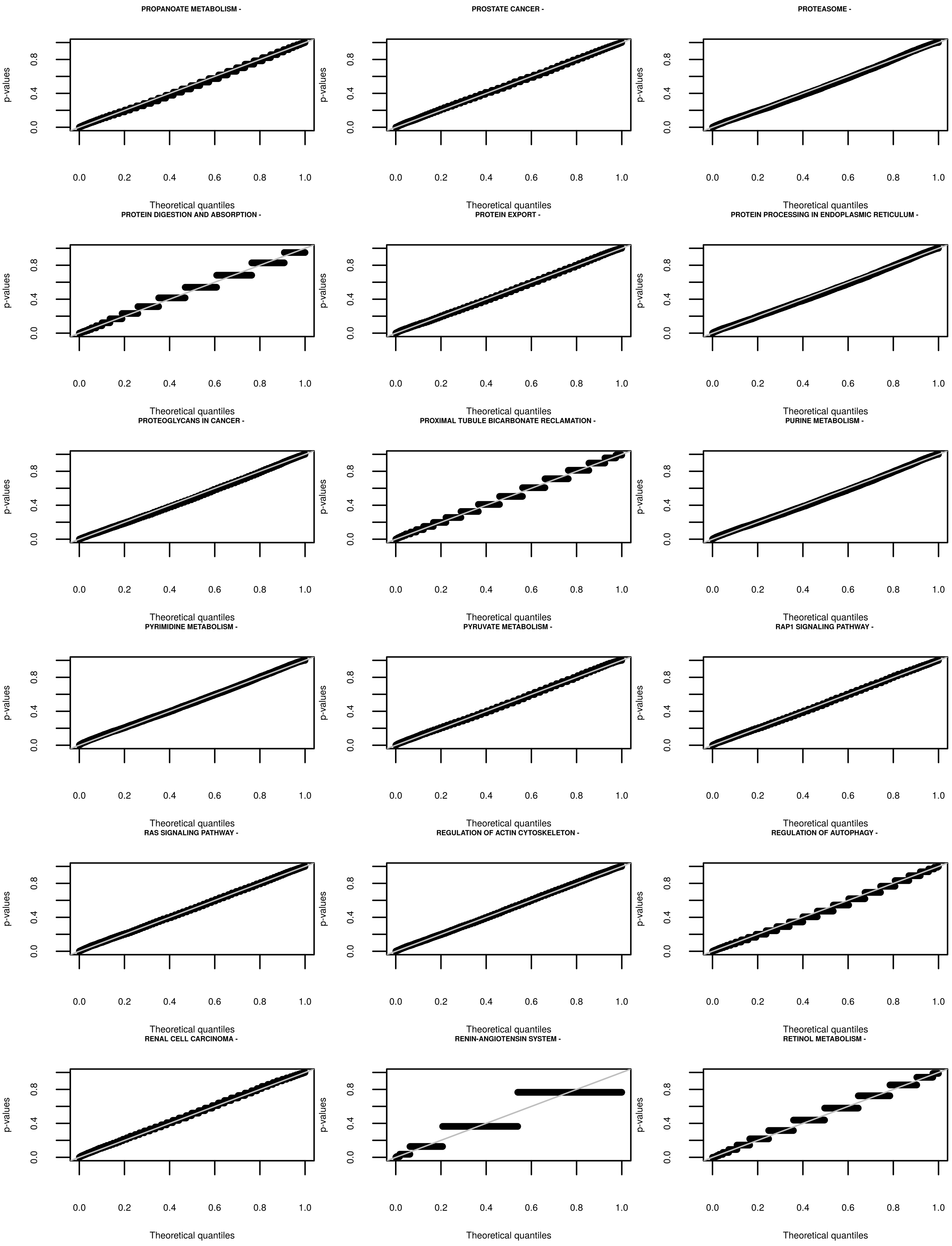
PROPANOATE METABOLISM -
PROSTATE CANCER -
PROTEASOME -
PROTEIN DIGESTION AND ABSORPTION -
PROTEIN EXPORT -
PROTEIN PROCESSING IN ENDOPLASMIC RETICULUM -
PROTEOGLYCANS IN CANCER -
PROXIMAL TUBULE BICARBONATE RECLAMATION -
PURINE METABOLISM -
PYRIMIDINE METABOLISM -
PYRUVATE METABOLISM -
RAP1 SIGNALING PATHWAY -
RAS SIGNALING PATHWAY -
REGULATION OF ACTIN CYTOSKELETON -
REGULATION OF AUTOPHAGY -
RENAL CELL CARCINOMA -
RENIN-ANGIOTENSIN SYSTEM -
RETINOL METABOLISM -
p-values
0.0
0.4
0.8
Theoretical quantiles
0.0
0.2
0.4
0.6
0.8
1.0

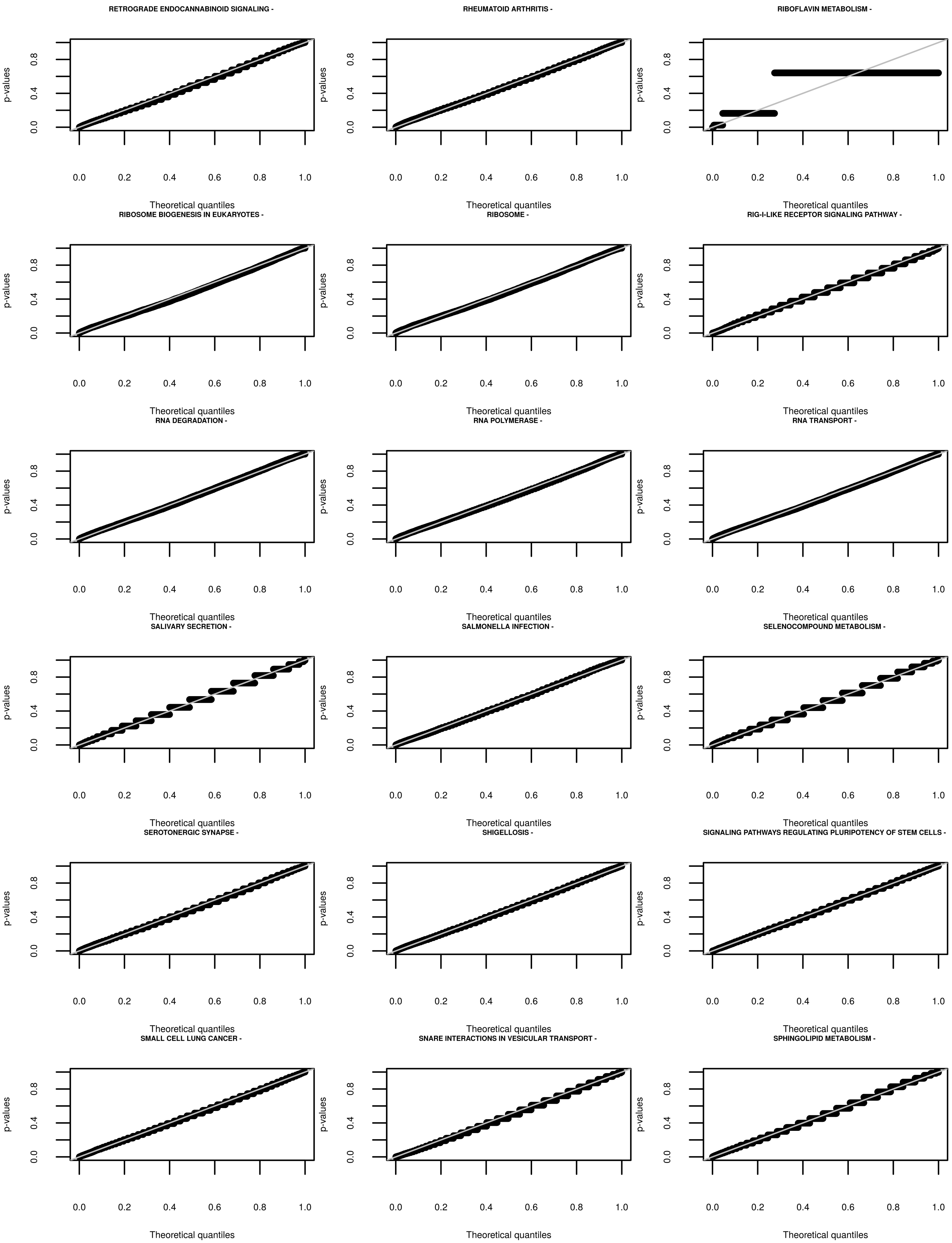
RETROGRADE ENDOCANNABINOID SIGNALING -
RHEUMATOID ARTHRITIS -
RIBOFLAVIN METABOLISM -
RIBOSOME BIOGENESIS IN EUKARYOTES -
RIBOSOME -
RIG-I-LIKE RECEPTOR SIGNALING PATHWAY -
RNA DEGRADATION -
RNA POLYMERASE -
RNA TRANSPORT -
SALIVARY SECRETION -
SALMONELLA INFECTION -
SELENOCOMPOUND METABOLISM -
SEROTONERGIC SYNAPSE -
SHIGELLOSIS -
SIGNALING PATHWAYS REGULATING PLURIPOTENCY OF STEM CELLS -
SMALL CELL LUNG CANCER -
SNARE INTERACTIONS IN VESICULAR TRANSPORT -
SPHINGOLIPID METABOLISM -
p-values
Theoretical quantiles
0.0
0.4
0.8
0.2
0.6
1.0

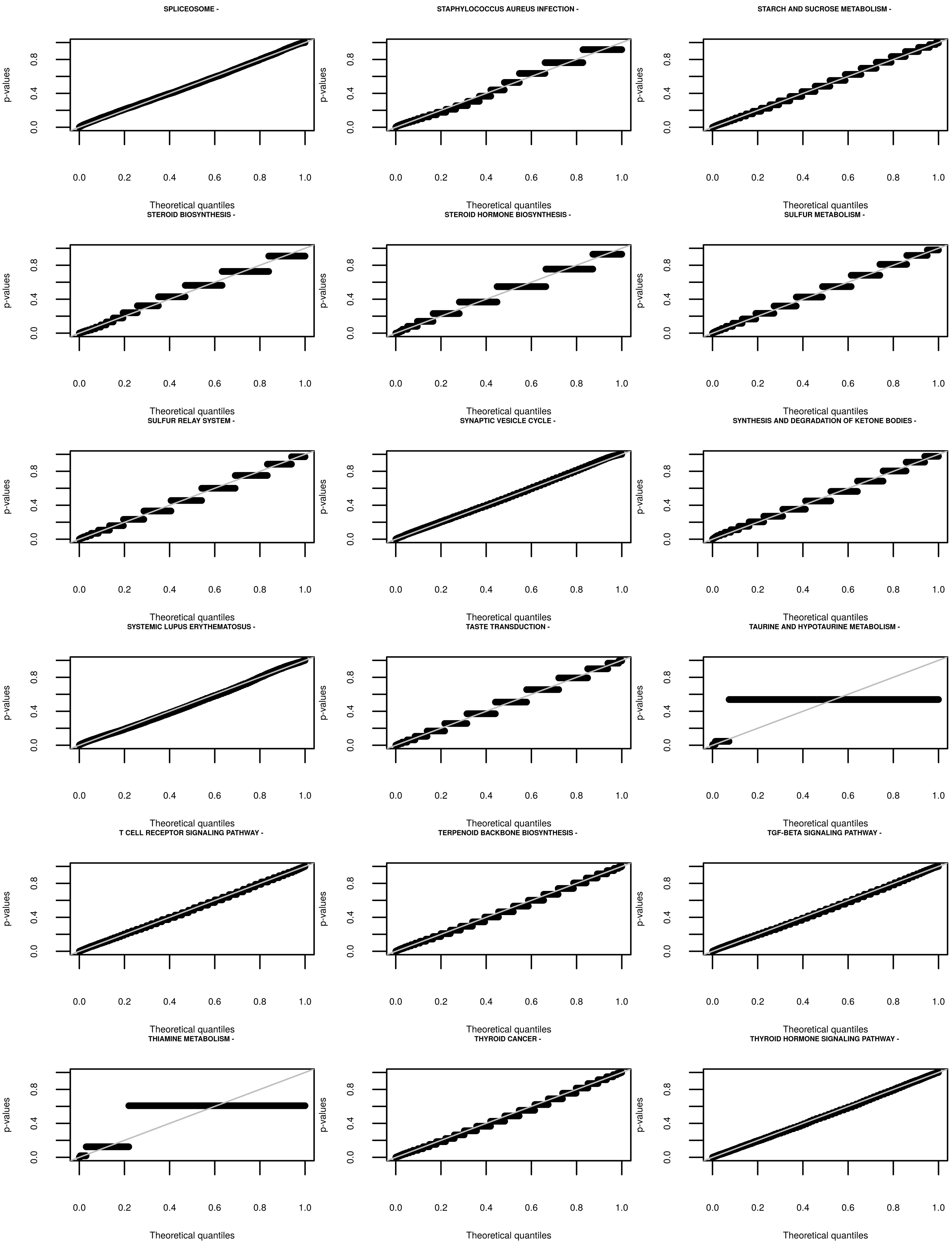
SPLICEOSOME -
STAPHYLOCOCCUS AUREUS INFECTION -
STARCH AND SUCROSE METABOLISM -
STEROID BIOSYNTHESIS -
STEROID HORMONE BIOSYNTHESIS -
SULFUR METABOLISM -
SULFUR RELAY SYSTEM -
SYNAPTIC VESICLE CYCLE -
SYNTHESIS AND DEGRADATION OF KETONE BODIES -
SYSTEMIC LUPUS ERYTHEMATOSUS -
TASTE TRANSDUCTION -
TAURINE AND HYPOTAURINE METABOLISM -
T CELL RECEPTOR SIGNALING PATHWAY -
TERPENOID BACKBONE BIOSYNTHESIS -
TGF-BETA SIGNALING PATHWAY -
THIAMINE METABOLISM -
THYROID CANCER -
THYROID HORMONE SIGNALING PATHWAY -
p-values
0.0
0.4
0.8
Theoretical quantiles
0.0
0.2
0.4
0.6
0.8
1.0

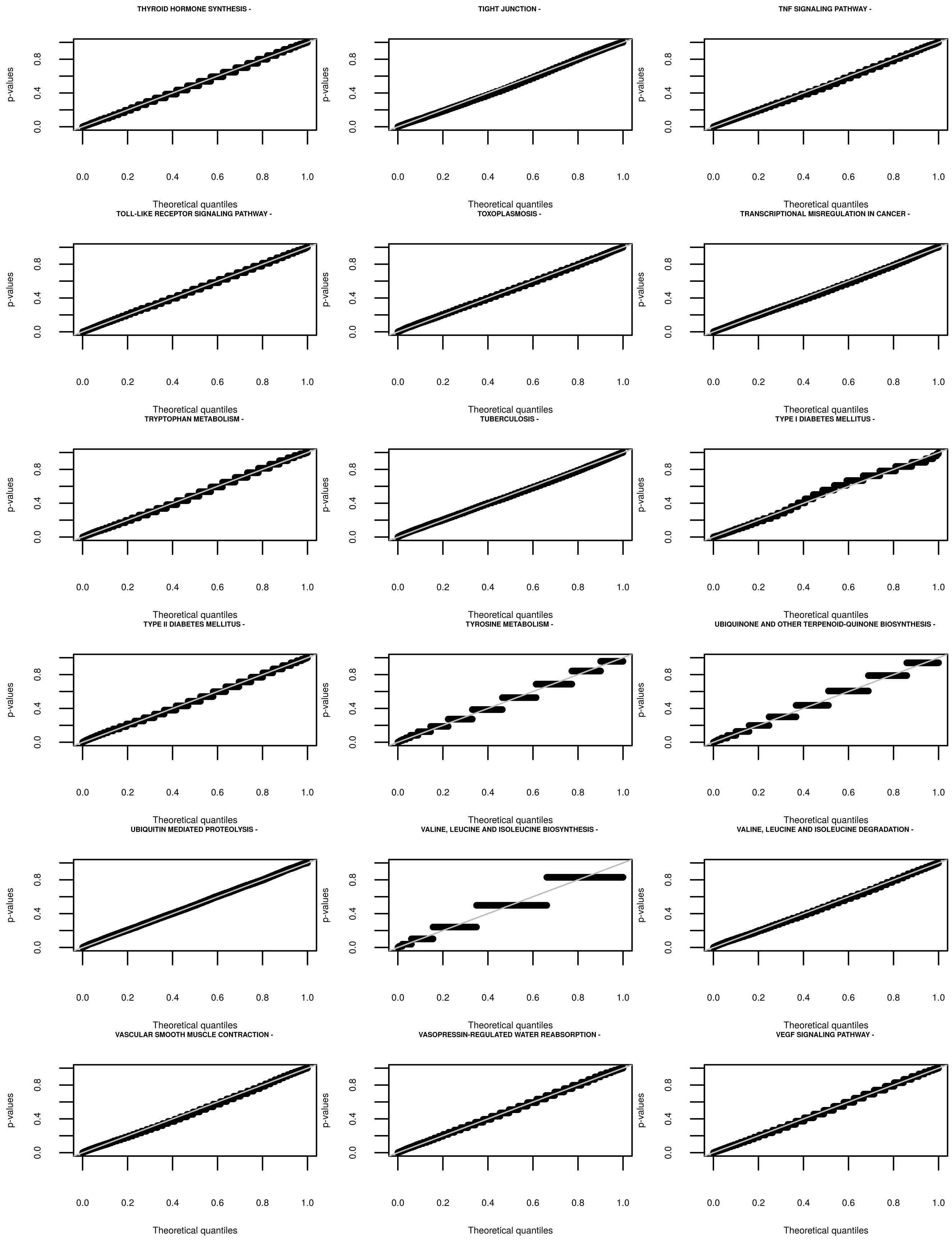

THYROID HORMONE SYNTHESIS -
TIGHT JUNCTION -
TNF SIGNALING PATHWAY -
TOLL-LIKE RECEPTOR SIGNALING PATHWAY -
TOXOPLASMOSIS -
TRANSCRIPTIONAL MISREGULATION IN CANCER -
TRYPTOPHAN METABOLISM -
TUBERCULOSIS -
TYPE I DIABETES MELLITUS -
TYPE II DIABETES MELLITUS -
TYROSINE METABOLISM -
UBIQUINONE AND OTHER TERPENOID-QUINONE BIOSYNTHESIS -
UBIQUITIN MEDIATED PROTEOLYSIS -
VALINE, LEUCINE AND ISOLEUCINE BIOSYNTHESIS -
VALINE, LEUCINE AND ISOLEUCINE DEGRADATION -
VASCULAR SMOOTH MUSCLE CONTRACTION -
VASOPRESSIN-REGULATED WATER REABSORPTION -
VEGF SIGNALING PATHWAY -
p-values
Theoretical quantiles

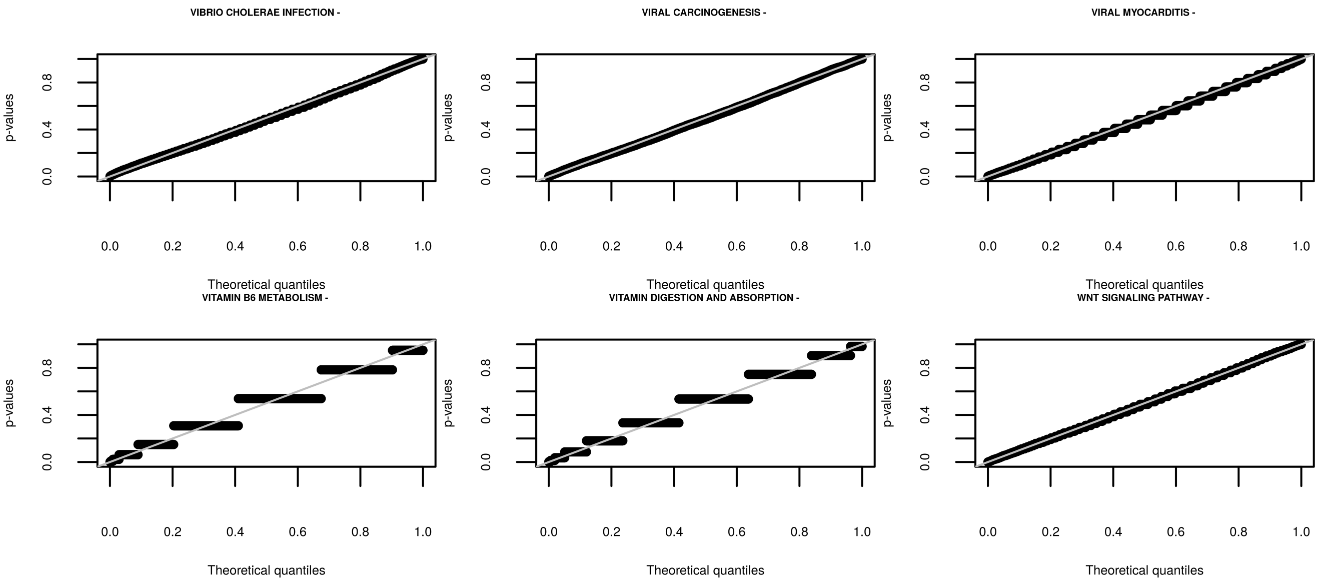
VIBRIO CHOLERAE INFECTION -
p-values
0.0
0.4
0.8
0.0
0.2
0.4
0.6
0.8
1.0
Theoretical quantiles
VIRAL CARCINOGENESIS -
p-values
0.0
0.4
0.8
0.0
0.2
0.4
0.6
0.8
1.0
Theoretical quantiles
VIRAL MYOCARDITIS -
p-values
0.0
0.4
0.8
0.0
0.2
0.4
0.6
0.8
1.0
Theoretical quantiles
VITAMIN B6 METABOLISM -
p-values
0.0
0.4
0.8
0.0
0.2
0.4
0.6
0.8
1.0
Theoretical quantiles
VITAMIN DIGESTION AND ABSORPTION -
p-values
0.0
0.4
0.8
0.0
0.2
0.4
0.6
0.8
1.0
Theoretical quantiles
WNT SIGNALING PATHWAY -
p-values
0.0
0.4
0.8
0.0
0.2
0.4
0.6
0.8
1.0
Theoretical quantiles

Pathway-specific model estimation for improved pathway annotation by network crosstalk

*Miguel Castresana-Aguirre, Erik L.L. Sonnhammer**

Department of Biochemistry and Biophysics, Stockholm University, Science for Life Laboratory, Box 1031, 17121 Solna, Sweden,
*To whom correspondence should be addressed: erik.sonnhammer@dbb.su.se

Supplementary Figure 3: 10000 random gene sets of size 200 are run against KEGG pathways. P-value uniformity test of ANUBIX. Reported p-values are plotted against theoretical quantile (rank). Software: R version 3.4.3 https://www.r-project.org/.

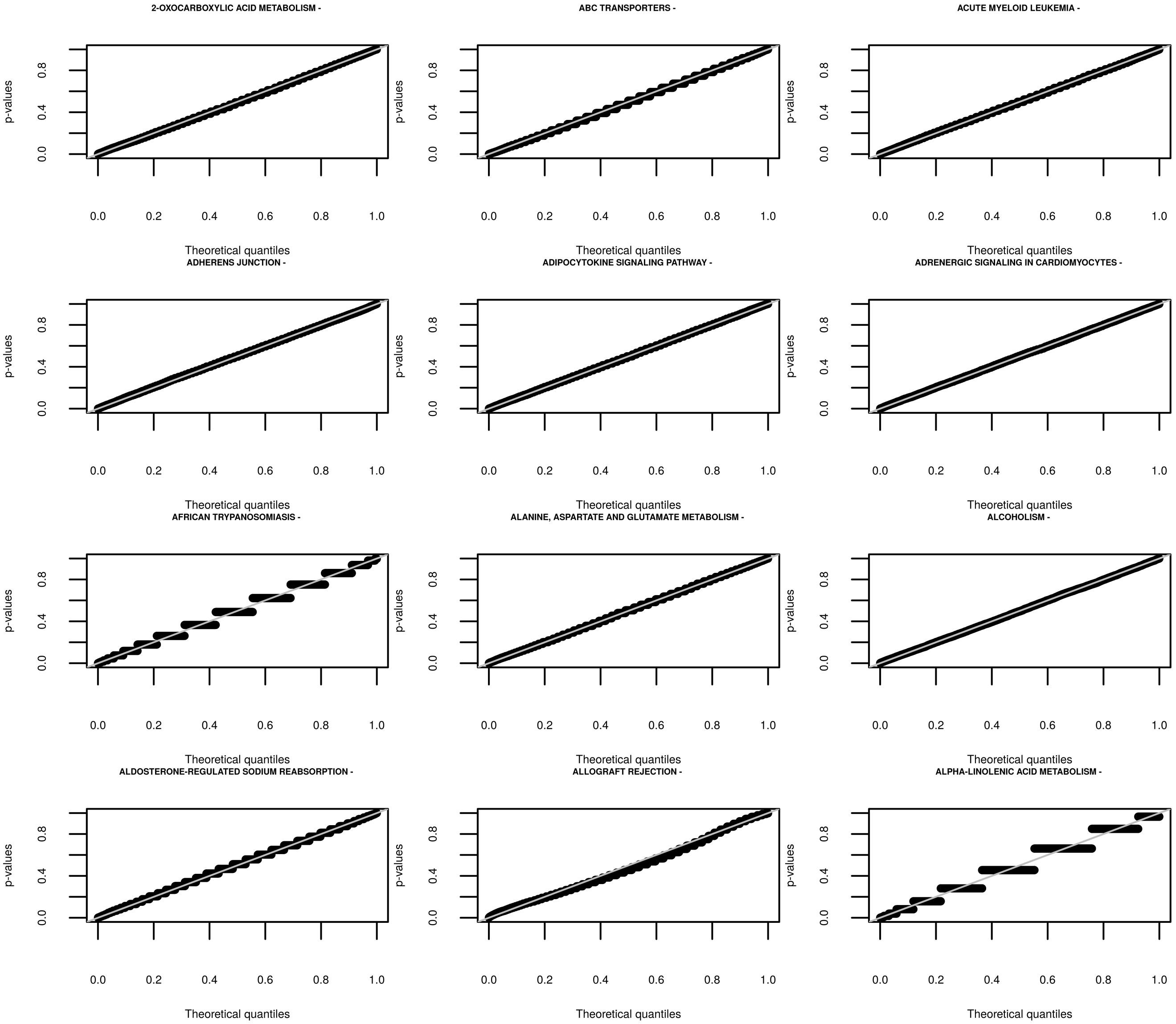

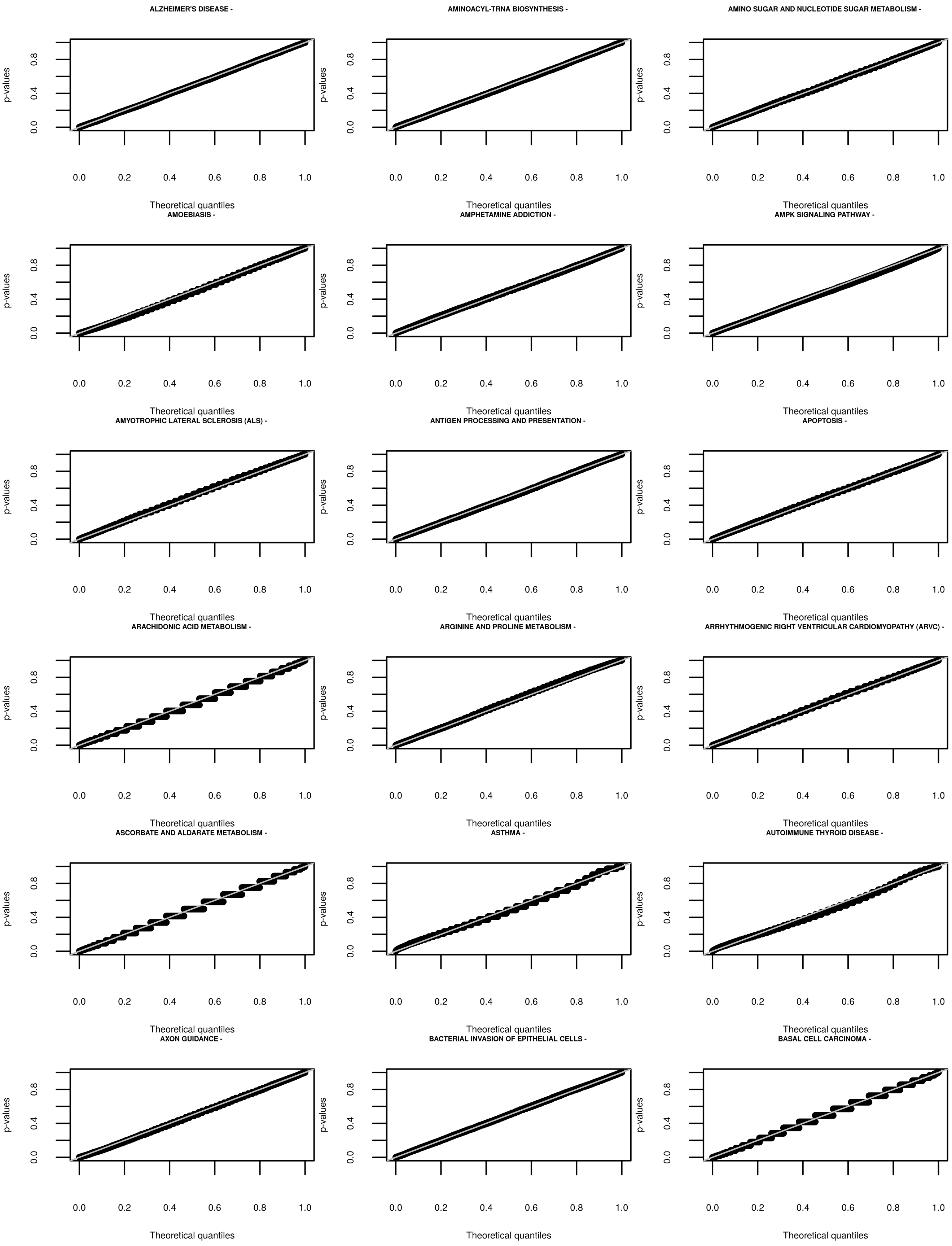
ALZHEIMER'S DISEASE -
AMINOACYL-TRNA BIOSYNTHESIS -
AMINO SUGAR AND NUCLEOTIDE SUGAR METABOLISM -
AMOEBIASIS -
AMPHETAMINE ADDICTION -
AMPK SIGNALING PATHWAY -
AMYOTROPHIC LATERAL SCLEROSIS (ALS) -
ANTIGEN PROCESSING AND PRESENTATION -
APOPTOSIS -
ARACHIDONIC ACID METABOLISM -
ARGININE AND PROLINE METABOLISM -
ARRHYTHMOGENIC RIGHT VENTRICULAR CARDIOMYOPATHY (ARVC) -
ASCORBATE AND ALDARATE METABOLISM -
ASTHMA -
AUTOIMMUNE THYROID DISEASE -
AXON GUIDANCE -
BACTERIAL INVASION OF EPITHELIAL CELLS -
BASAL CELL CARCINOMA -
p-values
0.0
0.4
0.8
0.0
0.2
0.4
0.6
0.8
1.0
Theoretical quantiles

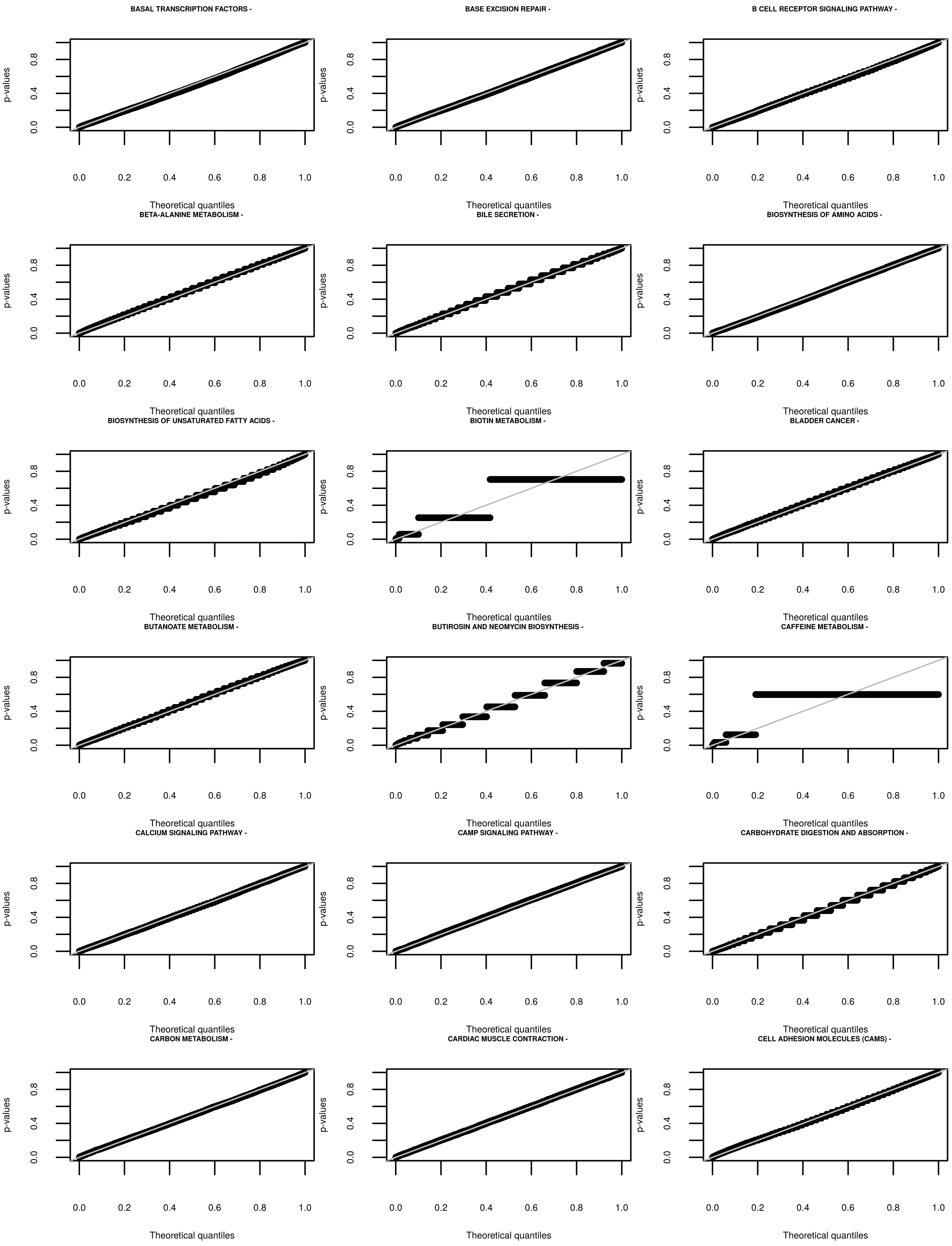
BASAL TRANSCRIPTION FACTORS -
BASE EXCISION REPAIR -
B CELL RECEPTOR SIGNALING PATHWAY -
BETA-ALANINE METABOLISM -
BILE SECRETION -
BIOSYNTHESIS OF AMINO ACIDS -
BIOSYNTHESIS OF UNSATURATED FATTY ACIDS -
BIOTIN METABOLISM -
BLADDER CANCER -
BUTANOATE METABOLISM -
BUTIROSIN AND NEOMYCIN BIOSYNTHESIS -
CAFFEINE METABOLISM -
CALCIUM SIGNALING PATHWAY -
CAMP SIGNALING PATHWAY -
CARBOHYDRATE DIGESTION AND ABSORPTION -
CARBON METABOLISM -
CARDIAC MUSCLE CONTRACTION -
CELL ADHESION MOLECULES (CAMS) -
p-values
0.0
0.4
0.8
0.0
0.2
0.4
0.6
0.8
1.0
Theoretical quantiles

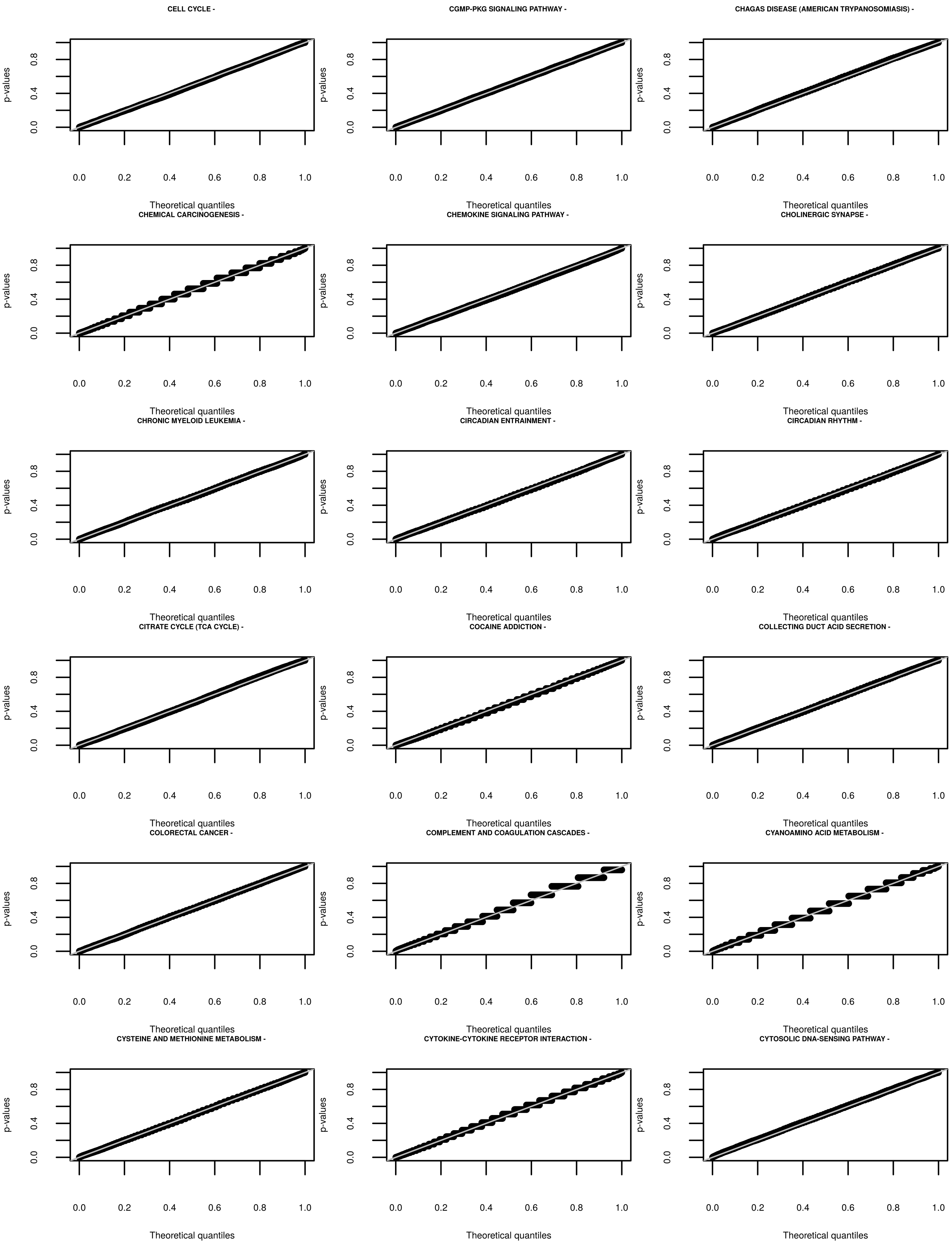
CELL CYCLE -
CGMP-PKG SIGNALING PATHWAY -
CHAGAS DISEASE (AMERICAN TRYPANOSOMIASIS) -
CHEMICAL CARCINOGENESIS -
CHEMOKINE SIGNALING PATHWAY -
CHOLINERGIC SYNAPSE -
CHRONIC MYELOID LEUKEMIA -
CIRCADIAN ENTRAINMENT -
CIRCADIAN RHYTHM -
CITRATE CYCLE (TCA CYCLE) -
COCAINE ADDICTION -
COLLECTING DUCT ACID SECRETION -
COLORECTAL CANCER -
COMPLEMENT AND COAGULATION CASCADES -
CYANOAMINO ACID METABOLISM -
CYSTEINE AND METHIONINE METABOLISM -
CYTOKINE-CYTOKINE RECEPTOR INTERACTION -
CYTOSOLIC DNA-SENSING PATHWAY -
p-values
0.0
0.4
0.8
0.0
0.2
0.4
0.6
0.8
1.0
Theoretical quantiles

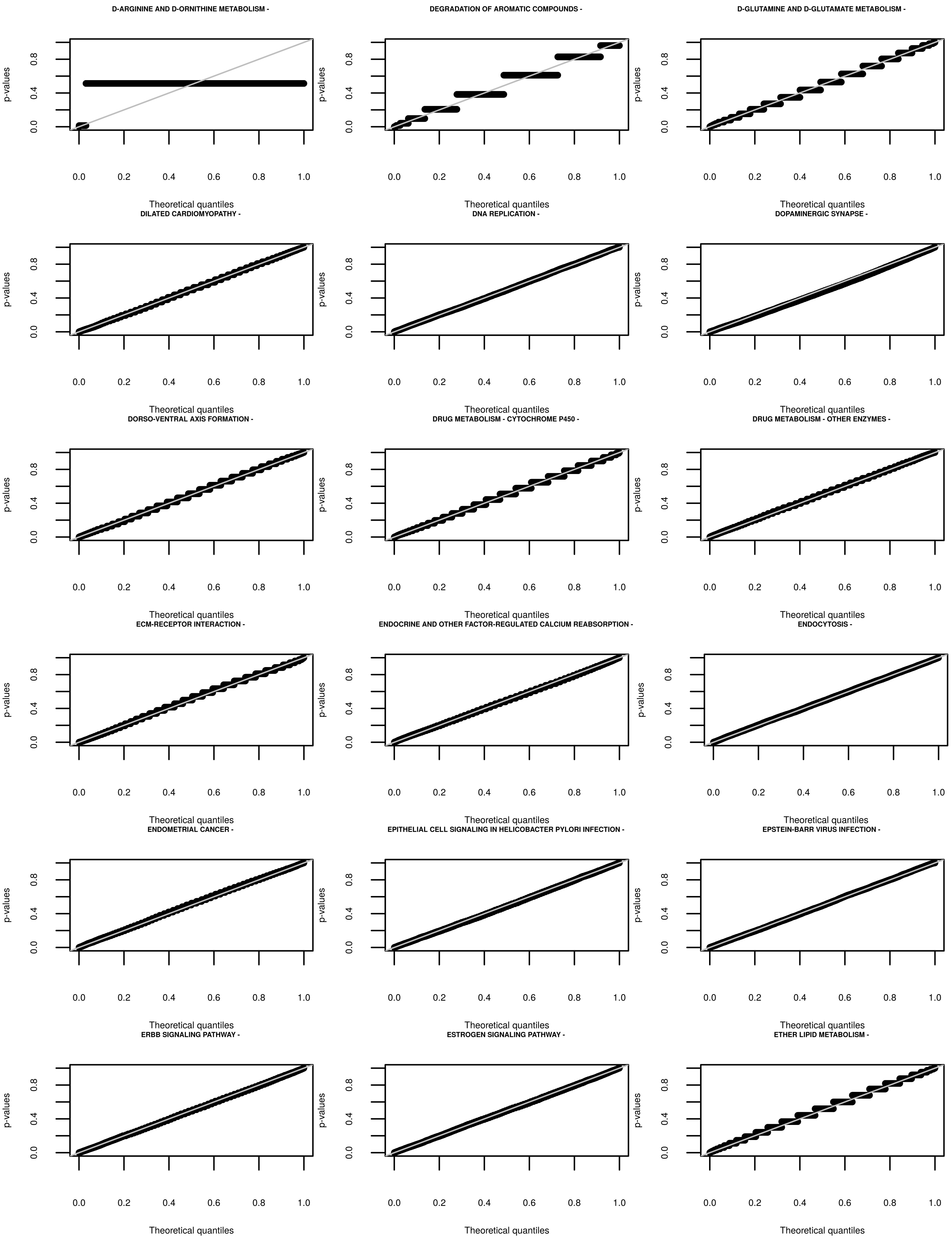
D-ARGININE AND D-ORNITHINE METABOLISM -
DEGRADATION OF AROMATIC COMPOUNDS -
D-GLUTAMINE AND D-GLUTAMATE METABOLISM -
DILATED CARDIOMYOPATHY -
DNA REPLICATION -
DOPAMINERGIC SYNAPSE -
DORSO-VENTRAL AXIS FORMATION -
DRUG METABOLISM - CYTOCHROME P450 -
DRUG METABOLISM - OTHER ENZYMES -
ECM-RECEPTOR INTERACTION -
ENDOCRINE AND OTHER FACTOR-REGULATED CALCIUM REABSORPTION -
ENDOCYTOSIS -
ENDOMETRIAL CANCER -
EPITHELIAL CELL SIGNALING IN HELICOBACTER PYLORI INFECTION -
EPSTEIN-BARR VIRUS INFECTION -
ERBB SIGNALING PATHWAY -
ESTROGEN SIGNALING PATHWAY -
ETHER LIPID METABOLISM -
p-values
0.0
0.4
0.8
Theoretical quantiles
0.0
0.2
0.4
0.6
0.8
1.0

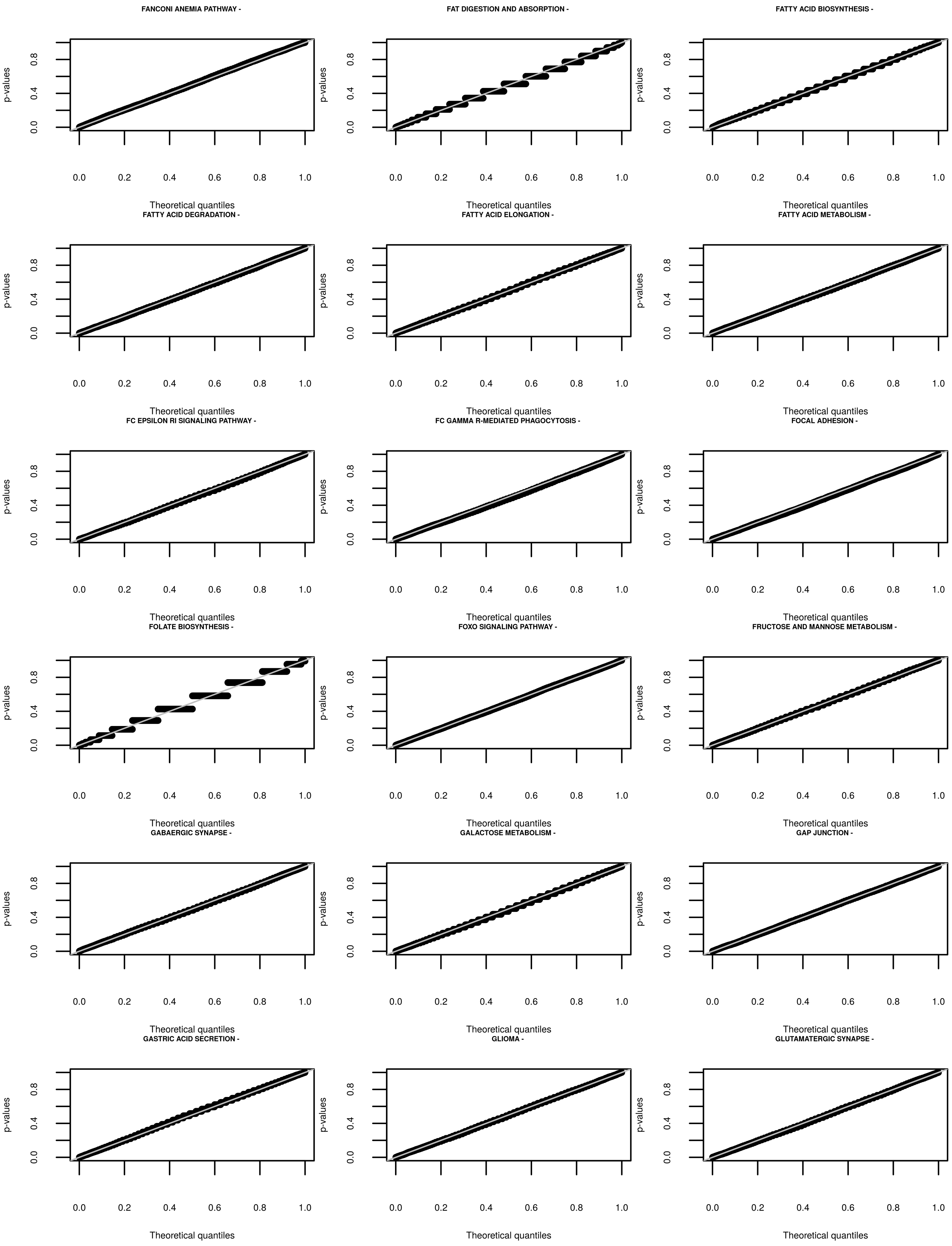
FANCONI ANEMIA PATHWAY -
FAT DIGESTION AND ABSORPTION -
FATTY ACID BIOSYNTHESIS -
FATTY ACID DEGRADATION -
FATTY ACID ELONGATION -
FATTY ACID METABOLISM -
FC EPSILON RI SIGNALING PATHWAY -
FC GAMMA R-MEDIATED PHAGOCYTOSIS -
FOCAL ADHESION -
FOLATE BIOSYNTHESIS -
FOXO SIGNALING PATHWAY -
FRUCTOSE AND MANNOSE METABOLISM -
GABAERGIC SYNAPSE -
GALACTOSE METABOLISM -
GAP JUNCTION -
GASTRIC ACID SECRETION -
GLIOMA -
GLUTAMATERGIC SYNAPSE -
p-values
0.0
0.4
0.8
Theoretical quantiles
0.0
0.2
0.4
0.6
0.8
1.0

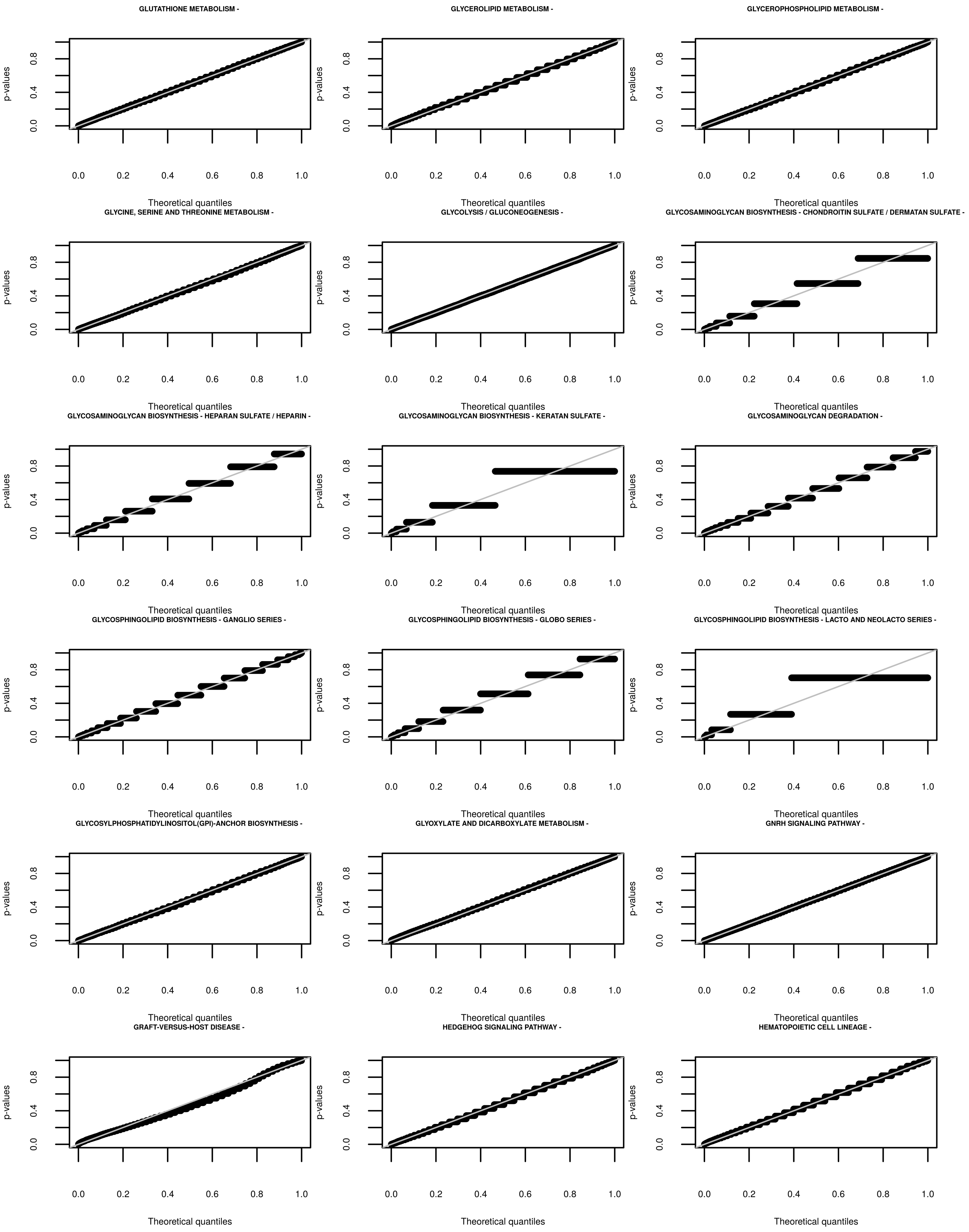
GLUTATHIONE METABOLISM -
GLYCEROLIPID METABOLISM -
GLYCEROPHOSPHOLIPID METABOLISM -
GLYCINE, SERINE AND THREONINE METABOLISM -
GLYCOLYSIS / GLUCONEOGENESIS -
GLYCOSAMINOGLYCAN BIOSYNTHESIS - CHONDROITIN SULFATE / DERMATAN SULFATE -
GLYCOSAMINOGLYCAN BIOSYNTHESIS - HEPARAN SULFATE / HEPARIN -
GLYCOSAMINOGLYCAN BIOSYNTHESIS - KERATAN SULFATE -
GLYCOSAMINOGLYCAN DEGRADATION -
GLYCOSPHINGOLIPID BIOSYNTHESIS - GANGLIO SERIES -
GLYCOSPHINGOLIPID BIOSYNTHESIS - GLOBO SERIES -
GLYCOSPHINGOLIPID BIOSYNTHESIS - LACTO AND NEOLACTO SERIES -
GLYCOSYLPHOSPHATIDYLINOSITOL(GPI)-ANCHOR BIOSYNTHESIS -
GLYOXYLATE AND DICARBOXYLATE METABOLISM -
GNRH SIGNALING PATHWAY -
GRAFT-VERSUS-HOST DISEASE -
HEDGEHOG SIGNALING PATHWAY -
HEMATOPOIETIC CELL LINEAGE -
p-values
0.0
0.4
0.8
Theoretical quantiles
0.0
0.2
0.4
0.6
0.8
1.0

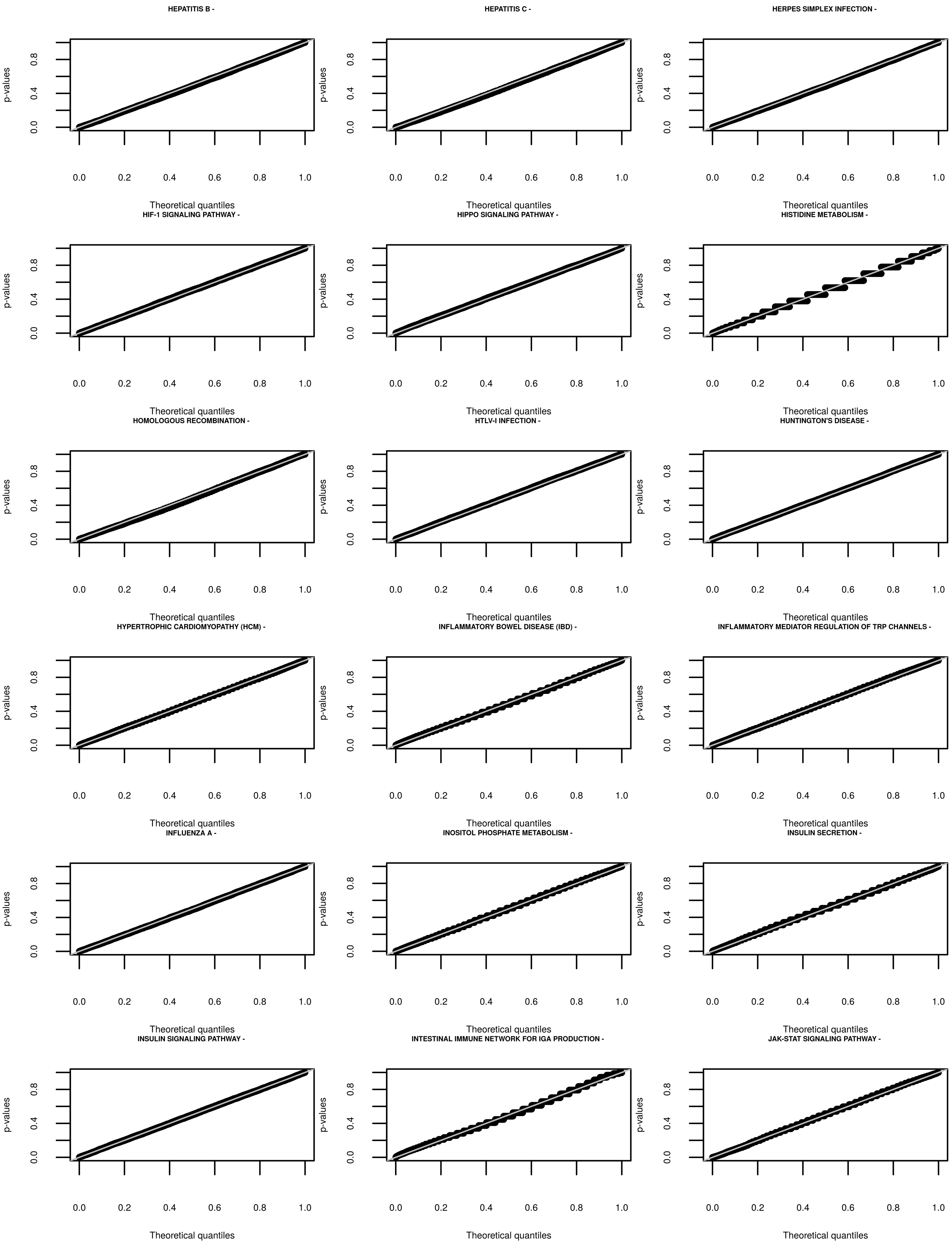
HEPATITIS B -
HEPATITIS C -
HERPES SIMPLEX INFECTION -
HIF-1 SIGNALING PATHWAY -
HIPPO SIGNALING PATHWAY -
HISTIDINE METABOLISM -
HOMOLOGOUS RECOMBINATION -
HTLV-I INFECTION -
HUNTINGTON'S DISEASE -
HYPERTROPHIC CARDIOMYOPATHY (HCM) -
INFLAMMATORY BOWEL DISEASE (IBD) -
INFLAMMATORY MEDIATOR REGULATION OF TRP CHANNELS -
INFLUENZA A -
INOSITOL PHOSPHATE METABOLISM -
INSULIN SECRETION -
INSULIN SIGNALING PATHWAY -
INTESTINAL IMMUNE NETWORK FOR IGA PRODUCTION -
JAK-STAT SIGNALING PATHWAY -
p-values
0.0
0.4
0.8
Theoretical quantiles
0.0
0.2
0.4
0.6
0.8
1.0

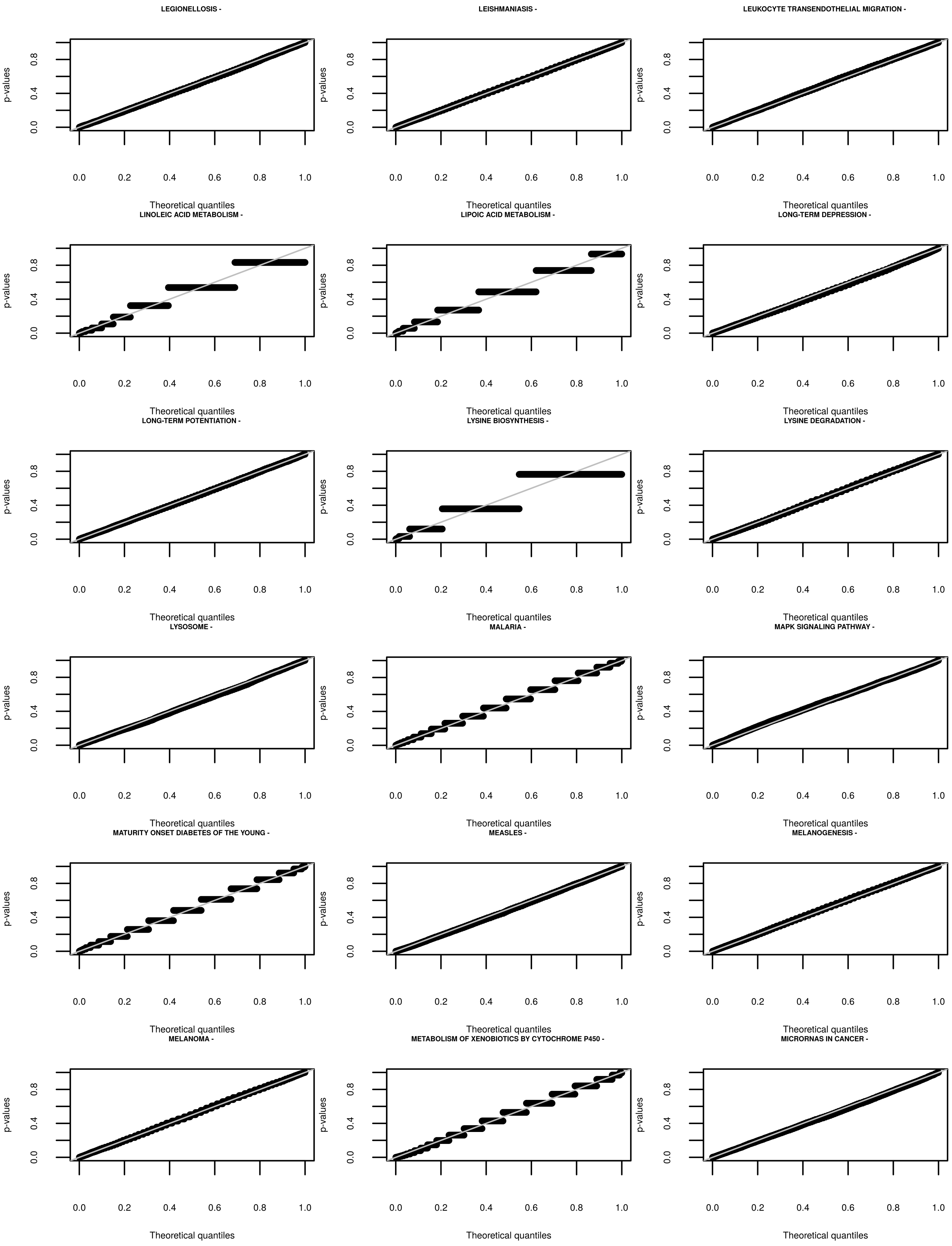
LEGIONELLOSIS -
LEISHMANIASIS -
LEUKOCYTE TRANSENDOTHELIAL MIGRATION -
LINOLEIC ACID METABOLISM -
LIPOIC ACID METABOLISM -
LONG-TERM DEPRESSION -
LONG-TERM POTENTIATION -
LYSINE BIOSYNTHESIS -
LYSINE DEGRADATION -
LYSOSOME -
MALARIA -
MAPK SIGNALING PATHWAY -
MATURITY ONSET DIABETES OF THE YOUNG -
MEASLES -
MELANOGENESIS -
MELANOMA -
METABOLISM OF XENOBIOTICS BY CYTOCHROME P450 -
MICRORNAS IN CANCER -
p-values
0.0
0.4
0.8
Theoretical quantiles
0.0
0.2
0.4
0.6
0.8
1.0

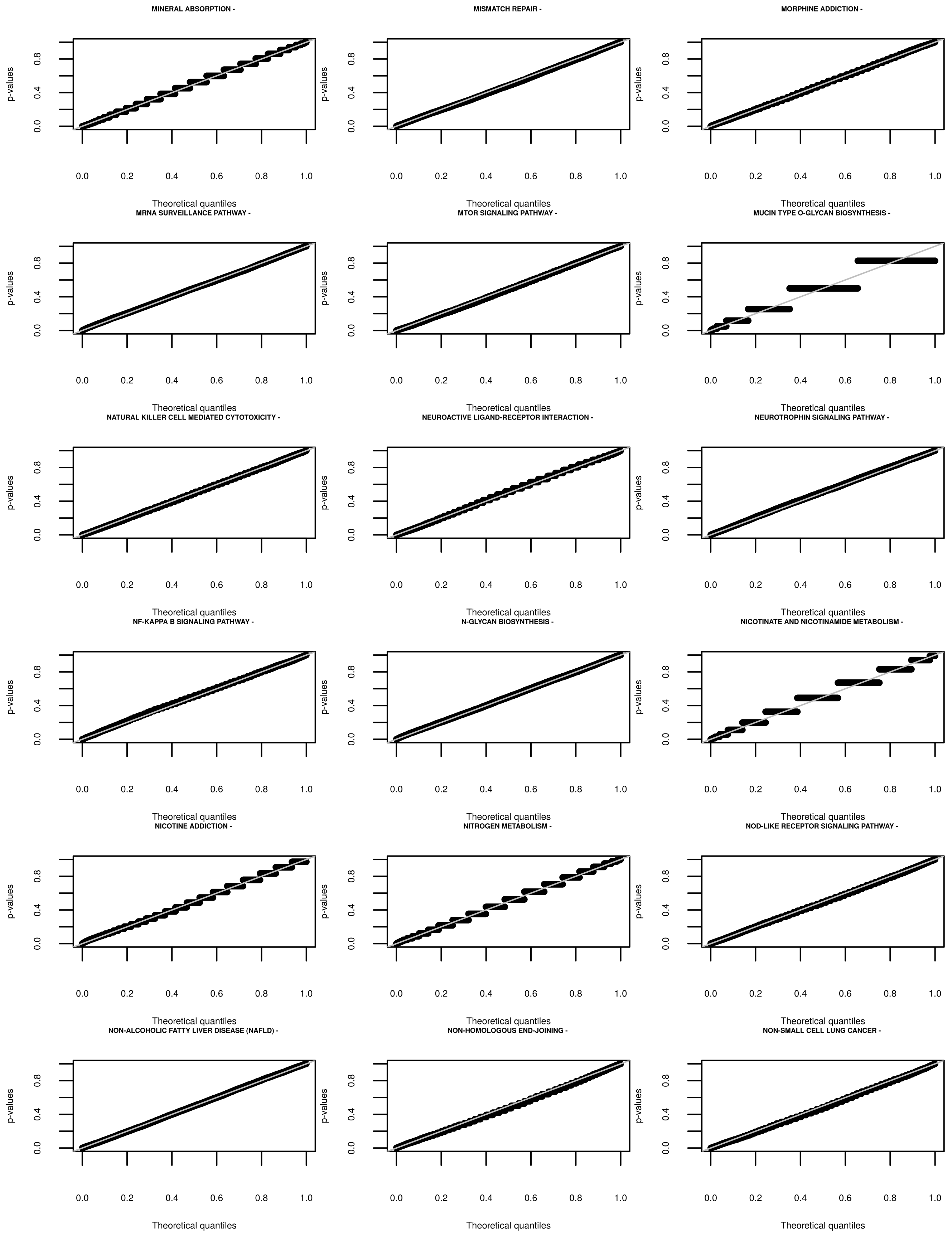
MINERAL ABSORPTION -
MISMATCH REPAIR -
MORPHINE ADDICTION -
MRNA SURVEILLANCE PATHWAY -
MTOR SIGNALING PATHWAY -
MUCIN TYPE O-GLYCAN BIOSYNTHESIS -
NATURAL KILLER CELL MEDIATED CYTOTOXICITY -
NEUROACTIVE LIGAND-RECEPTOR INTERACTION -
NEUROTROPHIN SIGNALING PATHWAY -
NF-KAPPA B SIGNALING PATHWAY -
N-GLYCAN BIOSYNTHESIS -
NICOTINATE AND NICOTINAMIDE METABOLISM -
NICOTINE ADDICTION -
NITROGEN METABOLISM -
NOD-LIKE RECEPTOR SIGNALING PATHWAY -
NON-ALCOHOLIC FATTY LIVER DISEASE (NAFLD) -
NON-HOMOLOGOUS END-JOINING -
NON-SMALL CELL LUNG CANCER -
p-values
0.0
0.4
0.8
Theoretical quantiles
0.0
0.2
0.4
0.6
0.8
1.0

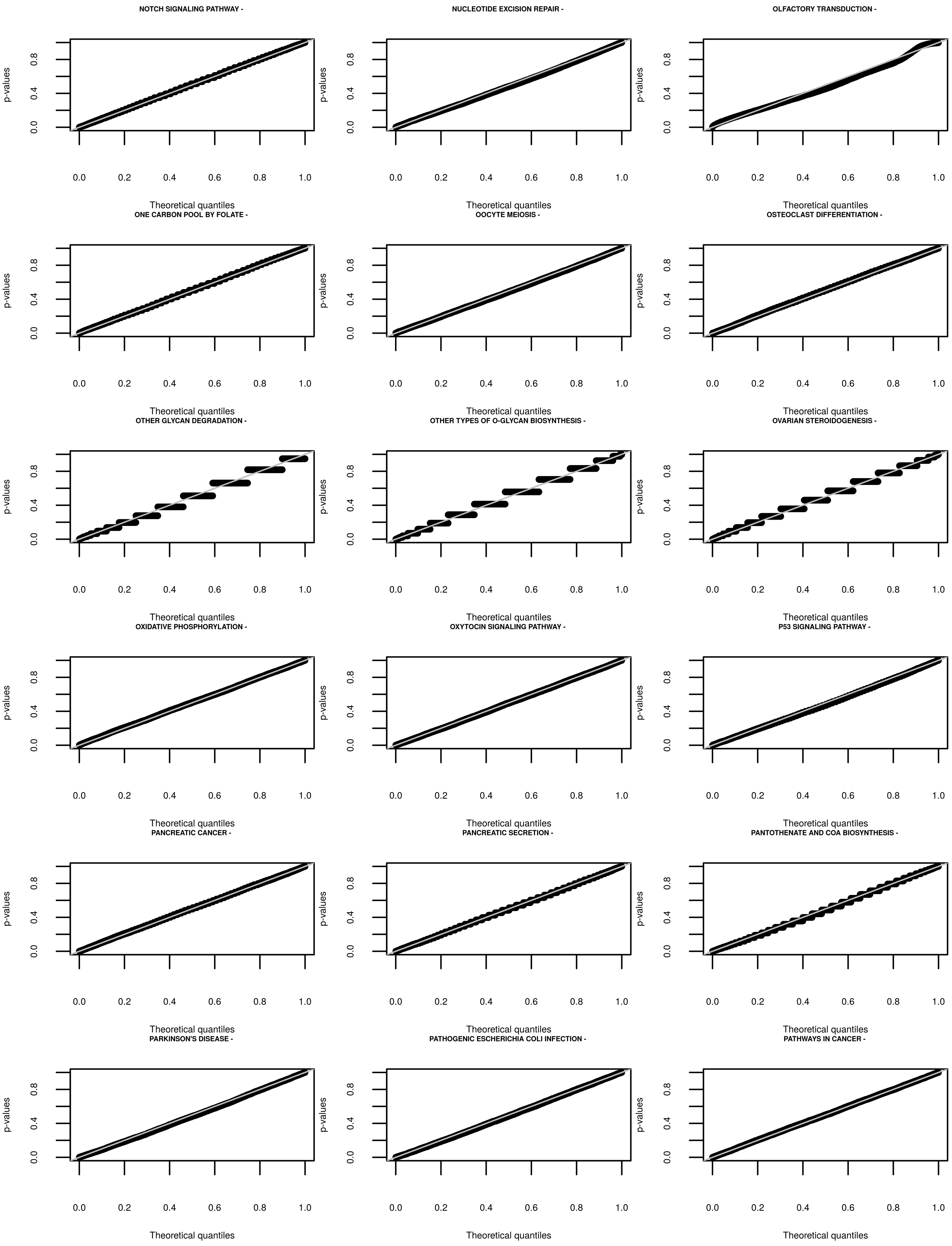
NOTCH SIGNALING PATHWAY -
NUCLEOTIDE EXCISION REPAIR -
OLFACTORY TRANSDUCTION -
ONE CARBON POOL BY FOLATE -
OOCYTE MEIOSIS -
OSTEOCLAST DIFFERENTIATION -
OTHER GLYCAN DEGRADATION -
OTHER TYPES OF O-GLYCAN BIOSYNTHESIS -
OVARIAN STEROIDOGENESIS -
OXIDATIVE PHOSPHORYLATION -
OXYTOCIN SIGNALING PATHWAY -
P53 SIGNALING PATHWAY -
PANCREATIC CANCER -
PANCREATIC SECRETION -
PANTOTHENATE AND COA BIOSYNTHESIS -
PARKINSON'S DISEASE -
PATHOGENIC ESCHERICHIA COLI INFECTION -
PATHWAYS IN CANCER -
p-values
0.0
0.4
0.8
Theoretical quantiles
0.0
0.2
0.4
0.6
0.8
1.0

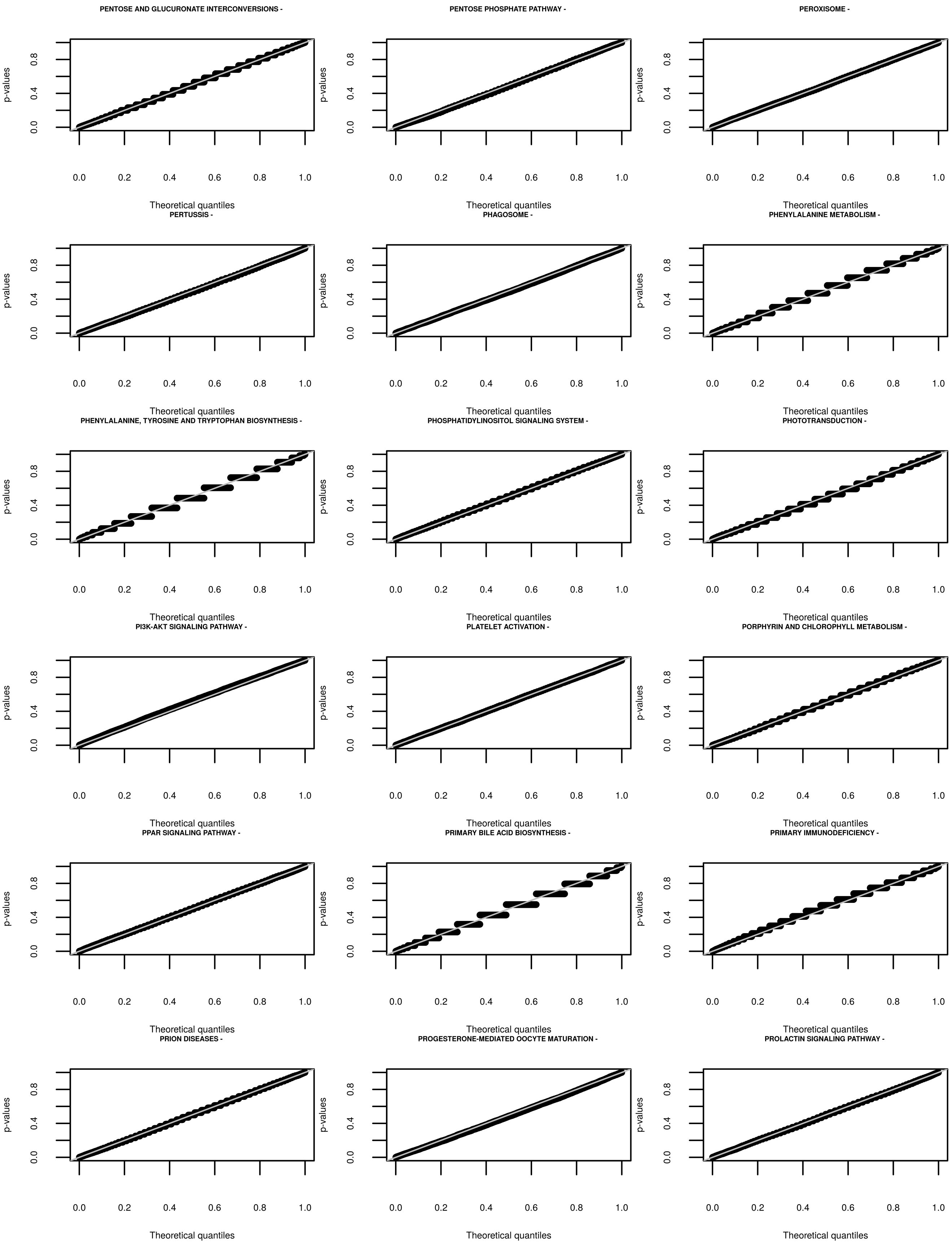
PENTOSE AND GLUCURONATE INTERCONVERSIONS -
PENTOSE PHOSPHATE PATHWAY -
PEROXISOME -
PERTUSSIS -
PHAGOSOME -
PHENYLALANINE METABOLISM -
PHENYLALANINE, TYROSINE AND TRYPTOPHAN BIOSYNTHESIS -
PHOSPHATIDYLINOSITOL SIGNALING SYSTEM -
PHOTOTRANSDUCTION -
PI3K-AKT SIGNALING PATHWAY -
PLATELET ACTIVATION -
PORPHYRIN AND CHLOROPHYLL METABOLISM -
PPAR SIGNALING PATHWAY -
PRIMARY BILE ACID BIOSYNTHESIS -
PRIMARY IMMUNODEFICIENCY -
PRION DISEASES -
PROGESTERONE-MEDIATED OOCYTE MATURATION -
PROLACTIN SIGNALING PATHWAY -
p-values
0.0
0.4
0.8
0.0
0.2
0.4
0.6
0.8
1.0
Theoretical quantiles

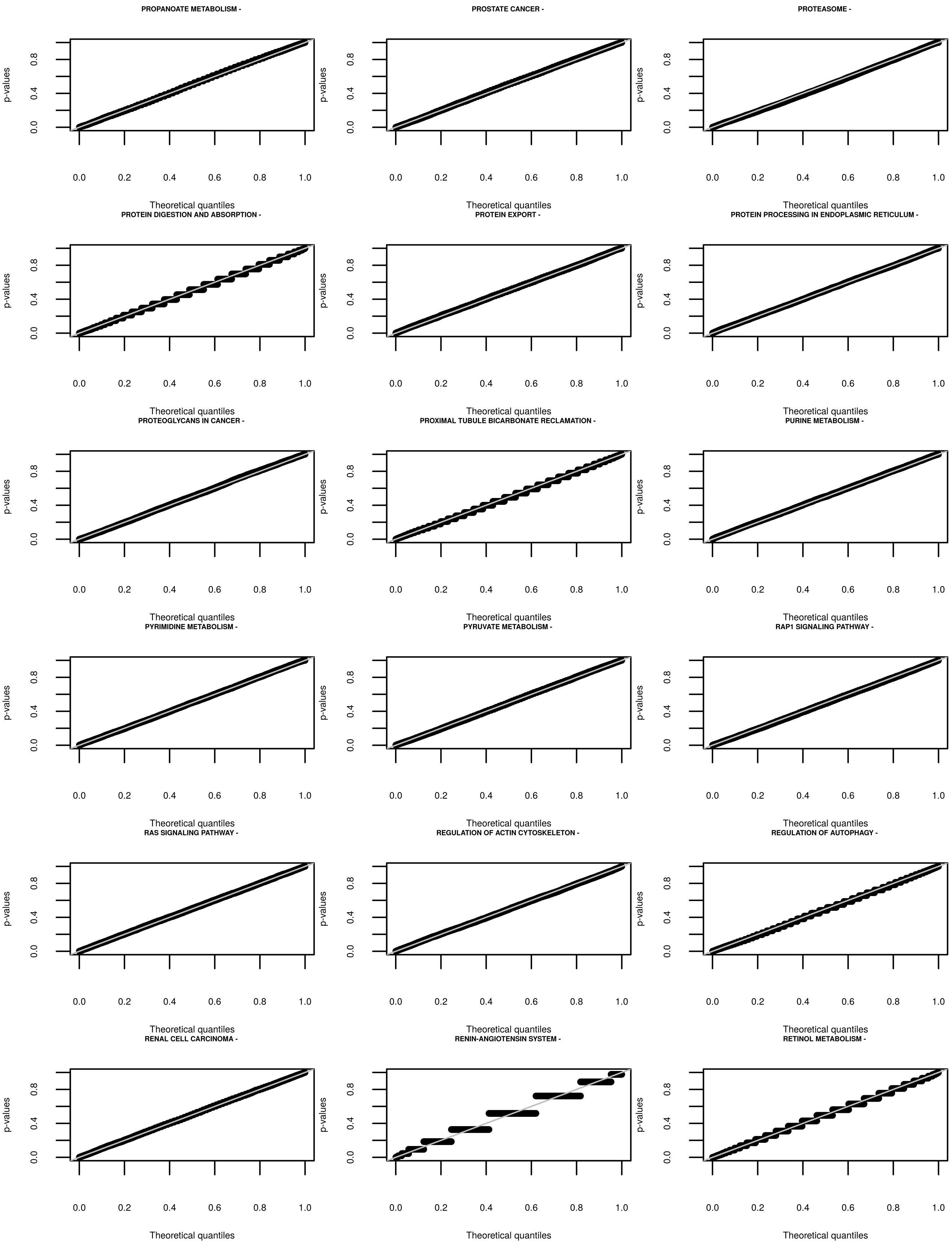
PROPANOATE METABOLISM -
p-values
0.0 0.4 0.8
0.0 0.2 0.4 0.6 0.8 1.0
Theoretical quantiles
PROSTATE CANCER -
p-values
0.0 0.4 0.8
0.0 0.2 0.4 0.6 0.8 1.0
Theoretical quantiles
PROTEASOME -
p-values
0.0 0.4 0.8
0.0 0.2 0.4 0.6 0.8 1.0
Theoretical quantiles
PROTEIN DIGESTION AND ABSORPTION -
p-values
0.0 0.4 0.8
0.0 0.2 0.4 0.6 0.8 1.0
Theoretical quantiles
PROTEIN EXPORT -
p-values
0.0 0.4 0.8
0.0 0.2 0.4 0.6 0.8 1.0
Theoretical quantiles
PROTEIN PROCESSING IN ENDOPLASMIC RETICULUM -
p-values
0.0 0.4 0.8
0.0 0.2 0.4 0.6 0.8 1.0
Theoretical quantiles
PROTEOGLYCANS IN CANCER -
p-values
0.0 0.4 0.8
0.0 0.2 0.4 0.6 0.8 1.0
Theoretical quantiles
PROXIMAL TUBULE BICARBONATE RECLAMATION -
p-values
0.0 0.4 0.8
0.0 0.2 0.4 0.6 0.8 1.0
Theoretical quantiles
PURINE METABOLISM -
p-values
0.0 0.4 0.8
0.0 0.2 0.4 0.6 0.8 1.0
Theoretical quantiles
PYRIMIDINE METABOLISM -
p-values
0.0 0.4 0.8
0.0 0.2 0.4 0.6 0.8 1.0
Theoretical quantiles
PYRUVATE METABOLISM -
p-values
0.0 0.4 0.8
0.0 0.2 0.4 0.6 0.8 1.0
Theoretical quantiles
RAP1 SIGNALING PATHWAY -
p-values
0.0 0.4 0.8
0.0 0.2 0.4 0.6 0.8 1.0
Theoretical quantiles
RAS SIGNALING PATHWAY -
p-values
0.0 0.4 0.8
0.0 0.2 0.4 0.6 0.8 1.0
Theoretical quantiles
REGULATION OF ACTIN CYTOSKELETON -
p-values
0.0 0.4 0.8
0.0 0.2 0.4 0.6 0.8 1.0
Theoretical quantiles
REGULATION OF AUTOPHAGY -
p-values
0.0 0.4 0.8
0.0 0.2 0.4 0.6 0.8 1.0
Theoretical quantiles
RENAL CELL CARCINOMA -
p-values
0.0 0.4 0.8
0.0 0.2 0.4 0.6 0.8 1.0
Theoretical quantiles
RENIN-ANGIOTENSIN SYSTEM -
p-values
0.0 0.4 0.8
0.0 0.2 0.4 0.6 0.8 1.0
Theoretical quantiles
RETINOL METABOLISM -
p-values
0.0 0.4 0.8
0.0 0.2 0.4 0.6 0.8 1.0
Theoretical quantiles

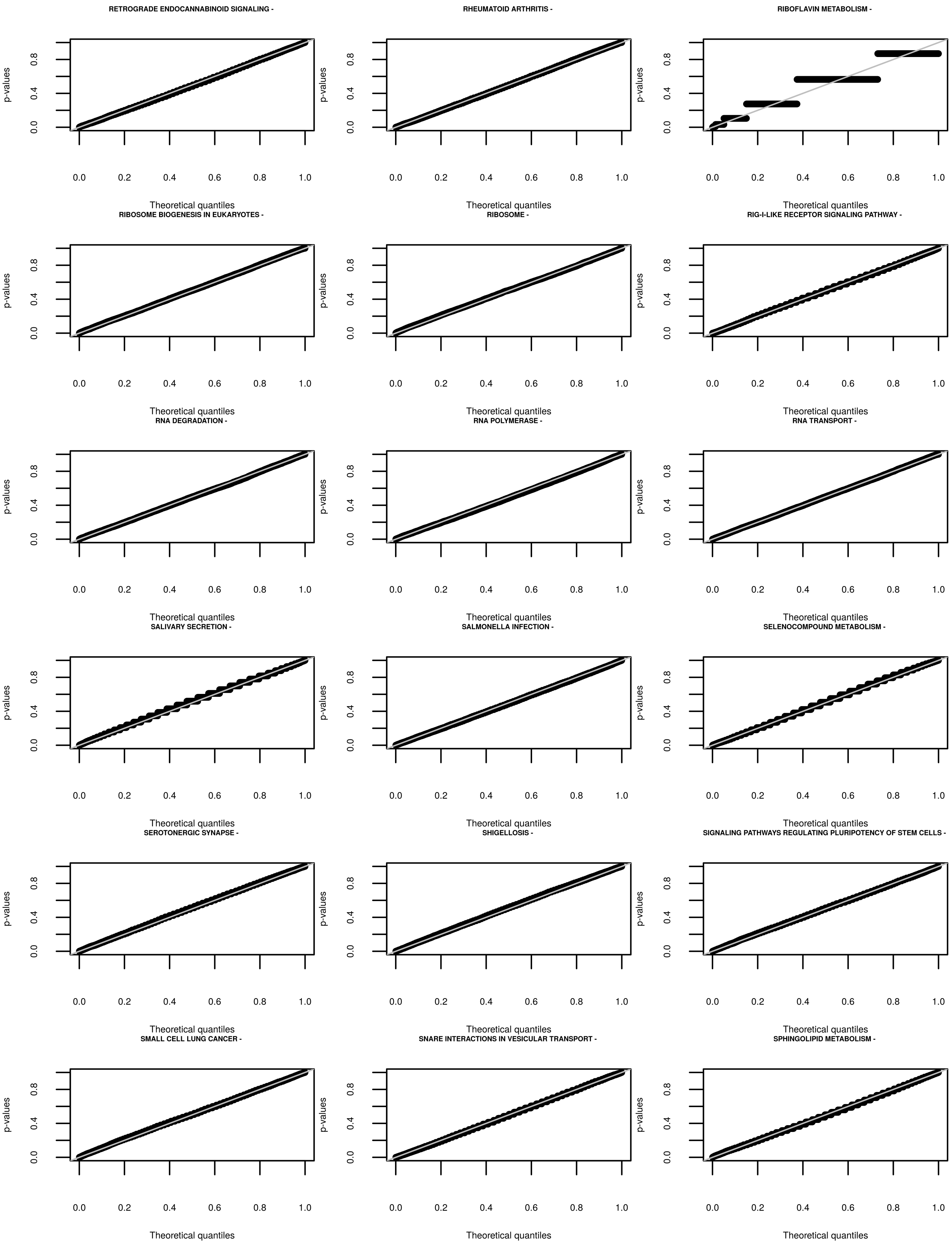
RETROGRADE ENDOCANNABINOID SIGNALING -
RHEUMATOID ARTHRITIS -
RIBOFLAVIN METABOLISM -
RIBOSOME BIOGENESIS IN EUKARYOTES -
RIBOSOME -
RIG-I-LIKE RECEPTOR SIGNALING PATHWAY -
RNA DEGRADATION -
RNA POLYMERASE -
RNA TRANSPORT -
SALIVARY SECRETION -
SALMONELLA INFECTION -
SELENOCOMPOUND METABOLISM -
SEROTONERGIC SYNAPSE -
SHIGELLOSIS -
SIGNALING PATHWAYS REGULATING PLURIPOTENCY OF STEM CELLS -
SMALL CELL LUNG CANCER -
SNARE INTERACTIONS IN VESICULAR TRANSPORT -
SPHINGOLIPID METABOLISM -
p-values
0.0
0.4
0.8
0.0
0.2
0.4
0.6
0.8
1.0
Theoretical quantiles

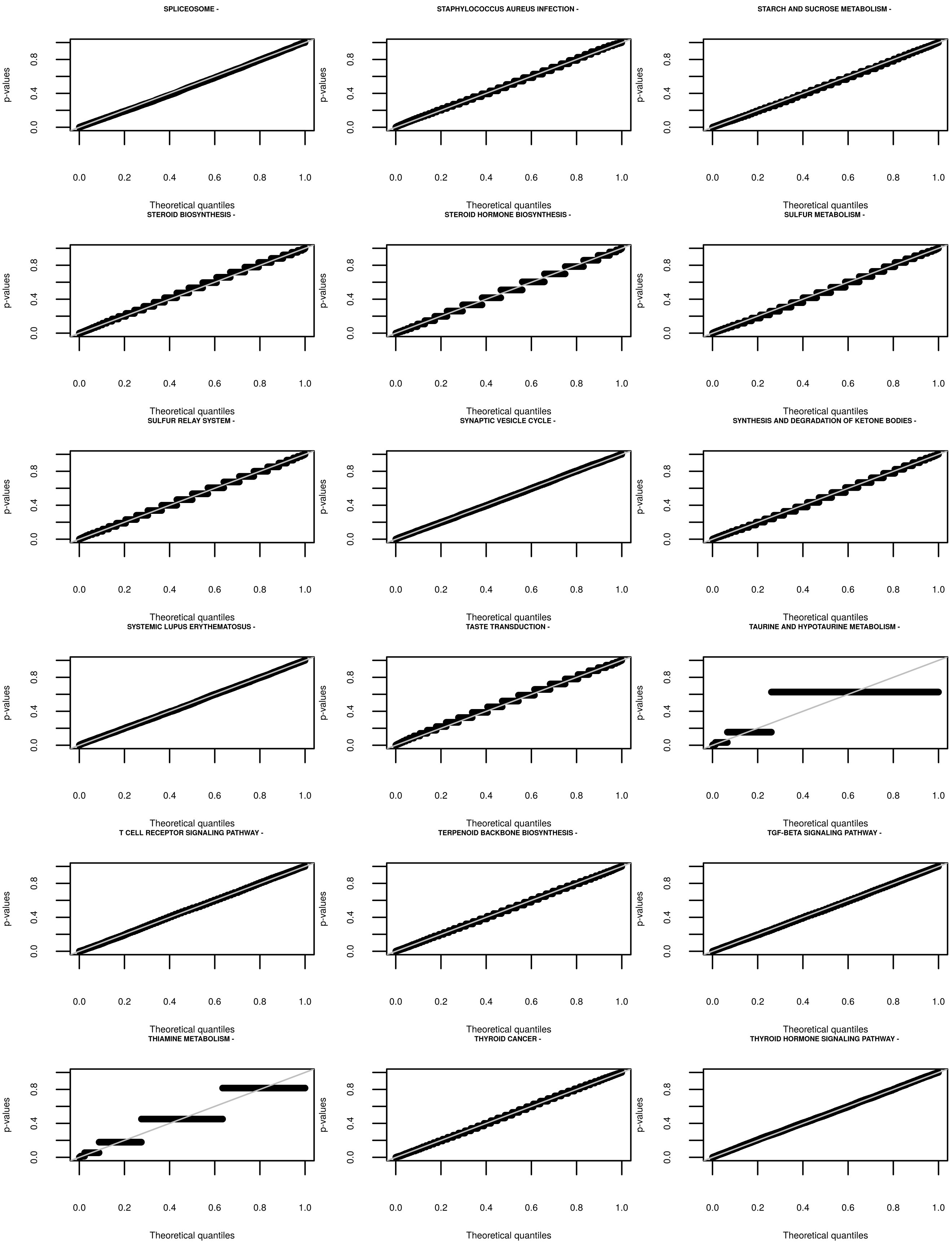
SPLICEOSOME -
STAPHYLOCOCCUS AUREUS INFECTION -
STARCH AND SUCROSE METABOLISM -
STEROID BIOSYNTHESIS -
STEROID HORMONE BIOSYNTHESIS -
SULFUR METABOLISM -
SULFUR RELAY SYSTEM -
SYNAPTIC VESICLE CYCLE -
SYNTHESIS AND DEGRADATION OF KETONE BODIES -
SYSTEMIC LUPUS ERYTHEMATOSUS -
TASTE TRANSDUCTION -
TAURINE AND HYPOTAURINE METABOLISM -
T CELL RECEPTOR SIGNALING PATHWAY -
TERPENOID BACKBONE BIOSYNTHESIS -
TGF-BETA SIGNALING PATHWAY -
THIAMINE METABOLISM -
THYROID CANCER -
THYROID HORMONE SIGNALING PATHWAY -
p-values
0.0
0.4
0.8
Theoretical quantiles
0.0
0.2
0.4
0.6
0.8
1.0

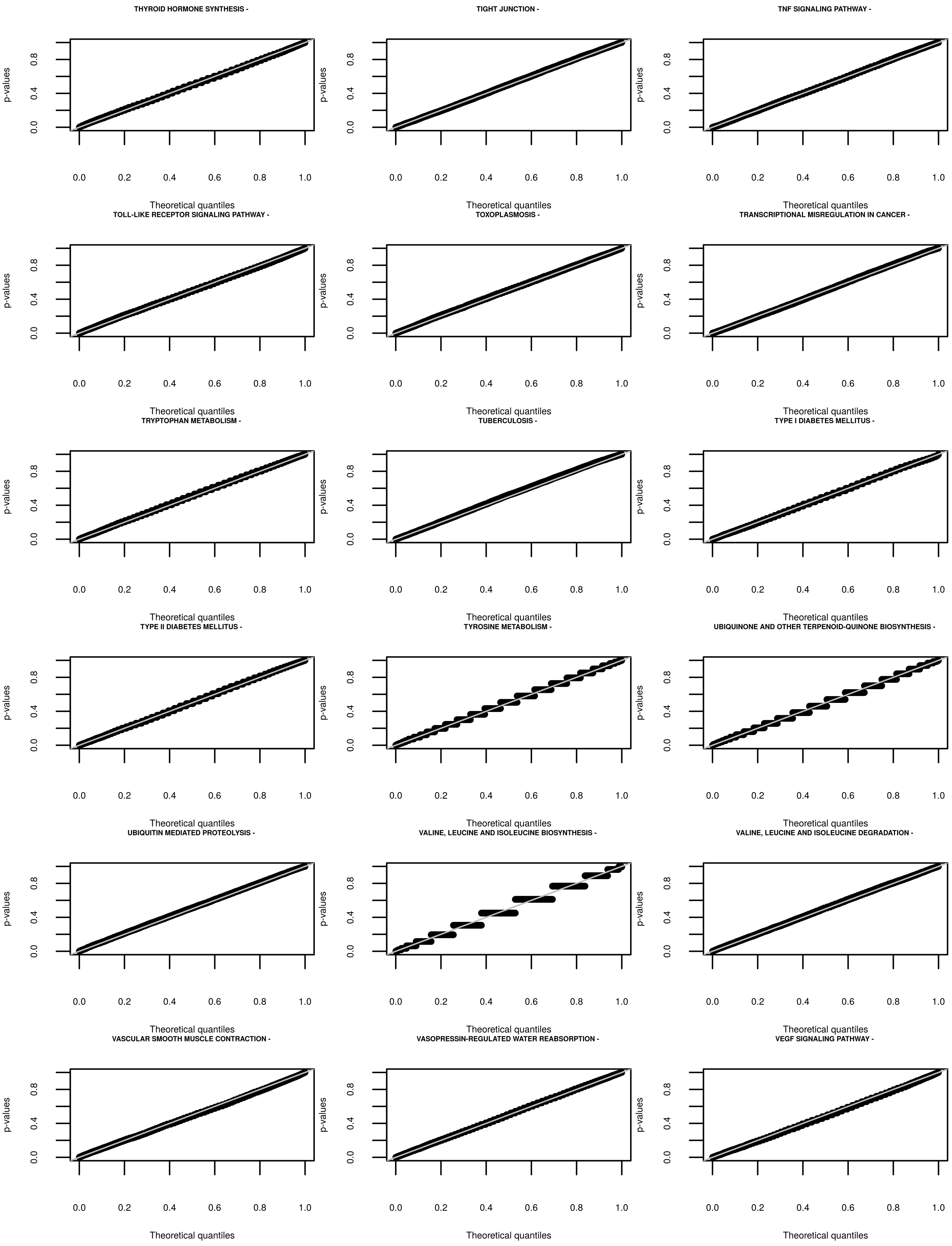
THYROID HORMONE SYNTHESIS -
TIGHT JUNCTION -
TNF SIGNALING PATHWAY -
TOLL-LIKE RECEPTOR SIGNALING PATHWAY -
TOXOPLASMOSIS -
TRANSCRIPTIONAL MISREGULATION IN CANCER -
TRYPTOPHAN METABOLISM -
TUBERCULOSIS -
TYPE I DIABETES MELLITUS -
TYPE II DIABETES MELLITUS -
TYROSINE METABOLISM -
UBIQUINONE AND OTHER TERPENOID-QUINONE BIOSYNTHESIS -
UBIQUITIN MEDIATED PROTEOLYSIS -
VALINE, LEUCINE AND ISOLEUCINE BIOSYNTHESIS -
VALINE, LEUCINE AND ISOLEUCINE DEGRADATION -
VASCULAR SMOOTH MUSCLE CONTRACTION -
VASOPRESSIN-REGULATED WATER REABSORPTION -
VEGF SIGNALING PATHWAY -
p-values
0.0
0.4
0.8
0.0
0.2
0.4
0.6
0.8
1.0
Theoretical quantiles

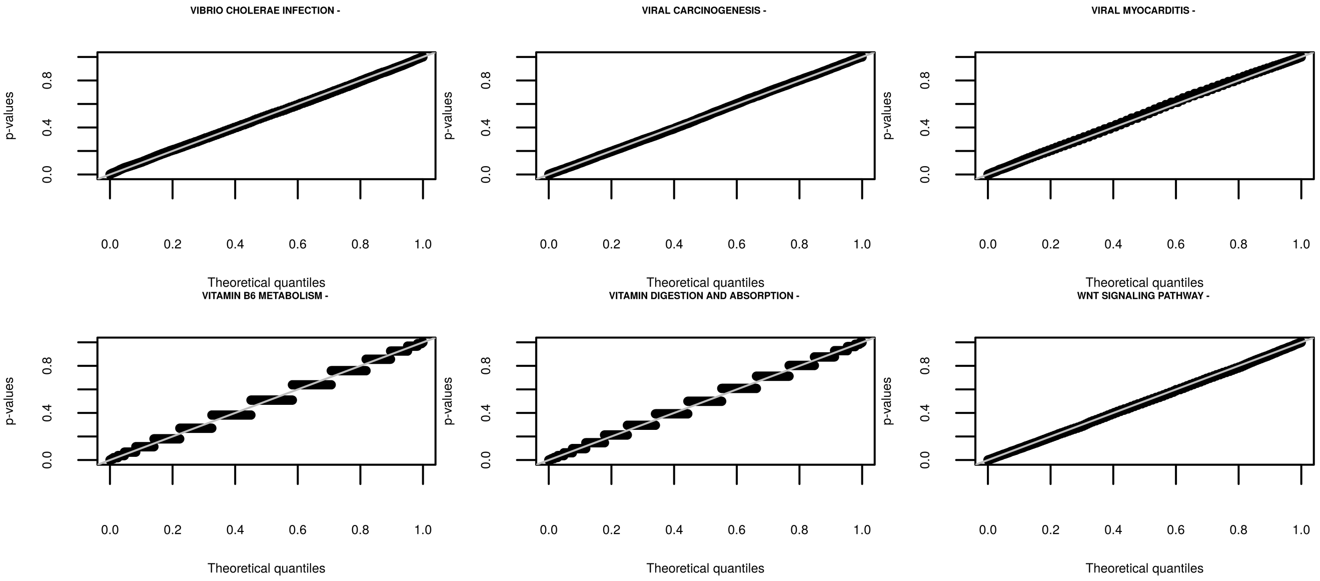
VIBRIO CHOLERAE INFECTION -
VIRAL CARCINOGENESIS -
VIRAL MYOCARDITIS -
VITAMIN B6 METABOLISM -
VITAMIN DIGESTION AND ABSORPTION -
WNT SIGNALING PATHWAY -
p-values
0.0
0.4
0.8
0.0
0.2
0.4
0.6
0.8
1.0
Theoretical quantiles
